# Children's and Adolescents’ Actual Motor Competence, Perceived Physical Competence and Physical Activity: A Structural Equation Modelling Meta-Analysis

**DOI:** 10.1007/s40279-025-02233-2

**Published:** 2025-05-06

**Authors:** Matthew Bourke, Hiu Fei Wendy Wang, Hughston Wicks, Lisa M. Barnett, John Cairney, Kathryn Fortnum

**Affiliations:** 1 The Health and Wellbeing Centre for Research Innovation, School of Human Movement and Nutrition Sciences, The University of Queensland, Brisbane, QLD Australia; 2 School of Human Movement and Nutrition Sciences, The University of Queensland, Brisbane, QLD Australia; 3 Institute for Physical Activity and Nutrition (IPAN), School of Exercise and Nutrition Sciences, Deakin University, Burwood, VIC Australia

## Abstract

**Background:**

Perceived physical competence (e.g. perceived motor skills, perceived athletic competence) is hypothesised to mediate the association between actual motor competence and physical activity in children, and this mediated association is expected to be stronger in older children and adolescents. However, no meta-analyses to date have synthesised the hypothesised mediation effect.

**Objectives:**

The purpose of this study was to systematically identify and synthesise the existing literature on the hypothesised mediation model between actual motor competence, perceived physical competence and physical activity in children and adolescents using structural equation modelling meta-analysis.

**Methods:**

Five electronic databases were searched from inception to December 2023 using a range of keywords for actual motor competence, perceived physical competence, physical activity and children/adolescents. Machine learning assisted screening was used to identify studies which reported the association between at least two of the variables in the hypothesised model in children and adolescents aged 4–18 years. One-stage structural equation modelling meta-analysis was used to test the hypothesised model. Moderation analysis was conducted to determine whether any of the model parameters differed as a function of children’s age.

**Results:**

A total of 218 reports that reported on 213 studies met the inclusion criteria and were included in the meta-analysis. Results from studies which examined the concurrent association between actual motor competence (gross motor, locomotion, object control), perceived physical competence, and physical activity demonstrated that perceived physical competence only had a small absolute (0.029 ≤ *r* ≤ 0.034) and relative (16.7–20.6% of total effect) mediating effect on the association between actual motor competence and physical activity. Results from studies which examined lagged associations (11% of included studies) also demonstrated a small bidirectional mediation effect of perceived physical competence. The moderation model demonstrated the mediation effect was significantly stronger in adolescents than children, albeit still weak.

**Conclusions:**

Perceived physical competence is not a strong mediator of the association between actual motor competence and physical activity in children and adolescents. Given that the association between perceived physical competence and actual motor competence with physical activity are largely independent, there may be benefits to targeting both motor skills and perceived physical competence to increase engagement in physical activity.

**Supplementary Information:**

The online version contains supplementary material available at 10.1007/s40279-025-02233-2.

## Key Points

**Table:** 

Perceived physical competence is hypothesised to mediate the association between actual motor competence and physical activity.
Structural equation modelling meta-analysis was used to evaluate this hypothesis across 213 studies and more than 60,000 children and adolescents.
Perceived physical competence only had a small absolute and relative mediating effect on the relationship between actual motor competence and physical activity.

## Background

Regular engagement in physical activity is associated with a range of physical, mental, social and cognitive health benefits in children and adolescents [1, 2]. Given the range and magnitude of the health benefits of regular engagement in physical activity, increasing population levels of physical activity has been recognised as a global public health priority [3]. The World Health Organization Global Action Plan sets out a global target of a relative reduction in the number of adolescents (aged 11–17 years) who do not engage in an average 60 min of moderate-to-vigorous intensity physical activity (MVPA) each day by 15% from 2018 to 2030 [4]. Although no global targets exist for children, levels of physical activity decrease substantially year-on-year as children grow older [5]. It is therefore crucial to provide children with the necessary building blocks to ensure they can continually engage in health enhancing levels of physical activity.

Researchers frequently recognise the importance of motor competence as an antecedent of physical activity in children and adolescents [6–8]. Motor competence describes the degree of mastery an individual has in completing goal-oriented movements and includes different skill components, such as locomotor skills (e.g. hopping on one leg, running) and object control skills (e.g. throwing, or kicking a ball) [9]. Motor competence may also include the underlying mechanisms necessary to complete goal-oriented movements, such as motor coordination [6]. Mastering basic motor competencies in childhood may lead to progression onto more specialised, sport-specific motor skills that can be applied across the life course [10]. Meta-analyses of mainly cross-sectional studies have demonstrated that there is a significant correlation between motor competence and physical activity amongst children and adolescents [7, 8]. When considering longitudinal and experimental studies, however, the picture is less clear, likely due to their divergent methods of measurement and varied time periods of investigation [6].

Self-perception is another potentially important antecedent of physical activity participation amongst children and adolescents. Physical self-perception is a higher order construct, which some authors have postulated to include perceptions regarding physical strength, physical conditioning, body appearance and sport/athletic competence [11]. Other researchers also refer to perceptions of body appearance as well as the more general construct of perceived physical ability [12, 13]. The concept of physical competence has also been proposed by Harter [14], whereas other concepts such as perceived adequacy proposed by Hay [15] are conceptually close to perceived physical competence. More recently, the concept of perceived motor skills has been highlighted [16] and is described as a subset of perceived sport and athletic competence, specifically referring to an individual’s perception of their motor skills, such as running, jumping, throwing, catching and striking [17]. Therefore, perceived motor competence reflects an individual’s notions of their degree of mastery of motor skills. Despite the nuances in the self-related concepts they specify, perceived physical ability/competence, perceived sport/athletic competence and perceived motor competence all reflect perceptions of physical competence relevant to the domain of physical activity. Two meta-analyses have demonstrated that the broader concept of physical self-perception, which includes perceived physical competence (and its derivatives), are significantly related to physical activity in children and adolescents [8, 18].

In 2008, Stodden and colleagues proposed an influential model including the hypothesis that, in addition to physical fitness, the association between actual motor competence and physical activity is mediated by perceived motor competence [9]. They argue that better motor skills result in higher perceived motor competence, and ultimately more regular engagement in physical activity. The association between physical self-perception (including perceived motor competence) and actual motor competence was first investigated by De Meester and colleagues [19], who demonstrated positive associations between perceived and actual overall motor competence (*r* = 0.25), locomotor skills (*r* = 0.19), object control skills (*r* = 0.22) and stability/balance (*r* = 0.21).

Although evidence of each of the independent associations described in Stodden and colleagues’ [9] theoretical model have been systematically reviewed and quantitatively synthesised, there have been fewer attempts to synthesise the indirect mediated association proposed by the model. In a narrative review, Robinson and colleagues [20] noted preliminary evidence that perceived physical competence does mediate the association between actual motor competence and physical activity. In an update of the Robinson and colleagues [20] review, Barnett and colleagues [6] included studies that defined the construct as perceived motor competence, perceived sport competence, physical self-perception or physical self-confidence (and not self-esteem, self-efficacy, self-concept, global self-worth) and noted an insufficient number of studies examining the mediating effect of perceived physical competence on the association between actual motor competence and physical activity, preventing a meta-analysis from being conducted. Therefore, the aim of this study was to use structural equation modelling meta-analysis to examine the mediating effect of perceived physical competence on the association between actual motor competence and physical activity. Unlike traditional univariate meta-analysis, structural equation modelling can model complex multivariate relationships and is therefore particularly useful for theory-driven analysis of indirect associations, such as the association proposed between actual motor competence, perceived motor competence and physical activity [21]. Consistent with previous reviews on this topic [6, 19], the current study used the broader concept of perceived physical competence (including the concepts of perceived motor competence, perceived athletic/sport competence, perceived physical ability) to quantitatively synthesise the mediating effect of physical self-perception on the association between actual motor competence and physical activity. Additionally, we aimed to determine whether the pooled mediation effect in the included studies differed on the basis of average participant age. This analysis was included on the basis of the tenets of Stodden and colleagues' model, which postulated that the association between perceived physical competence with actual motor skills and physical activity will be stronger in older children and adolescents compared with young children [9].

## Methods

This systematic review and meta-analysis was preregistered in the International Prospective Register For Systematic Reviews database (CRD42024499080) and was conducted in accordance with the Preferred Reporting Items for Systematic Reviews and Meta-Analyses (PRISMA) guidelines [22].

### Identification of Studies and Search Strategy

Searches were conducted across five databases: Medline (via Ovid), SPORTDiscus, PsycINFO, Cumulative Index to Nursing and Allied Health Literature (CINAHL) and Education Resources Information Center (ERIC) for observational empirical research, written in English from inception to December 2023. The search strategy contained keywords from four categories: actual motor development (e.g. motor skill*, movement skill*, gross motor), perceived physical competence (e.g. perceived motor competenc*, perceived sport competenc*, perceived athletic competenc*), physical activity (e.g. physical activit*, exercise*, sport*) and children/adolescents (e.g. child*, youth*, juvenil*). A full list of keywords can be found in Appendix A.

### Inclusion and Exclusion Criteria

Studies were included if they met the following criteria: (1) participants were between 4–18 years of age (either at baseline or follow-up for longitudinal studies); (2) they included and reported on a sample of typically developing children; (3) physical activity was assessed by an objective measure (e.g. accelerometer, pedometer) or validated self/proxy-reported questionnaire (i.e. it used an assessment with previously reported validity or provided evidence of validity in the study); and (4) perceived physical competence (i.e. perceived physical ability/competence, perceived sport/athletic competence or perceived motor competence) was assessed using participant self-report (i.e. not a proxy, for example parent or guardian). Studies which only assessed other aspects of physical self-perception including perceived physical strength, perceived physical conditioning and perceived body appearance were excluded; (5) actual motor competence was assessed using a process (i.e. quality of movements assessed by an assessor) or product (i.e. the objective outcome from the execution of a skill such as distance jumped) oriented assessment; (6) the study assessed any of the following: locomotor skills, object control skills or gross motor skills (i.e. a combination of locomotor and object control skills). Studies which included a combination of gross and fine motor skills in the assessment of gross motor skills were included; (7) a bivariate or partial correlation was reported (or could be calculated based on reported results of a regression analysis, for example, from the *t*-test or standardised regression coefficient) for at least one of the proposed relationships (e.g. between perceived physical competence and physical activity); and (8) the study used a cross-sectional or longitudinal design.

### Selection and Screening

Title and abstract screening was conducted using ASReview, an open source software package which expedites the review process through the use of a machine learning algorithm and active learning that orders titles and abstracts on the basis of their relevance [23]. To determine the prevalence of relevant articles in the dataset and ultimately the optimal stopping point of the machine-learning-assisted review, a random sample of 200 titles and abstracts were initially screened. On the basis of these results, an upper limit of potentially relevant articles (*R*) was calculated as:

$$R = \text{N}\left(\text{p}\right)+1.96 \times \sqrt{\frac{(\text{p}*(1-\text{p})}{n}}$$

where *N* is the total number of articles identified in the literature search, *p* is the proportion of relevant articles identified in the random sample of articles and *n* is the number of random titles and abstracts screened. To ensure no potentially relevant studies were missed, it was also a requirement that 50 consecutive titles and abstracts were labelled as irrelevant.

The aforementioned approach to screening resulted in 1604 titles and abstracts being screened. Title and abstract screening were completed by a single reviewer (M.B.) in ASReview. A second reviewer (H.F.W.W.) screened the full list of titles and abstracts considered to be irrelevant by the first reviewer. All titles and abstracts labelled as relevant by either the first or second reviewer were retrieved for full-text screening. Full texts were screened by a single reviewer (M.B.) in Covidence (Veritas Health Innovation, Melbourne, Australia) and rescreened by a second reviewer (K.F., H.W., H.F.W.W.) to ensure that all studies that met the inclusion criteria were included in the review. Where there was a disagreement between the reviewers, the lead author reread the article and made the final decision.

### Data Extraction

Data were extracted from relevant papers by three independent reviewers (M.B., K.F., H.W.). Data for each paper were extracted by a single reviewer and checked for accuracy by a second independent reviewer. The extracted data included year of publication, the name of the study or cohort, the country in which the study was conducted in, sample size, average age of participants, gender/sex (% girl/female), assessment tools used to measure actual motor competence, perceived physical competence and physical activity and the correlations between each of the study variables. When studies reported on multiple correlations between actual motor competence and perceived physical competence, the correlation between assessments with the greatest level of alignment was extracted (e.g. correlation between actual locomotion skills and perceived locomotion skills [17, 19]). If studies reported on multiple physical activity outcomes, the correlation with the outcome which most closely resembled moderate-to-vigorous intensity physical activity was extracted. This decision was made as the strongest evidence regarding the health benefits of physical activity are for moderate-to-vigorous intensity physical activity. Overall physical activity was included if it was the only physical activity outcome reported. When studies reported on multiple equally relevant correlations, the median correlation of all equally relevant correlation coefficients was extracted (e.g. reporting on the correlation between throwing, catching, etc. with physical activity separately, rather than reporting on the correlation with a summation of multiple object control skills; and/or reporting the association between perceived physical competence and moderate intensity physical activity and vigorous intensity physical activity separately). If there was an even number of correlation coefficients, the more conservative (i.e. closer to zero) correlation of the two correlations around the median was extracted. If a correlation coefficient was calculated from results reported in a regression analysis, the coefficient based on the model with the fewest covariates was extracted. If a study reported on correlations for subgroups separately (e.g. reporting the correlation in boys/male and girls/female separately), these correlations were recorded separately and treated as independent.

### Quality Appraisal Criteria

The quality appraisal criteria (Appendix C) were adapted from the Strengthening the Reporting of Observation Studies in Epidemiology (STROBE) statement [24] and the Consolidated Standards of Reporting Trials (CONSORT) 2010 statement [25]. Six criteria were generated that aligned with previous research [19]. The criteria were scored with either a ‘Y’ (met the criteria), ‘N’ (did not meet the criteria), ‘UC’ (unclear if they had met the criteria) or ‘NA’ (not applicable). Quality appraisal was completed independently, with each article reviewed by a single reviewer only (H.W., M.B., K.F.). To ensure consistency in the review process, the three reviewers initially appraised five studies independently and discussed their findings. Differences were discussed and resolved. The remaining studies were equally divided amongst the researchers. An overall quality score was calculated as the proportion of relevant quality criteria a study met [19].

### Data Synthesis and Analysis

**Fig. 1:**
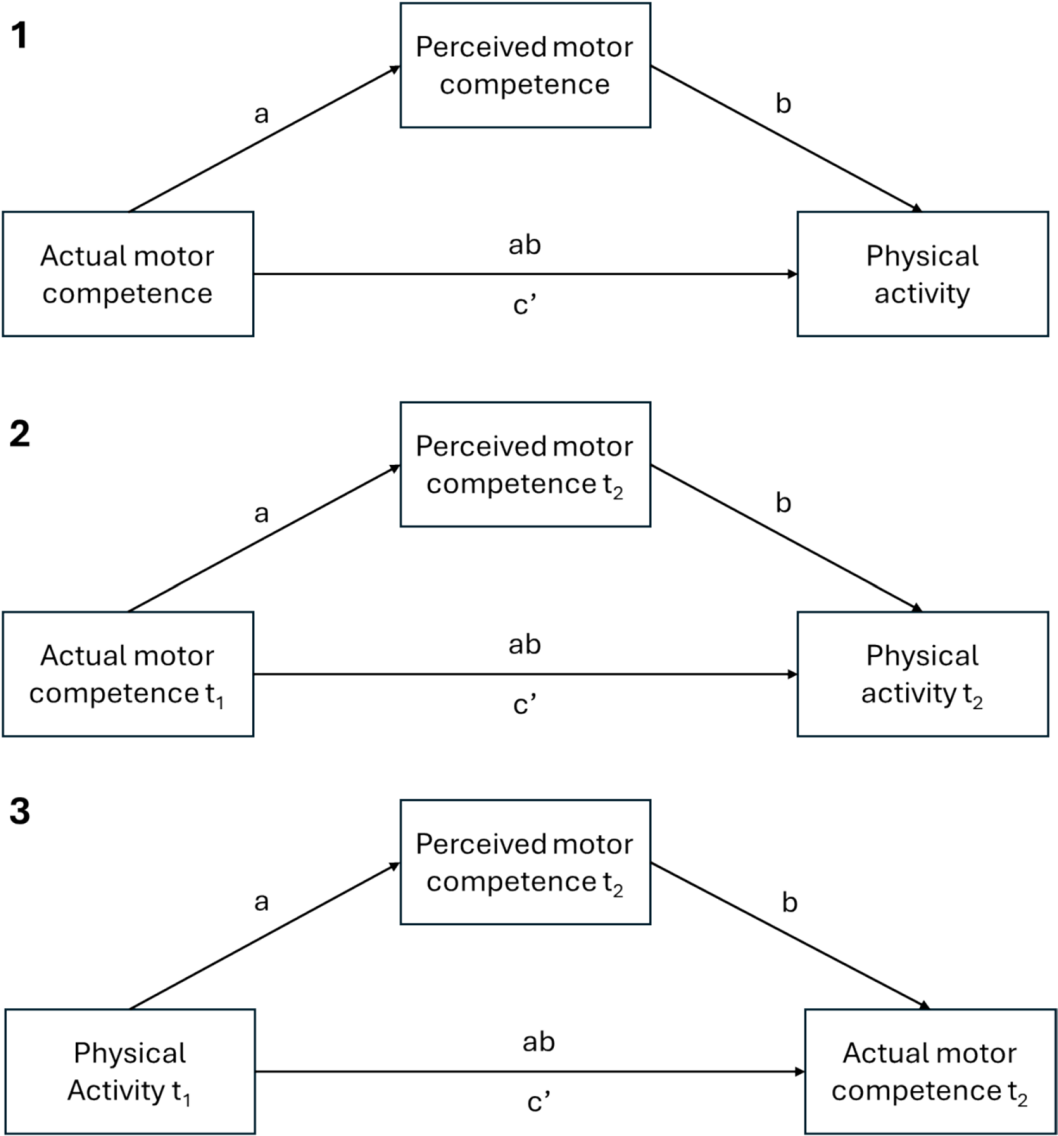
Estimated (1) concurrent mediation model and (2,3) lagged mediation models. *t*_*1*_ baseline assessment, *t*_*2*_ follow-up assessment, *a* correlation between independent variable and mediation, *b* partial correlation between mediator variable and outcome controlling for independent variable, *c’* partial correlation between the independent variable and outcome controlling for the mediator variable, *ab* the indirect association between the independent variable and outcome variable through the mediation variable (i.e. $a \times b$)

A pooled mediation model was estimated using random-effects one-stage meta-analytic structural equation modelling (OSMA-SEM) [26] using the metaSEM package [27] in R. OSMA-SEM treats studies as ‘subjects’ and correlation-matrices as ‘variables’ in the analysis and models the pooled correlation coefficients as a function of the structural equation model (SEM) parameters (see Fig. 1 for the mediation models tested via OSMA-SEM). The effect size of the correlations in the SEM model was interpreted as small (*r* = 0.10), moderate (*r* = 0.30) or large (*r* = 0.50). The relative size of the indirect effect was calculated as the ratio (presented as a percentage) of the indirect effect to the total effect (i.e.$ab :ab+c{\prime}$). Between study heterogeneity in direct effects was expressed as *I*^2^ values. All analyses were conducted separately for gross motor skills, locomotor skills and object control skills.

OSMA-SEM uses full-information maximum-likelihood (FIML) to handle missing data efficiently, under the assumption that the data are missing at random [26]. If correlation coefficients were missing due to not being examined in a study (e.g. a study examined the relation between actual motor competence and perceived physical competence but not physical activity), they were assumed to be missing at random. When a correlation was missing because a study only reported significant correlations (*p* < 0.05), it was considered to violate the assumption that the data were missing at random [26], and these studies were excluded.

For studies which examined concurrent associations (i.e. cross-sectional studies, concurrent change), the SEM was estimated with actual motor competence as the independent variable, perceived physical competence as the mediator and physical activity as the outcome (Fig. 1a). For lagged-longitudinal studies, two separate models were estimated. One model was estimated with actual motor competence at baseline as the independent variable and perceived physical competence and physical activity at follow-up as the mediator and outcome, respectively (Fig. 1b). Another model was estimated with physical activity at baseline as the independent variable and perceived physical competence and actual motor competence at follow-up as the mediator and dependent variable, respectively (Fig. 1c).

To determine whether any of the direct effects estimated in the model varied on the basis of participant age, moderation analysis with age as a continuous variable was conducted using the webMASEM Shiny app [28]. Where age was a significant moderator based on an omnibus test, separate OSMA-SEM models were estimated for three different age groups: early childhood (4 years), middle childhood (5–9 years) and adolescence (10–18 years), to determine whether the mediation effect significantly differed on the basis of age. Additionally, sensitivity analysis was conducted to determine whether the results of the meta-analysis differed between studies which used process versus product assessments of actual motor competence; between studies which assessed perceived motor competence versus studies which assessed other forms of physical self-perception (i.e. assessed perceived physical ability/competence, perceived sport/athletic competence); and between studies which assessed physical activity using wearable devices (e.g. accelerometers, pedometers) and studies which assessed physical activity using self/parent-reported questionnaires. Given the relatively few studies which assessed lagged associations, moderation and sensitivity analysis were only conducted for studies reporting on concurrent associations.

## Results

### Study Selection

**Fig. 2:**
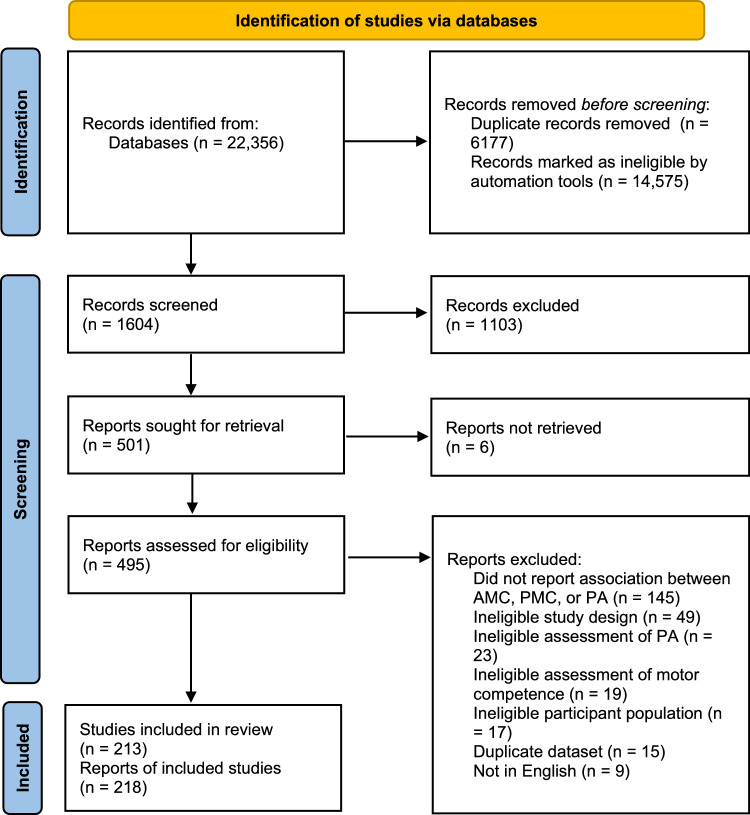
PRISMA flowchart. *AMC* actual motor competence, *PMC* perceived motor competence, *PA* physical activity

After removing duplicate records, the search identified 16,179 potentially relevant articles. Of these, 1604 were screened manually, and the remaining 14,575 titles and abstracts were rated below the threshold on the basis of the machine learning algorithm to warrant manual screening. Amongst the titles and abstracts screened, 1103 were deemed to be irrelevant, and 501 full texts were sought for screening. Six full texts could not be accessed, so 495 full texts were screened for inclusion. Of the full texts screened, 277 were deemed ineligible (see Fig. 2 for reasons), whilst 218 reports that reported on 213 studies met the inclusion criteria.

### Study Characteristics

A list of included studies and an overview of study characteristics are available in Appendix B.

Studies were conducted in Europe (*k* = 98), North America (*k* = 56), Australia (*k* = 25), Asia (*k* = 14), South America (*k* = 14) and Africa (*k* = 3). Three studies were conducted across multiple continents. The average age of participants was 4 years to 17.7 years (median: 8.5 years). The percentage of samples that were female ranged from 25 to 100% (median: 50%).

Actual motor competence was assessed in 180 studies, perceived physical competence in 122 studies and physical activity in 151 studies. The most commonly utilised assessments for actual motor competence were a version of the test of gross motor development (TGMD; *k* = 81), the Körperkoordinationstest für Kinder (KTK; *k* = 18), a version of the Movement Assessment Battery (MABC; *k* = 11) and a version of the Bruininks-Oseretsky Test of Motor Proficiency (BOT; *k* = 11). The majority of studies/articles used process-oriented assessments of actual motor competence (*k* = 111, 59%). Perceived physical competence was most commonly assessed using the pictorial scale of Perceived Movement Skill Competence (*k* = 26), the Physical Self-Perception Profile—Sports Competence subscale (*k* = 16) and the Pictorial Scale of Perceived Competence—Physical Competence subscale (*k* = 13), the Self-Perception Profile for Children—Athletic Competence subscale (*k* = 10) and the Perceived Competence Scale for Children—Physical Competence subscale (*k* = 8). Physical activity was most commonly assessed using devices including accelerometers (*k* = 88) and pedometers (*k* = 22). The most used questionnaire for assessing physical activity was the Physical Activity Questionnaire for Older Children or Adolescents (*k* = 15).

### Quality Appraisal

All applicable quality criteria were met in 13 articles; 80–99% of applicable criteria met in 21 articles; 60–79% met in 74 articles; 40–59% met in 24 articles; and < 40% met in 4 articles. There were 82 articles that did not provide sufficient details to accurately determine the degree to which they met at least one applicable criterion. The most common criteria that were not met or adequately described were recruiting a representative sample (81.7% of studies), reporting an acceptable inter-rater reliability for the assessment of AMC (62.1% of studies that used a process-oriented assessment of AMC) and reporting an adequate rate of participant attrition (40.8% of studies). Detailed quality appraisal results for individual articles are available in Appendix C.

### Meta-Analysis

#### Concurrent Associations

Results from the SEM meta-analyses of the concurrent association between actual motor competence (i.e. gross motor competence, locomotion and object control) with perceived physical competence and physical activity are presented in Table 1. Results for gross motor competence (ab = 0.034), locomotion (ab = 0.029) and object control (ab = 0.030) demonstrated that there was only a weak absolute indirect effect from actual motor competence through perceived physical competence on physical activity. Additionally, the relative magnitude of the indirect effect was small, with perceived competence mediating between 16.7% and 20.6% of the association between actual competence and physical activity.

**Table 1:** Results from the one stage meta-analysis structural equation modelling examining concurrent mediation models

	*k* samples	*n* participants	a	b	c'	ab	ab:ab + c’
Gross motor competence	179	56,681	0.215 (0.175, 0.254) *I*^2^ = 85.3%	0.160 (0.124, 0.196) *I*^2^ = 88.5%	0.172 (0.143, 0.201) *I*^2^ = 76.2%	0.034 (0.026, 0.044)	16.7%
Locomotion	204	65,136	0.165 (0.112, 0.218) *I*^2^ = 91.8%	0.178 (0.142, 0.214) *I*^2^ = 88.5%	0.113 (0.073, 0.153) *I*^2^ = 90.2%	0.029 (0.020, 0.041)	20.6%
Object control	201	59,102	0.173 (0.125, 0.220) *I*^2^ = 88.4%	.172 (0.138, 0.206) *I*^2^ = 87.3%	0.143 (0.108, 0.177) *I*^2^ = 85.0%	0.030 (0.021, 0.040)	17.2%

Results presented as *r* (95% CI)

##### Sensitivity Analysis

The results from the sensitivity analysis exploring differences in model results on the basis of methodological differences between included studies is displayed in Appendix D. Although there were some differences observed in individual paths in the model, for the most part, the indirect effect between actual motor competence with physical activity through perceived motor competence was robust to methodological differences between studies. There were significant differences in indirect effects between studies which assessed the association between gross motor competence and physical activity when assessed using wearable devices compared with self- or parent-reported questionnaires (*z* = 2.50, *p* = 0.012), and between studies which assessed the association between locomotion skills and physical activity which assessed actual locomotor competence using process- and product-oriented assessment (*z* = 2.76, *p* = 0.006).

#### Lagged Associations

Results from the SEM meta-analyses of lagged association between actual competence (gross motor competence, locomotion and object control) with perceived physical competence and physical activity are presented in Table 2. Considering gross motor competence, perceived competence did not significantly mediate the association between actual competence and physical activity in either direction.

**Table 2:** Results from the one stage meta-analysis structural equation modelling examining lagged mediation models

	*k* samples	*n* participants	a	b	c'	ab	ab:ab + c’
*Gross motor competence*							
AMC1 → PMC2 → PA2	10	2938	0.236 (0.091, 0.353) *I*^2^ = 88.2%	0.135 (− 0.070, 0.320) *I*^2^ = 89.5%	0.143 (0.062, 0.222) *I*^2^ = 36.0%	0.032 (− 0.003, 0.032)	18.2%
PA1 → PMC2 → AMC2	11	2562	0.120 (− 0.019, 0.255) *I*^2^ = 79.6%	0.070 (− 0.138, 0.266) *I*^2^ = 85.0%	0.076 (0.023, 0.127) *I*^2^ = 10.7%	0.008 (− 0.020, 0.042)	9.9%
*Locomotion*							
AMC1 → PMC2 → PA2	13	3398	0.205 (0.119, 0.295) *I*^2^ = 46.0%	0.215 (0.005, 0.364) *I*^2^ = 69.5%	0.027 (− 0.065, 0.114) *I*^2^ = 71.5%	0.044 (0.001, 0.085)	62.3%
PA1 → PMC2 → AMC2	14	3517	0.197 (0.148, 0.248) *I*^2^ = 0.0%	0.164 (− 0.015, 0.327) *I*^2^ = 82.7%	0.149 (0.078, 0.227) *I*^2^ = 55.9%	0.032 (− 0.003, 0.067)	17.8%
*Object control*							
AMC1 → PMC2 → PA2	10	1943	0.234 (0.097, 0.374) *I*^2^ = 70.1%	0.224 (0.088, 0.323) *I*^2^ = 50.2%	0.105 (0.061, 0.136) *I*^2^ = 32.1%	0.052 (0.008, 0.108)	33.2%
PA1 → PMC2 → AMC2	10	1367	0.183 (0.113, 0.254) *I*^2^ = 7.7%	0.182 (0.068, 0.295) *I*^2^ = 0.0%	0.094 (0.022, 0.165) *I*^2^ = 0.0%	0.033 (0.012, 0.062)	26.3%

Results presented as *r* (95% CI)

*AMC* actual motor competence, *PMC* perceived motor competence, *PA* physical activity

For locomotion skills, there was a significant indirect effect between actual competence at baseline and physical activity at follow-up through perceived physical competence. Although the relative magnitude of this association was large—perceived physical competence mediated 62.3% of the association between actual locomotor competence and physical activity—the absolute indirect association was small (ab = 0.044). Perceived physical competence also mediated the association between physical activity at baseline and locomotion skills at follow-up, but the absolute (ab = 0.032) and relative (17.8%) strength of the association were also small. Similar results were exhibited for object control skills, where actual motor competence at baseline had a small but significant indirect effect with physical activity at follow-up through perceived physical competence (ab = 0.052). There was also a small indirect effect between physical activity at baseline and actual object control competence at follow-up mediated by perceived physical competence (ab = 0.033).

### Moderation by Participant Age

The results from the moderation analysis are presented in Table 3. The results demonstrated that age significantly moderated the results for the gross motor and locomotion models. Results from a subgroup analysis (Table 4) demonstrated that perceived physical competence had a significantly stronger mediation effect on the relationship between gross motor competence and physical activity (*z* = 3.06, *p* = 0.002) and on actual locomotor competence and physical activity (*z* = 2.41, *p* = 0.016) in adolescents compared with middle childhood.

**Table 3:** Results from moderation analysis examining differences in estimated paths on the basis of average participant age

	Omnibus test	a	b	c'
Gross motor competence	*χ*^2^(3) = 13.32, *p* = 0.004	0.061 (− 0.001, 0.102)	0.031 (− 0.008, 0.069)	0.012 (− 0.018, 0.042)
Locomotion	*χ*^2^(3) = 9.19, *p* = 0.027	0.024 (− 0.032, 0.081)	0.050 (0.015, 0.085)	− 0.012 (− 0.052, 0.030)
Object control	*χ*^2^(3) = 5.97, *p* = 0.113	0.029 (− 0.023, 0.081)	0.032 (0.000, 0.068)	− 0.007 (− 0.043, 0.029)

Results presented as *r* (95% CI)

**Table 4:** Results from the one stage meta-analysis structural equation modelling examining concurrent mediation models stratified by average participant age

	*k* samples	*n* participants	a	b	c'	ab	ab:ab + c’
**Gross motor competence**							
Early childhood (4 years)	27	3389	0.234 (0.096, 0.363) *I*^2^ = 60.6%	0.100 (0.008, 0.229) *I*^2^ = 24.5%	0.155 (0.081, 0.229) *I*^2^ = 51.1%	0.023 (0.001, 0.059)	13.1%
Middle childhood (5–9 years)	74	17,891	0.155 (0.096, 0.212) *I*^2^ = 83.9%	0.137 (0.082, 0.192) *I*^2^ = 86.3%	0.161 (0.122, 0.198) *I*^2^ = 57.9%	0.021 (0.012, 0.034)	11.7%
Adolescence (10–18 years)	75	35,223	0.317 (0.275, 0.356) *I*^2^ = 63.8%	0.172 (0.117, 0.226) *I*^2^ = 90.5%	.190 (.137, .240) *I*^2^ = 84.2%	0.054 (0.037, 0.073)	22.3%
**Locomotion**							
Early childhood (4 years)	30	5243	0.188 (0.078, 0.301) *I*^2^ = 70.8%	0.131 (0.042, 0.220) *I*^2^ = 0.0%	0.137 (0.044, 0.228) *I*^2^ = 79.8%	0.025 (0.008, 0.050)	15.2%
Middle childhood (5–9 years)	101	28,889	0.118 (0.049, 0.187) I^2^ = 88.8%	0.133 0.079, 0.187) *I*^2^ = 87.9%	0.116 (0.055, 0.177) *I*^2^ = 91.7%	0.016 (0.007, 0.28)	11.9%
Adolescence (10–18 years)	73	31,004	.224 (.117, .331) *I*^2^ = 95.4%	.223 (.173, .272) *I*^2^ = 89.3%	0.105 (0.046, 0.159) *I*^2^ = 82.6	0.050 (0.026, 0.077)	32.2%

Results presented as *r* (95% CI)

## Discussion

The aim of this systematic review and meta-analysis was to examine the extent to which perceived physical competence mediated the association between actual motor competence and physical activity in children and adolescents. This is the first study to quantitatively synthesise the existing literature on this mediation effect. A small absolute and relative indirect effect from actual motor competence to physical activity through perceived physical competence was evident. The strength of the indirect association was consistently small for gross motor competence, locomotion and object control skills; and between studies examining the concurrent and lagged indirect association between actual motor competence and physical activity through perceived physical competence. The results of the sensitivity analysis also demonstrated that the indirect effect was largely robust to methodological differences amongst studies, including the assessment of actual motor skills, perceived motor skills and physical activity. Importantly, mediation is based on the assumption of a causal process which occurs over time [29]. Because cross-sectional data are collected at a single timepoint, it is not possible to determine temporal precedence of the variables being modelled. The consistency in results across studies examining the concurrent and lagged associations observed in the current study allay concerns related to these noted limitations of cross-sectional mediation of longitudinal processes [29]. Therefore, similar to a previous systematic review on this topic (without a meta-analysis) [6], the results from the current study do not provide strong evidence to support the hypothesised association between actual motor competence with physical activity mediated through perceived physical competence [9]. However, even a small mediational process may have some practical relevance in public health and developmental contexts, especially when accumulated at a population level.

The second aim of this study was to examine the impact of age on the relationship between actual motor competence and physical activity mediated by perceived physical competence. The relationship was stronger in adolescents (aged 10–18 years) compared with children (< 10 years), providing support for the moderating impacts of age and the model proposed by Stodden and colleagues [9]. This is consistent with theories of self-perception which postulate that younger children have limited self-perception skills and conflate perceptions about their desired self with their actual self [30]. The accuracy of self-perception improves with age and some researchers have postulated that children are not likely to be capable of accurately perceiving their own capabilities until the age of 9 years [31]. Our results complement those of a meta-analysis conducted by Babic and colleagues [18] and a pooled analysis conducted by Barnett and colleagues [32], who demonstrated a stronger association between physical self-perceptions and physical activity, and a stronger association between actual motor skills and physical activity, respectively, with increasing age. Conversely, our results conflict with those of a meta-analysis conducted by De Meester and colleagues [19], who examined the relationship between actual and perceived physical competence and did not find a moderating impact of age. However, the review by De Meester and colleagues [19] was limited in terms of the numbers of older samples, whilst the review by Babic and colleagues [18] was also limited by the number of younger samples included in their review. Therefore, the results of the current review may provide the most robust evidence of how age moderates the associations between actual motor competence, perceived physical competence and physical activity.

The results from this meta-analysis add to the results from previous analyses which have demonstrated the independent association between actual motor competence and perceived physical competence [8, 19]; actual motor competence and physical activity [7, 8]; and physical self-perception and physical activity [18]. Identification of the largely independent associations of actual and perceived motor competence with physical activity has important implications for the design of interventions or programs aiming to improve the physical activity levels of children and adolescents. Interventional studies targeting actual motor skills have demonstrated efficacy in improving the physical activity levels of children [33–35], and therefore actual motor competence is an obvious target for interventions aiming to increase physical activity. However, evidence of the effectiveness of targeting improvements in perceived motor skills simultaneously with actual motor skills on physical activity in children is limited [36]. There is some evidence to show that interventions can simultaneously improve actual and perceived motor competence [37, 38]. Conversely, some evidence demonstrates that interventions that improve actual motor skills decrease perceived physical competence in children [39]. More interventional research is therefore necessary in this area to identify the most effective methods of improving the physical activity levels of children and adolescents through targeting actual and/or perceived motor competence. A strategy to improve perceived physical competence which warrants consideration is the provision of individualised feedback highlighting areas of improvement so that a child’s perceived physical competence improves in unison with their actual motor competence [40]. Providing individualised feedback as part of a motor development intervention may reduce social comparison biases in children’s perceptions of their motor competence and simultaneously improve their actual and perceived motor competence skills [41].

Several person-centred studies, which group homogeneous subgroups of participants on the basis of their actual motor competence and their perceived motor competence, have found that approximately one-quarter to one-half of children and adolescents have perceptions about their motor competence which do not reflect their actual motor competence, also known as ‘divergent’ profiles [42–46]. For example, de Witte and colleagues [44] found that 32% of the children they assessed had below average actual motor skills and above average perceived motor skills, whilst 22% of children had above average motor skills but below average perceived motor skills. This may explain the modest correlations observed in the current meta-analysis, as there is a strong positive correlation between actual motor skills and perceived physical competence in some children and a strong negative correlation in others. This might also explain the moderating effects of age identified in the present study; older children may have less divergence between their actual and perceived motor competence, so the correlations between each of the variables will be stronger. Results from one person-centred analysis have also demonstrated that children who are high in actual and perceived motor competence engage in significantly more physical activity than children with high perceived motor competence and average actual motor competence [43]. In another person-centred analysis, researchers reported that children with low perceived motor competence and low actual motor competence engage in significantly less physical activity than children with low actual competence but average perceived motor competence [42]. Together with the results of the current meta-analysis, the results from person-centred analyses demonstrate that perceived physical competence may act more as a moderator, which augments the association between actual motor competence and physical activity rather than a mediator, as is currently hypothesised. That is, actual motor competence may only have a strong positive association with physical activity in children who also have a positive perception about their perceived physical competence, and vice versa. This is a hypothesis which is yet to be tested and warrants attention.

Considering directionality, synthesis of the included longitudinal studies demonstrated that the relationship between actual motor competence and physical activity, mediated by perceived physical competence, is bidirectional for locomotion and object control skills. That is, generally, children with better actual motor skills have better perceived physical competence and will therefore engage in higher levels of physical activity. Additionally, children who are more physically active develop more positive perceptions about their motor skills and as a result, are more motivated to participate in physical activities that demand high motor competence, which improves their actual motor competence [9]. However, some exceptions were identified; for example, perceived physical competence did not mediate the association between physical activity and gross motor competence when physical activity was an antecedent. Perceived physical competence also had a significantly greater mediating effect in the model examining the relationship between actual locomotion skills as an antecedent and physical activity as an outcome. This may be attributed to locomotion skills at baseline having only a trivial correlation with physical activity at follow-up, which is consistent with a previous review of longitudinal studies [6]. The bidirectional nature of the relationship may also be dependent on age. Stodden and colleagues [9] proposed that the relationship is not bidirectional in young children; whereby in young children physical activity is an antecedent of actual motor competence. Given the small number of longitudinal studies identified in this review, it was not possible to determine whether age moderated the results from the estimated lagged models. The effect of age on the lagged associations between physical activity and motor competence warrants further investigation.

Further research is also warranted with respect to the aspects identified from the sensitivity analysis. Although the results were for the most part robust to methodological differences between studies, there was some evidence that the indirect effect observed between studies differed depending on whether studies assessed physical activity using wearable devices or self/parent-reported questionnaires. Wearable devices and questionnaires assess different aspects of physical activity [47]. Wearable devices do not typically capture nuances in different types of physical activity such as cycling, swimming and climbing, and yet these behaviours rely on motor competence, whilst questionnaires typically do not assess incidental physical activity which occurs during activities of daily living; thus future research could consider the implications of their physical activity measurement. A difference was also observed for the association between locomotion skills and physical activity between studies which used process- and product-oriented assessment; this finding is also reflected in the literature [48].

This systematic review and meta-analysis had several strengths. The use of multivariate meta-analysis in the form of an OSMA-SEM meant that missing data could be handled using FIML. This facilitated the inclusion of a highly comprehensive collection of published studies which reported on at least one of the associations in the hypothesised model and meant that each of the meta-analyses on the concurrent associations was based on more than 180 studies and 50,000 individual participants, making this the most comprehensive review on the topic to date. Additionally, the use of structural equation modelling meta-analysis allowed for the empirical examination of hypothesised indirect effect, something that cannot be achieved using traditional meta-analytic techniques. There are also several limitations that need to be considered. First, most of the included articles were of modest methodological quality based on the quality appraisal tool used in the current study. Therefore, there may have been various forms of bias influencing the reported results across the included studies. Another limitation is that the review was restricted to articles written in English, potentially introducing a language bias. This is evidenced by greater than 80% of the studies included in the review being conducted in Europe, North America or Australia. Significantly different patterns of physical activity participation [49, 50] and motor skill competence [51] have been observed across different cultures and regions of the world. Cultural differences may also exist in the practise of humility, self-effacement or modesty, which may be higher in Eastern cultures [52] and impact on a child’s perceptions of their motor competency in comparison with their actual motor competency. Further, there is publication bias towards countries such as North America (termed as part of the minority world) where most of the world’s children do not reside [53]. Third, the meta-analysis assumed a linear association between each of the variables in the model. A recent cross-sectional pooled analysis determined that there was a non-linear association between motor skill competence and physical activity with observed associations stronger when children were at the higher end of the skill spectrum [32]. Therefore, important dose–response relationships between the variables included in the model may have been missed. Finally, not all stages of the review process were independently completed by two reviewers, such as the quality appraisal of included studies, which may have reduced the methodological rigour of some aspects of the review.

## Conclusions

This systematic review and meta-analysis provides comprehensive evidence that perceived physical competence has only a small mediating effect on the association between actual motor competence and physical activity. Age moderated the association between actual gross motor competence and perceived physical competence, and the indirect effect of actual gross motor competence on physical activity through perceived physical competence was significantly stronger in adolescents compared with children. Results from lagged models also provide initial support that the small mediation effect of perceived physical competence on the association between actual motor competence and physical activity may be bidirectional, although additional longitudinal studies are necessary to further elucidate the bidirectional nature of this relationship.

## Supplementary Information

Below is the link to the electronic supplementary material.

Supplementary file1 (DOCX 16 KB)

Supplementary file2 (DOCX 226 KB)

Supplementary file3 (DOCX 170 KB)

Supplementary file4 (DOCX 23 KB)

## Declarations

### Data availability

The dataset and code used to run the primary analysis have been made publicly available on the OSF and can be accessed at https://osf.io/y2ncs/. Moderation analysis was conducted using the webMASEM Shiny app (https://sjak.shinyapps.io/webMASEM/), which is codeless; thus code for the moderation analysis is not available.
