# Supplementary material for: Children's and Adolescents’ Actual Motor Competence, Perceived Physical Competence and Physical Activity: A Structural Equation Modelling Meta-Analysis
Source: Sports Med. 2025 May 6;55(8):1923–36. doi: 10.1007/s40279-025-02233-2 (PMC12460483; doi:10.1007/s40279-025-02233-2)
Supplement: Supplementary file 1 — Supplementary file1 (DOCX 16 KB) [file 40279_2025_2233_MOESM1_ESM.docx]

Table A1 – Systematic literature search terms

| **Actual motor competence** | **AND** | **Perceived physical competence** | **AND** | **Youth** | **AND** | **Physical activity** |
| --- | --- | --- | --- | --- | --- | --- |
| motor skill* OR movement skill* OR motor development OR gross motor OR object control OR locomoto* skill* OR motor competen* OR basic movement* OR motor function OR fms |  | perceived motor competenc* OR perceived sport* competenc* OR perceived physical competenc* OR perceived athletic competenc* OR perceived athletic skill* OR perceived movement compenten* OR perceived fundamental motor OR perceive fundamental movement OR perceived movement skill* OR perceived motor skill* OR perceived skill competenc* OR perceived skill proficienc* OR  perception* of motor competenc* OR perception* of sport* competenc* OR perception* of physical competenc* OR perception* of athletic competenc* OR perception* of athletic skill* OR perception* of movement compenten* OR perception* of fundamental motor OR perception* of fundamental movement OR perception* of movement skill* OR perception* of motor skill* OR perception* of skill competenc* OR perception* of skill proficienc* OR perceived object control OR perceived loctomot* OR perceptions of object control OR perceptions of locomot* OR perceived physical competence OR perception* of physical competence |  | child* OR adolescen* OR student* OR teen* OR youth* OR pediatric OR paediatric OR pube* OR juvenil* OR school* OR youngster OR preschool OR pre-school OR kindergart* OR kid* OR kids* OR young people |  | physical* activit* OR exercis* OR energetic play OR active play OR energetic play OR movement behavio* OR sport* OR ltpa OR mvpa OR moderate-to-vigorous OR leisure time |
