# Supplementary material for: Children's and Adolescents’ Actual Motor Competence, Perceived Physical Competence and Physical Activity: A Structural Equation Modelling Meta-Analysis
Source: Sports Med. 2025 May 6;55(8):1923–36. doi: 10.1007/s40279-025-02233-2 (PMC12460483; doi:10.1007/s40279-025-02233-2)
Supplement: Supplementary file 2 — Supplementary file2 (DOCX 226 KB) [file 40279_2025_2233_MOESM2_ESM.docx]

Table B1- Characteristics of included studies

| Author (year) | Study name/ country | Participants | Assessment of AMC  (OC = Object control, Loco = Locomotor,) | Assessment of PMC  (OC = Object control, Loco = Locomotor, GM = Gross motor, Prox = Proxy) | Assessment of PA |
| --- | --- | --- | --- | --- | --- |
| Concurrent Association | | | | | |
| Aadland et al. (2017) | Norway | N = 697  Age = 10.2  Girls = 51.2% | Catching one handed, throwing at a wall target, shuttle run (GM) | N/A | Accelerometer (ActiGraph GT3X+) |
| Aalizadeh, Mohamadzadeh, & Hosseini (2014) | Iran | N = 241  Age = 8.5  Girls = nr | TGMD-2 (OC) | N/A | 7-day PA recall |
| Adank, Van Kann, Remmers, Kremers, & Vos (2021) | Netherland  Stimulating an Active Lifestyle Through Physical Education | N = 371  Age = 10.1  Girls = 52.3% | Athletic Skills Track (GM) | N/A | Accelerometer (ActiGraph GT3X+) |
| Afthentopoulou, Venetsanou, Zounhia, & Petrogiannis (2018) | Greece | N = 121  Age 7.6  Girls = 53.7% | BOT-2 SF (GM) | PSPC-PC (Prox) | Pedometer (Walking Style Pro HJ-720IT-E2 (HJ-720)) |
| Amraei & Azadian (2021) | Iran | n = 320  Age = 8-12  Girls = 100% | TGMD-3 (GM) | PSDQ (Prox) | N/A |
| Anderson, Mâsse, Zhang, Coleman, & Chang (2009) | USA | N = 432  Age = 8-15  Girls = 58.7% | N/A | AIQ-C (Prox) | PAQ-C |
| Bai, Chen, Vazou, Welk, & Schaben (2015) | USA | N = 1,552  Age = nr  Girls = nr | N/A | PCSC-PC (Prox) | Youth Activity Profile |
| Balaban (2018) | Czech Republic | N = 201  Age = 9.22  Girls = 43.3% | TGMD-2 (OC, Loco, GM) | N/A | Accelerometer (ActiGraph GT3X) |
| Bardid et al. (2016) | Netherlands | N = 161  Age = 8.82  Girls = 59.6% | KTK (Loco) | SPPC-AC (Prox) | N/A |
| Barnett, Morgan, Van Beurden, Ball, & Lubans (2011) | Australia  Move It Groove It | N = 215  Age = 16.4  Female = 51.6% | Get Skilled Get Active (OC, Loco) | PSPP – SC (Prox) | Adolescent Physical Activity Recall Questionnaire |
| Barnett, Ridgers, Hesketh, & Salmon (2017) | Australia  InFANT | Sample 1  N = 152  Age = 4.7  Girls = 50.7%  Sample 2  N = 78  Age = 6.6  Girls = 44.9% | N/A | PSPMSC (GM) | Accelerometer (ActiGraph GT1M) |
| Barnett, Ridgers, & Salmon (2015) | Australia | N= 100  Age = 6.3  Girls = 44% | TGMD-2 (OC) | PSPMSC (GM) | Accelerometer (ActiGraph GT3X) |
| Barnett et al. (2019) | Australia | N = 261  Age = 9-11  Females = 53.3% | TGMD-2 (OC, Loco) | PSPP – SC (Prox) | Accelerometer (ActiGraph GT3X) |
| Barnett, Hinkley, Okely, & Salmon (2013) | Australia  HAPPY | N = 76  Age = 4.1  Girls = 55% | TGMD-2 (OC, Loco) | N/A | Accelerometer (ActiGraph GT1M) |
| Bernal, Lhuisset, Bru, Fabre, & Bois (2024) | France | N = 324  Age = 8.58  Girls = 53.7% | Eurofit battery (Loco) | N/A | Accelerometer (ActiGraph GT3X+) |
| Bezerra et al. (2021) | Brazil  Movement’s Cool | N = 201  Age = 4.0  Girls = 51.4% | TGMD-2 (OC, Loco) | N/A | Accelerometer (ActiGraph GT3X) |
| Blomqvist, Mononen, Tolvanen, & Konttinen (2019) | Finland | N = 477  Age = 10-11  Girls = 50.3% | KTK, overhand throw (Loco, OC) | N/A | Accelerometer (ActiGraph) |
| Bois, Sarrazin, Brustad, Trouilloud, & Cury (2005) | France | n = 152  Age = 9.56  Girls = 55.3% | N/A | PCSC-PC (Prox) | Activity checklist |
| Bolger et al. (2019; Bolger et al. (2018) | Ireland  Project Spraoi | N = 76  Age = 6  Girls = 48.0% | TGMD-2 (OC, Loco, GM) | PSPMSC (OC, Loco, GM) | Accelerometer (ActiGraph GT3X) |
| Boucher, Doescher, & Sugawara (1993) | USA | N = 31  Age = 4.72  Girls = 48.4% | PDMS-2 (GM) | PCSC-PC (Prox) | N/A |
| Breau et al. (2021) | Germany  JolinchenKids—Fit and healthy in daycare | N = 193  Age = 4.2  Girls = 52.3% | Shuttle run, standing long jump, lateral jumping, one-leg stand, sit and reach (Loco) | N/A | Accelerometer (GENEActiv) |
| Bremer et al. (2020) | Canada | N = 110  Age = 7-14  Girls = 52% | PLAYfun tool (GM) | N/A | Pedometer (PiezoRX Steps Counter) |
| Brian et al. (2018) | Belgium and USA | N = 326  Age = 4-5  Girls = 50.0% | TGMD-2 (OC, Loco) | PSPMSC (OC, Loco) | N/A |
| Brian, Taunton, Shortt, Pennell, & Sacko (2019) | USA | N = 59  Age = 4.6  Girls = 52.6% | TGMD-2 (GM) | PSPMSC (GM) | Accelerometer (ActiGraph GT3X+) |
| Bürgi et al. (2011) | Switzerland  Ballabeina-Study | N = 217  Age = 5.2  Girls = 52% | Obstacle course, dynamic balance test (Loco) | N/A | Accelerometer (ActiGraph GT1M) |
| Burns et al. (2022) | USA | N = 440  Age = 8.9  Girls = 52% | TGMD-3 (GM) | N/A | Pedometer (Yamax DigiWalker CW600) |
| Burns & Fu (2018) | USA | N = 84  Age = 11.6  Girls = 47.6% | TGMD-3 (GM) | PCSC-PC (Prox) | Pedometer (Yamax DigiWalker CW600) |
| Burns, Brusseau, & Hannon (2017) | USA | N = 1460  Age = 8.4  Girls = 50.0% | TGMD-3 (OC, Loco, GM) | N/A | Accelerometer (ActiGraph wGT3X-BT) |
| Capio, Sit, Eguia, & Abernethy (2014) | Philippines | N = 32  Age = 6.54  Girls = 53.1% | TGMD-2 (OC, Loco, Tot) | N/A | Accelerometer (ActiGraph 7164) |
| Capio & Eguia (2021) | Hong Kong | N = 230  Age = 5.62  Girls = 52.6% | TGMD-2 (OC, Loco) | PSPC-PC (Prox) | Pedometer (NL-800) |
| Carballo-Fazanes, Díaz-Pereira, Fernández-Villarino, Abelairas-Gómez, & Rey (2023) | Spain | N = 57  Age = 4.62  Girls = 56.1% | Athletic Skills Track (Loco) | N/A | Activity tracker (Garmin Vivofit jr) |
| Carcamo-Oyarzun, Estevan, & Herrmann (2020) | Chile | N = 467  Age = 11.16  Girls = 43.9% | MOBAK (OC, Loco) | SEMOK (OC, Loco) | N/A |
| Carvalho et al. (2021) | Brazil | N = 148  Age = 8.7  Girls = 52.7% | TGMD-2 (GM) | N/A | Accelerometer (ActiGraph wGT3X-BT) |
| Chan, Ha, Ng, & Lubans (2019) | Hong Kong | N = 763  Age = 9.3  Girls = 62% | TGMD-2 (OC, Loco) | 12 item perceived movement skill scale (GM) | Accelerometer (ActiGraph GT3X+) |
| Chaves et al. (2015) | Portugal  Active Vouzela | N = 390  Age = 8.61  Girls = 52.3% | KTK (Loco) | N/A | Godin and Shephard questionnaire |
| Cliff, Okely, Smith, & McKeen (2009) | Australia  PANDA | N = 46  Age = 4.29  Girls = 45.7% | TGMD-2 (OC, Loco, GM) | N/A | Accelerometer (ActiGraph 7164) |
| Cohen, Morgan, Plotnikoff, Callister, & Lubans (2014) | Australia  SCORES | N = 460  Age = 8.5  Girls = 54% | TDGMD-2 (OC, Loco) | N/A | Accelerometer (ActiGraph GT3X+) |
| Coker & Herrick (2021) | USA | N = 117  Age = 12.60  Girls = nr | Functional Movement Screen (GM) | PSPP-SC (Prox) | N/A |
| Cook et al. (2019) | South Africa | N = 136  Age = 4.39  Female = 47% | TGMD‐2 (OC, Loco, GM) | N/A | Accelerometer (ActiGraph GT3X+) |
| Coppens et al. (2021) | Belgium | N = 206  Age = 10.83  Girls = 45.7% | KTK (Loco) | PhSCS (Prox) | FPAQ (organized sport) |
| Craft, Pfeiffer, & Pivarnik (2003) | USA | N = 46  Age = 12.0  Girls = 100% | N/A | SPPC-AC (Prox) | Heart Rate Monitor |
| Craike et al. (2014) | Australia | N = 732  Age = 13.6  Female = 100% | N/A | AIQ-C (Prox) | Active Australia physical activity questionnaire |
| Crane, Foley, Naylor, & Temple (2017) | Canada | N = 250  Age = 5.66  Girls = nr | TDGMD-2 (OC, Loco) | PSPC-PC (Prox) | N/A |
| Crane, Foley, & Temple (2023) | Canada | N =129  Age = 8.3  Girls = 48.1% | TDGMD-2 (OC, Loco) | PSPC-PC (Prox) | Accelerometer (Actigraph GT1M) |
| Crocker, Eklund, & Kowalski (2000) | Canada | N = 466  Age = 11.7  Girls = 52.8% | N/A | PSPP-SC (Prox) | PAQ-C |
| Cumming et al. (2011) | UK | N = 407  Age = 13.2  Females = 100% | N/A | PSPP-SC (Prox) | PAQ-A |
| Davison, Schmalz, & Downs (2010) | USA | N = 151  Age = 13.33  Girls = 100% | N/A | G-DAS-C (Prox) | Accelerometer (Actigraph 7164) |
| de Bruijn & van der Wilt (2023) | Netherlands | N = 182  Age = 9.90  Girls = 51.1% | N/A | PMC-C (GM) | Dutch National Assessment of PE Sport Participation Questionnaire |
| De Meester, Maes, et al. (2016) | Belgium | N = 215  Age = 13.64  Girls = 44.0% | KTK (Loco) | PSPP-SC (Prox) | FPAQ |
| De Meester, Stodden, et al. (2016) | USA | N = 361  Age = 9.50  Girls = 50.1% | TGMD-2 (GM) | SPPC-AC (Prox) | Accelerometer (ActiGraph GT3X+) |
| de Oliveira Martins, Flôres, Valentini, & Copetti (2023) | Brazil | N = 77  Age = 6.56  Girls = 53.2% | TGMD-2 (OC, Loco, GM) | PSPMSC (Prox) | N/A |
| de Witte et al. (2022) | Netherlands | N = 1669  Age = 9.09  Girls = 45% | Athletic Skills Track (Loco) | PhSCS (Prox) | N/A |
| den Uil, Janssen, Busch, Kat, & Scholte (2023) | Netherlands | n = 2068  Age = 4-13  Girls = 49.4% | 4-Skills Scan (GM) | PSPC-PC (Prox) | ENERGY-questionnaire |
| D'Hondt et al. (2014) | Belgium | N = 754  Age = 8.3  Girls = 49.2% | KTK (Loco) | N/A | FPAQ |
| Dishman et al. (2006) | USA | N = 1250  Age = 17.66  Girls = 100% | N/A | PSDQ (Prox) | 3-Day Physical Activity Recall |
| DuBose, Gross McMillan, Wood, & Sisson (2018) | USA | N = 75  Age = 6.7  Girls = 42% | MABC-2 (OC, Loco, GM) | N/A | Accelerometer (Actigraph GT1M) |
| Duncan, Jones, O’Brien, Barnett, & Eyre (2018) | UK | N = 258  Age = 5.6  Girls = 46.1% | TGMD-2 (GM) | PSPMSC (GM) | N/A |
| Duncan & Stanley (2012) | UK | N = 58  Age = 10.7  Girls = 50% | Functional Movement Screen (GM) | N/A | Pedometer (New Lifestyles, NL2000) |
| Eberline, Judge, Walsh, & Hensley (2018) | USA | N = 42  Age = 10-11  Girls = 40.5% | N/A | CPAC (Prox) | PAQ-C |
| Emadirad, Temple, Field, Naylor, & Temple (2021) | Canada | N = 398  Age = 8.9  Girls = 50.5% | TGMD-2 (GM) | N/A | CAPE |
| Ensrud-Skraastad & Haga (2020) | Norway | N = 101  Age = 11.7  Girls = 47.5% | TMC (OC, Loco, GM) | SPPC-AC (Prox) | N/A |
| Estevan et al. (2022) | Spain | N = 124  Age = 7.4  Girls = 45.2% | TGMD-3 (OC, Loco, GM) | PSPMSC (OC, Loco, GM) | Accelerometer (ActiGraph wGT3X) |
| Estevan et al. (2021) | Spain | N = 91  Age = 11-14  Girls = nr | KTK (Loco) | PSPMSC (Loco) | N/A |
| Estevan et al. (2018) | Spain | N = 139  Age = 8.16  Girls = 48.2% | TGMD-3 (OC, Loco, GM) | PSPMSC (OC, Loco, GM) | N/A |
| Famelia, Tsuda, Bakhtiar, & Goodway (2018) | Indonesia | N = 66  Age = 5.04  Girls = 54.5% | TGMD-3 (OC, Loco) | PSPMSC (GM) | Accelerometer (ActiGraph wGT3X-BT) |
| Farmer, Belton, & O’Brien (2017) | Ireland | N = 160  Age = 10.69  Female = 100% | TGMD/TGMD-2/Get Skilled Get Active (OC, Loc) | PSC (Prox) | PAQ-C |
| Feitoza, Santos, Barnett, & Cattuzzo (2022) | Brazil | N = 379  Age = 8.2  Girls = 45.1% | TGMD-2 (OC, Loco, Co GM m) | PSPMSC (OC, Loco, GM) | LTEQ |
| Field & Temple (2017) | Canada | N = 400  Age = 9.5  Girls = 51% | TGMD-2 (OC, Loco) | N/A | CAPE |
| Fisher et al. (2005) | UK  MAGIC | N = 394  Age = 4.2  Girls = 47.0% | MABC (GM) | N/A | Accelerometer (ActiGraph 7164) |
| Foweather et al. (2015) | UK  Active Play Project | N = 99  Age = 4.6  Girls = 47.5% | TGMD-2 (OC, Loco, GM) | N/A | Accelerometer (Actigraph GT1M) |
| Fu & Burns (2018) | United States, ‘Zoom’ School | n = 66  Age = 11.6  Girls = 54.5% | TGMD-3 (GM) | PCSC-PC (Prox) | Yamax Digi-  Walker CW600, 7 days |
| Gilson, Cooke, & Mahoney (2005) | UK | N = 134  Age = 13.2  Girls 50.7% | N/A | CPSP-SC (Prox) | 4-day physical activity recall |
| Giuriato et al. (2022) | Italy | N = 104  Age = 8-11  Girls = NR | KTK (Loco) | N/A | PAQ-C |
| Goodway & Rudisill (1997) | USA | N =59  Age = 4.74  Female = 49.2% | TGMD (OC, Loco) | PSPC-PC (Prox) | N/A |
| Gråstén, Huhtiniemi, & Jaakkola (2022; Gråstén, Kolunsarka, Huhtiniemi, & Jaakkola (2022; Kolunsarka, Gråstén, Huhtiniemi, & Jaakkola (2021; Kolunsarka, Gråstén, Stodden, Huhtiniemi, & Jaakkola (2023) | Finland | N = 1121  Age = 11.26  Girls = 51.1% | Side-to-side jump, five-leap, throwing-catching (OC, Loco, GM) | PSPP-SC (Prox) | Accelerometer (ActiGraph wGT3X+) |
| Gu (2016) | USA | N = 256  Age = 5.37  Girls = 49.6% | PE Metrics^TM^ (OC, Loco, GM) | N/A | Accelerometer (Actical) |
| Gu, Chen, & Zhang (2019) | USA | N = 671  Age = 6.96  Girls = 46% | PE Metrics^TM^ (OC, Loco) | N/A | Accelerometer (Actical) |
| Gu, Thomas, & Chen (2017) | USA | N = 262  Age = 10.87  Girls = 49.2% | PE Metrics^TM^ (OC, Loco, GM) | Perceived competence during PE (Prox) | Pedometers, (Accusplit, Inc., Livermore, CA). |
| Gu et al. (2021) | USA | N = 342  Age = 8.4  Girls = 45.6% | PE Metrics^TM^ (OC, Loco) | N/A | Accelerometer (Actical) |
| Guan, Xiang, Land, & Hamilton (2023) | USA | N = 223  Age = 13.09  Girls = 48.4% | N/A | Perceived Physical Education Competence (Prox) | LTEQ |
| Guo, Schenkelberg, O’Neill, Dowda, & Pate (2018) | USA  CHAMPS | N = 227  Age = 4.15  Female = 51.1% | CHAMPS motor skill protocol (OC, Loco, GM) | N/A | Accelerometer (ActiGraph 7164) |
| Haapala et al. (2023) | Finland  PANIC | N = 189  Age = 7.67  Girls = 43% | Shuttle run (Loco) | N/A | Accelerometer/Heart Rate Monitor (ActiHeart) |
| Hall, Eyre, Oxford, & Duncan (2019) | UK | N = 38  Age = 5.41  Female = 37% | TGMD-2 (OC, Loco, GM) | PSPMSC (OC, Loco, GM) | Accelerometer (GENEActiv) |
| Hall, Eyre, Oxford, & Duncan (2018) | UK | N = 177  Age = 4.28  Female = 45.9% | TGMD-2 (OC, Loco, GM) | N/A | Accelerometer (GENEActiv) |
| Han, Li, Meng, Li, & Tong (2022) | China | N = 183  Age = 8.8  Girls = 52.5% | TGMD-3 (GM) | N/A | Pedometer |
| Hands, Larkin, Parker, Straker, & Perry (2009) | Australia  Raine | N = 1585  Age = 14.06  Girls = 48.6% | McCarron Assessment of Neuromuscular Development (GM) | N/A | Pedometer (Yamax Digiwalker SW200) |
| Hardman, Wanderley, Oliveira, & Barros (2017) | Brazil  ELOS-Pre | N = 665  Age = 6.3  Girls = 47.4% | KTK (Loco) | N/A | Parent reported child PA |
| Haugen, Ommundsen, & Seiler (2013) | Norway  Youth in Balance | N = 1839  Age = 15  Girls = 48.3% | N/A | SPPA-AC (Prox) | Youth Risk Behavior Study Physical Activity Questionnaire |
| Haugland, Nilsen, Okely, Aadland, & Aadland (2023) | Norway  ACTNOW | N = 952  Age = 4.3  Girls = 48.8% | TGMD-3 (OC, Loco) | N/A | Accelerometer (ActiGraph wGT3X+) |
| He, Ng, Cairney, Bedard, & Ha (2021) | Hong Kong | N =148  Age = 4.52  Girls = 43% | TGMD−2 (OC, Loco, GM) | Modified PSPMSC (OC, Loco, GM) | Accelerometer (ActiGraph wGT3X+) |
| Hill et al. (2022) | UK | N = 190  Age = 10.6  Girls = 57.9% | Get Skilled, Get Active (OC, Loco) | PSPP-SC (Prox) | Accelerometer (ActiGraph GT1M) |
| Houwen, Hartman, & Visscher (2009) | Netherlands | N = 48  Age = 8.5  Females = 33.3% | TGMD-2 (OC, Loco) | N/A | Accelerometer (ActiGraph GT1M) |
| Hulteen et al. (2018) | Australia  NEAT | N = 109  Age = 15.82  Girls = 49.5% | Lifelong Physical Activity Skills Battery (GM) | N/A | Accelerometer (GENEActiv) |
| Hulteen, True, & Pfeiffer (2020) | USA | N = 167  Age = 6-9  Girls = 55.7% | TGMD-2 (OC, Loco) | N/A | Pedometer (Yamax Digiwalker NL2000) |
| Humble, Yu, & Brown (2024) | Australia | N = 30  Age = 10.57  Female = 36.7% | BOT-2 (OC, Loco, GM) | PSPMSC (OC, Loco, GM) | N/A |
| Hume et al. (2008) | Australia  Switch-Play | N = 248  Age = 10.0  Girls = 50.4% | Overhand throw, two-handed strike, kick, sprint run, dodge, and vertical jump (OC, Loco, GM) | N/A | Accelerometer (ActiGraph 7164) |
| Huotari, Heikinaro-Johansson, Watt, & Jaakkola (2018) | Finland | N = 3736  Age = 15.2  Girls = 48.6% | Figure 8 dribbling, lateral jumping test, motor coordination test (OC, Loco, GM) | N/A | LTPA Questionnaire |
| Iivonen et al. (2013) | Finland | N = 37  Age = 4.2  Girls = 54.1% | APM Inventory (OC, Loco, GM) | N/A | Accelerometer (ActiGraph wGT3X) |
| Jaakkola, Huhtiniemi, et al. (2019) | Finland | N = 422  Age = 11.26  Girls = 58.3% | 5-leaps test, throwing-catching, two-legged jumping from side-to-side (Loco, OC) | PSPP-SC (Prox) | Accelerometer (ActiGraph wGT3X+) |
| Jaakkola & Washington (2013) | Finland | N = 152  Age = 13  Girls = 43.4% | Flamingo standing test, rolling test, leaping test, shuttle run, rope jumping, figure-eight dribbling, accuracy throwing (Loco, OC) | N/A | Health Behaviour in School-aged Children survey |
| Jarvis et al. (2018) | UK | N = 591  Age = 10.8  Female = 43.8% | GetSkilled: Get Active (GM) | PSPP-SC (Prox) | PAQ-C |
| Johnson, Wadsworth, Rudisill, Irwin, & Bridges (2022) | USA | N = 76  Age = nr  Female = 51.3% | TGMD-3 (Loco) | PCSC-PC (Prox) | N/A |
| Kaioglou, Dania, Kambas, & Venetsanou (2023) | Greece | N = 576  Age = 10.2  Girls = 53.5% | CAMSA (GM) | N/A | Pedometer (Omron HJ-720IT-E2) |
| Kalaja, Jaakkola, Liukkonen, & Watt (2010) | Finland | n = 316  Age = 13  Girls = 51.3% | Leaping Test, Flamingo Standing Test,  Figure 8 Dribbling Test (Loco) | PSPP - SC (Prox) | 7 Day Recall |
| Kambas et al. (2012) | Greece  Active Children-Active Schools | N = 232  Age = 5.37  Girls = 49.1% | BOT-SF (GM) | N/A | Pedometer (Omron Walking style pro HJ-720IT-E2) |
| Karuc et al. (2020) | Croatia  CRO-PALS | N = 725  Age = 16.6  Girls = 50.5% | Functional Movement Screen (GM) | N/A | SHAPES Questionnaire |
| Kavanagh, Issartel, & Moran (2019) | Ireland | N = 45  Age = 4.5  Female = 29% | MABC‐2 (OC, Loco, GM) | PSPMSC (GM) | N/A |
| Khodaverdi, Bahram, Khalaji, & Kazemnejad (2013; Khodaverdi, Bahram, & Robinson (2015) | Iran | N = 352  Age = 8.78  Girls = 100% | TGMD-2 (OC, Loco, GM) | SDQ-1-PA (Prox) | PAQ-C |
| King-Dowling, Proudfoot, Cairney, & Timmons (2020) | Canada  HOPP | N = 418  Age = 4.5  Girls = 49.8% | BOT-SF (GM) | N/A | Accelerometer (ActiGraph GT3X) |
| Lalor, Brown, & Murdolo (2016) | Australia | N = 55  Age = 10.04  Girls = 50.9% | BOT-2 (GM) | SPPC-AC (Prox) | N/A |
| Larouche, Boyer, Tremblay, & Longmuir (2013) | Canada  CAPL | N = 491  Age = 9-12  Female = 56.4% | Obstacle course (GM) | N/A | Pedometer (Digi-Walker SW200) |
| Laukkanen, Pesola, Havu, Sääkslahti, & Finni (2014) | Finland | N = 84  Age =5-9  Girls = 54.8% | KTK/underarm throw (OC, Loco) | N/A | Accelerometer (X6-1a) |
| Laukkanen, Pesola, Finni, & Sääkslahti (2017) | Finland | N = 64  Age = 6.33  Girls = 50% | KTK (Loco) | N/A | Accelerometer (X6-1a) |
| Laukkanen, Niemistö, Aunola, Barnett, & Sääkslahti (2023) | Finland  Skilled Kids  Active Family | n = 396  Age = 5.59  Girls = 51.8% | N/A | PSPMSC (GM) | Accelerometer (UKK RM42) |
| LeGear et al. (2012) | Canada | n = 267  Age = 5.75  Girls = 48% | TGMD-2 (Loco, OC, GM) | PSPC-PC (Prox) | N/A |
| Lin, Cherng, & Chen (2017) | Taiwan | N = 264  Age = 4.38  Girls = 25% | MABC-2 (GM) | N/A | Pre-PAQ |
| Liong, Ridgers, & Barnett (2015) | Australia | N = 136  Age = 6.5  Girls = 49% | TGMD-2 (OC, Loco, GM) | PSPMSC (OC, Loco, GM) | N/A |
| Lohbeck, von Keitz, Hohmann, & Daseking (2021) | Germany  Fulda Movement Check | N = 1082  Age = 7.32  Girls = 51.4% | German Motor Skills Test 6-18 (GM) | SDQ-1-PA (Prox) | N/A |
| Lopes & Rodrigues (2021) | Portugal | N = 1064  Age = 7.87  Girls = 49.8% | KTK (Loco) | N/A | LTEQ |
| Lopes, Barnett, & Rodrigues (2016) | Portugal | N = 101  Age = 4.9  Girls = 47.5% | MABC–2 (OC, Loco) | PSPMSC (GM) | Accelerometer (ActiGraph GT3X) |
| Lopes, Saraiva, Gonçalves, & Rodrigues (2018) | Portugal | n = 200  Age = 7.6  Girls = 55.5% | TGMD2 (OC, Loco) | PSPMSC (OC, Loco) | N/A |
| Loucaides, Chedzoy, Bennett, & Walshe (2004) | Cyprus | N = 256  Age = nr  Girls = nr | N/A | PCSC-PC (Prox) | PDPAR |
| Ma & Luo (2023) | China | N = 358  Age = 4.48  Girls = 45.8% | TGMD-2 (OC, Loco) | N/A | Accelerometer (ActiGraph GT3X+) |
| Matarma et al. (2018) | Finland  STEPS | N = 111  Age = 5.57  Girls = 54.5% | BOT-2 (OC, Loco) | N/A | Accelerometer (ActiGraph GT3X) |
| McGrane, Belton, Powell, & Issartel (2017) | Ireland | N = 395  Age = 13.78  Girls = 49.6% | TGMD-2/ Victorian Fundamental Movement Skills Manual (GM) | PSC (Prox) | N/A |
| McGrane, Powell, Belton, & Issartel (2018) | Ireland | N = 584  Age = 13.78  Girls = 52.4% | TGMD-2/ Victorian Fundamental Movement Skills Manual (OC, Loco) | PSC (OC, Loco) | Accelerometer (ActiGraph GT1M/GT3X/ GT3X+) |
| McIntyre, Parker, Chivers, & Hands (2018) | Australia | N = 201  Age = 6-8  Girls = 44.3% | Fundamental Movement Skills Teacher Resource Manual (GM) | SDQ-1-PA/App/PR (Prox) | Pedometers (Yamax SW-200) |
| Melby et al. (2021) | Denmark  CoSCIS-study | N = 654  Age = 6  Girls = 48% | KTK (Loco) | N/A | Accelerometer (Actigraph MTI 7164) |
| Menescardi & Estevan (2021) | Spain | N = 518  Age = 9.64  Girls = 46.5% | CAMSA (GM) | PMSC (GM) | PAQ-C |
| Miller, Eather, Duncan, & Lubans (2019) | Australia | N = 107  Age = 10.53  Girls = 57% | TGMD-3 (OC) | N/A | Accelerometer (ActiGraph GT3X) |
| Monacis, Trecroci, Invernizzi, & Colella (2022) | Italy | N = 1029  Age = 12.02  Female = 50.9% | N/A | PSESC (Prox) | PAQ-C |
| Morano, Colella, Robazza, Bortoli, & Capranica (2011) | Italy | N = 260  Age = 12.2  Girls = 46.2% | Standing long jump, overhead throw, shuttle run (OC, Loco) | PSDQ (Prox) | N/A |
| Morano, Bortoli, Ruiz, Campanozzi, & Robazza (2020) | Italy | N = 603  Age = 6-7  Girls = 49.9% | TGMD-2 (OC, Loco) | PSPMSC (Loco, OC) | N/A |
| Morgan, Okely, Cliff, Jones, & Baur (2008) | Australia | N = 137  Age = 8.3  Girls = 57.7% | TGMD-2 (OC, Loco, GM) | SPPC-AC/ PCSC-PC (Prox) | Accelerometer (ActiGraph 7164) |
| Morrison, Cairney, Eisenmann, Pfeiffer, & Gould (2018) | Canada  PHAST | N = 1881  Age = 9.9  Girls = 49.2% | BOTMP-SF (GM) | SPPC-AC (Prox) | PAQ |
| Nicolai Ré et al. (2020) | Brazil | N = 1017  Age = 7.21  Girls = 45% | TGMD-2/KTK (OC, Loco, GM) | N/A | Accelerometer (ActiGraph GT3X+) |
| Niemistö et al. (2019) | Finland  Get Skilled | N = 472  Age = 6.22  Girls = 47.7% | TGMD‐3 (OC, Loco) | PSPMSC (Loco, OC) | CLASS |
| Nilsen, Anderssen, Loftesnes, et al. (2020) | Norway  PRESPAS | N = 1081  Age = 4.7  Girls = 48% | TGMD-3/PGMQ (OC, Loco) | N/A | Accelerometer (ActiGraph GT3X+) |
| Noonan, Boddy, Knowles, & Fairclough (2018) | UK | N = 194  Age = 9.96  Girls = 55.2% | N/A | PSPSEQ-PC (Prox) | PAQ-C |
| Norman, Geer, & Looper (2019) | USA | N = 12  Age = 8-11  Female = 91.7% | TGMD-2 (OC, Loco) | N/A | Accelerometer (ActiGraph) |
| O’ Brien, Belton, & Issartel (2016) | Ireland  Y-PATH | N = 85  Age = 12.87  Girls = 36.5% | TGMD/TGMD-2/Get Skilled Get Active (OC, Loco, GM) | N/A | Accelerometer (ActiGraph GT1M/GT3X) |
| Paxton, Estabrooks, & Dzewaltowski (2004) | USA | N = 63  Age = 11.5  Girls = 66% | N/A | PCSC-PC (Prox) | PAQOC |
| Peers, Issartel, Behan, O'Connor, & Belton (2020) | Ireland  MWBW | N = 860  Age = 10.9  Female = 47.7% | TGMD-3 (GM) | PSPMSC (GM) | PACE |
| Pereira et al. (2020) | Portugal  Growth, Motor Development, and Cognition study | N = 307  Age = 7.7  Girls = 50.2% | KTK (Loco) | N/A | Accelerometer (ActiGraph GT3X+) |
| Pesce, Masci, Marchetti, Vannozzi, & Schmidt (2018) | Italy | N = 90  Age = 7.5  Girls = 45.6% | TGMD–2 (OC, Loco) | PSPMSC (OC, Loco) | N/A |
| Pérez & Sanz (2005) | Spain | N = 495  Age = 4-6  Girls = 50.1% | MABC (OC, Loco) | CPMCS (GM) | N/A |
| Queiroz et al. (2020) | Brazil  Longitudinal Study of Health and Wellbeing of Children in Preschool | N = 668  Age = 6.31  Girls = 52.4% | KTK (Loco) | N/A | Parent reported PA |
| Raudsepp, Liblik, & Hannus (2002) | Estonia | N = 253  Age = 13.6  Female = 47.0% | N/A | PSPP-SC (Prox) | 7-day physical activity recall |
| Raudsepp & Päll (2006) | Estonia | N = 133  Age = 7.6  Girls = 48.9% | Overhand throw, standing long jump (OC, Loco) | N/A | Accelerometer (Caltrac) |
| Reed, Metzker, & Phillips (2004) | USA | N = 217  Age = nr  Girls = 44.7% | Bass stick balance test, side-step agility test, AAHPERD passing test (OC, Loco) | N/A | Pedometer (New Lifestyles Digi-Walker SW401) |
| Reyes et al. (2019) | Portugal  Growth, Motor Development and Cognition Study | N = 344  Age = 4-9  Girls = 49.4% | KTK (Loco) | N/A | Accelerometer (ActiGraph GT3X+) |
| Robinson (2011) | USA | N= 119  Age= 4  Girls= 45.4% | TGMD-2 (OC, Loco, GM) | PSPCSA (GM) | N/A |
| Robinson, Wadsworth, & Peoples (2012) | USA | N = 34  Age = 4.75  Girls = 64.7% | TGMD-2 (OC, Loco) | PSPCSA – PC (Prox) | Pedometer (Yamax SW-200 Digiwalker) |
| Robinson & Palmer (2021) | USA | N = 87  Age = 4.5  Girls = 52% | TGMD-2 (OC, Loco, GM) | PSPMSC (OC, Loco, GM) | N/A |
| Rogers, Barnett, & Lander (2018) | Australia | N = 173  Age = 12.48  Girls = 100% | Victorian FMS Teachers’ Manual (Loco, OC, GM) | PSPMSC (Loco, OC, GM) | N/A |
| Rudisill, Mahar, & Meaney (1993) | USA | N = 218  Age = 9.90  Girls = 52.3% | 50y dash, shuttle run, broad jump, ball throw (Loco, OC) | Motor Perceived Competence Scale (GM) | N/A |
| Ryu, Lee, Liu, McDonough, & Gao (2021) | USA | N = 61  Age = 4.45  Girls = 54.1% | TGMD-2 (GM) | PSPC-PC (Prox) | Accelerometer (ActiGraph GT9X) |
| Ryu, Lee, Zeng, et al. (2021) | USA | N = 261  Age = 8.27  Girls = 51.3% | Run, kick,  and throw speed and hop and jump distance (GM) | PSPCSA-PC (Prox) | Accelerometer (ActiGraph GT3X+) |
| Sabiston & Crocker (2008) | Canada | N = 532  Age = 16.17  Girls = 61.3% | N/A | PCSPA (Prox) | LTEQ |
| Sallen, Andrä, Ludyga, Mücke, & Herrmann (2020) | Germany | N = 51  Age = 10.27  Girls = 47.1% | MOBAK-5-6 (OC, Loco) | SEMOK (OC, Loco) | Accelerometer (ActiGraph GT3X-BT) |
| Sallis, Alcaraz, McKenzie, & Hovell (1999) | USA | N = 732  Age = 9.54  Girls = 50.5% | N/A | Perceived physical activity competence (Prox) | Accelerometer (Caltrac) |
| Santos et al. (2018) | Brazil | N = 169  Age = 9.49  Girls = 61.2% | BOT-2 SF (OC, Loco) | N/A | Accelerometer (ActiGraph GT3X+) |
| Seabra et al. (2013) | Portugal | N = 683  Age = 8.83  Girls = 48.5% | N/A | PCSC-PC (Prox) | LTEQ |
| Silva-Santos, Santos, Duncan, Vale, & Mota (2019) | Portugal  PRESTYLE | N = 209  Age = 4.73  Girls = 52.1% | MABC-2 (GM) | N/A | Accelerometer (ActiGraph GT1M) |
| Silva-Santos et al. (2021) | Portugal  Preschool Physical Activity, Body Composition and Lifestyle Study | N = 54  Age = 4.27  Girls = 42.4% | MABC-2 (GM) | N/A | Accelerometer (ActiGraph GTM1) |
| Slykerman, Ridgers, Stevenson, & Barnett (2016) | Australia | N = 109  Age = 6.5  Female = 45.9% | TGMD-2 (OC, Loco) | PSPC (OC, Loco) | Accelerometer (ActiGraph GT3X+) |
| Smith, Fazeli, Wilkinson, & Clark (2021) | Iran/UK | N = 258  Age = 9.6  Girls = 51.6% | TGMD-2 (GM) | N/A | Accelerometer (GENEActiv) |
| Spessato, Gabbard, Robinson, & Valentini (2013) | Brazil | N = 178  Age = 5.36  Girls = 53.9% | TGMD-2 (GM) | PSPC-PC (Prox) | N/A |
| Spessato, Gabbard, & Valentini (2013) | Brazil | N = 264  Age = 7.44  Girls = 50% | TGMD-2 (OC, Loco, GM) | N/A | Pedometer (Yamax SW-200 Digiwalker) |
| Stein, Fisher, Berkey, & Colditz (2007) | USA  Growing Up Today Study (GUTS) | N = 8670  Age = 12.9  Girls = 60.7% | N/A | SPPC (Prox) | Self-reported questionnaire |
| Strotmeyer, Herrmann, & Kehne (2022) | Germany | N = 200  Age = 8.84  Girls = 58% | MOBAK 3–4 (OC, Loco) | SEMOK 3–4 (OC, Loco) | N/A |
| Sung, Loh, & Lin (2021) | Taiwan | N = 28  Age = 5.3  Female = 46.4% | MABC-2 (OC, Loco, GM) | N/A | Accelerometer (ActiGraph GT3X) |
| Syväoja et al. (2021) | Finland  Finnish Schools on the Move | N = 311  Age = 14.0  Girls = 59% | 5 leaps test, throwing and catching (GM) | N/A | Accelerometer (ActiGraph GT3X /GT3X+) |
| Telford, Telford, Olive, Cochrane, & Davey (2016) | Australia  Lifestyle of our Kids (LOOK) project | N = 555  Age = 8  Girls = 50.3% | Throw and catch skill test (OC) | Perceived Physical Education competence (Prox) | Pedometer (Walk 4 Life) |
| Temple, Crane, Brown, Williams, & Bell (2016) | Canada | N = 74  Age = 5.92  Girls = 45% | TGMD-2/Stork Stand (OC, Loco) | N/A | CAPE |
| Tietjens et al. (2020) | Australia – Germany  Universities Australia–Germany Joint Research Cooperation Scheme “Global Assessment of Children’s Motor Competence” | N = 358  Age = 8.34  Girls = 46.6% | TGMD-3 (OC, Loco) | PMSC (OC, Loco) | N/A |
| Tietjens et al. (2018) | Germany | N = 27  Age = 4.93  Girls = 40.7% | TGMD-3 (OC, Loco) | PSPSCC (Prox) | N/A |
| Toftegaard-Stoeckel, Groenfeldt, & Andersen (2010) | Denmark  Copenhagen School Child Intervention Study | N = 646  Age = 6.8  Girls = 47.7% | KTK (Loco) | Perceived bodily competence (Prox) | N/A |
| True, Brian, Goodway, & Stodden (2017) | USA | N = 411  Age = 4-11  Girls = 51% | TGMD-2 (GM) | PSPC-PC/SPPC-AC (Prox) | N/A |
| Tsuda, Goodway, Famelia, & Brian (2020) | USA | N = 72  Age = 4.38  Girls = 45.8% | TGMD-2 (OC, Loco) | PSPC-PC (Prox) | Accelerometer (ActiGraph GT3X-BT) |
| Valentini, Nobre, de Souza, & Duncan (2020) | Brazil | N = 900  Age = 3-10  Girls = 49.7% | TGMD-2 (OC, Loco) | PSPC-PC (Prox) | Pedometer (Yamax SW-200 Digiwalker) |
| Valentini, Souza, Souza, & Nobre (2023) | Brazil | N = 172  Age = 7.3  Female = 30.4% | TGMD-3 (OC, Loco) | PSPCSA (Prox) | Pedometer (Yamax SW-200 Digiwalker) |
| van Niekerk, du Toit, & Pienaar (2016) | South Africa  PAHL | N = 239  Age = 13-14  Girls = 59.0% | BOT-2-SF (OC, Loco, GM) | N/A | IPAQ |
| Vedul-Kjelsås, Sigmundsson, Stensdotter, & Haga (2012) | Norway | N = 67  Age = 11.46  Girls = 41.8% | MABC (Tot) | SPPC-AC (Prox) | N/A |
| Visagie, Coetzee, & Pienaar (2017) | South Africa  NWCHILD | N = 406  Age = 9.86  Girls = 100% | TGMD-2 (OC) | N/A | CLASS |
| Visser et al. (2020) | Australia | N = 134  Age = 6-7  Girls = 34.3% | N/A | PSPMSC (OC, Loco, GM) | Accelerometer (ActiGraph GT3X+) |
| Wang, Chia, Quek, & Liu (2006) | Singapore | N = 1155  Age = 11.27  Girls = 46.1% | N/A | PSPP-SC (Prox) | SAPAC |
| Webb, Benjamin, Gammon, McKee, & Biddle (2013) | UK | N = 238  Age = 15.3  Female = 100% | N/A | PSPP-SC (Prox) | Previous day physical activity recall |
| Weedon et al. (2023) | UK  Rhythmic Motor Learning in Children with Developmental Coordination Disorder study | N = 62  Age = 13-14  Girls = 49.7% | MABC2 (OC, Loco) | N/A | Accelerometer (Axivity AX3) |
| Welk & Schaben (2004) | USA | N = 25  Age= 8-12  Girls = 44% | N/A | SPPC-AC (Prox) | Accelerometer (Biotrianer activity monitor) |
| Wrotniak, Epstein, Dorn, Jones, & Kondilis (2006) | USA | N = 65  Age = 9.6  Girls = 52.3% | BOT-SF (GM) | N/A | Accelerometer (ActiGraph 7164) |
| Yli-Piipari, Gråstén, Huhtiniemi, Salin, & Jaakkola (2021) | Finland | N = 450  Age = 11.26  Females = 43.1% | Five-jump test, throwing and catching, two-legged jump (OC, Loco) | N/A | Accelerometer (ActiGraph GT3X+) |
| Zeng, Johnson, Boles, & Bellows (2019) | USA  Longitudinal Eating And Physical activity (LEAP) | N = 228  Age = 4.67  Girls = 56.1% | BOT-2 (OC, Loco) | PSPC-PC (Prox) | N/A |
| Zhang, Thomas, & Weiller (2015) | USA | N = 288  Age = 10-12  Girls = 48.3% | PE Metrics (OC) | PCS (OC) | PAQ-C |
| Zhang, Lee, Chu, Chen, & Gu (2020) | USA | N = 215  Age = 10.55  Girls = 48.4% | PE Metrics (OC) | PCS (OC) | PAQ-C |
| Ziviani et al. (2006) | Australia | N = 50  Age = 7.74  Girls = 48% | MABC (GM) | SDQ-1-PA (Prox) | Pedometer (Yamax SW-200 Digiwalker) |
| Lagged Association | | | | | |
| Baker & Davison (2011) | USA | n = 149  Age = 9  Girls = 100.0% | N/A | SPPA-AC (Prox) | Accelerometer (ActiGraph 7164) & Children’s’ Physical Activity scale |
| Barnett, Morgan, van Beurden, & Beard (2008) | Australia  Move It Groove It | n = 250  Age = 16.4  Female = 52.4% | Get Skilled Get Active (OC, Loco) | PSPP – SC (Prox) | Adolescent Physical Activity Recall Questionnaire |
| Barnett, Salmon, & Hesketh (2016) | Australia  InFANT | n = 116  Age = 3.5  Girls = 55.1% | TGMD-2 (OC, Loco, GM) | PSPMSC (OC, Loco, GM) | Accelerometer (ActiGraph GT1M) |
| Bürgi et al. (2011) | Switzerland  Ballabeina-Study | N = 217  Age = 5.2  Girls = 52% | Obstacle course, dynamic balance test (Loco) | N/A | Accelerometer (ActiGraph GT1M) |
| Burns et al. (2022) | USA | N = 440  Age = 8.9  Girls = 52.0% | TGMD-3 (GM) | N/A | Pedometer (Yamax DigiWalker CW600) |
| D'Hondt et al. (2014) | Belgium | N = 754  Age = 8.3  Girls = 49.2% | KTK (Loco) | N/A | FPAQ |
| Estevan et al. (2022) | Spain | N = 124  Age = 7.4  Girls = 45.2% | TGMD-3 (OC, Loco, GM) | PSPMSC (OC, Loco, GM) | Accelerometer (ActiGraph wGT3X) |
| Foulkes et al. (2022) | UK  Active Play Project | N = 75  Age = 4.6  Girls = 49.3% | TGMD-2 (OC, Loco, GM) | N/A | Accelerometer (ActiGraph wGT3X+) |
| Gråstén, Kolunsarka, et al. (2022; Jaakkola, Yli-Piipari, et al. (2019) | Finland | N = 491  Age = 11.27  Girls = 50.7% | Side-to-side jump, five-leap, throwing-catching (GM) | PSPP-SC (Prox) | Accelerometer (ActiGraph GT3X+) |
| Han et al. (2022) | China | N = 183  Age = 8.8  Girls = 52.5% | TGMD-3 (GM) | N/A | Pedometer |
| Hikihara et al. (2022) | Japan | n = 247  Age = 6.9  Girls = 38.5% | TGMD-2 (OC, Loco) | N/A | Acceleromter (Activestyle Pro 350-IT) |
| Jaakkola, Yli-Piipari, Huotari, Watt, & Liukkonen (2016) | Finland | N = 333  Age = 12.41  Girls = 60.0% | Flamingo standing test, leaping test, figure-8 test (GM) | N/A | IPAQ |
| Jaakkola, Hakonen, et al. (2019) | Finland  Finnish Schools on the Move | N = 336  Age = 12.03  Girls = 48.5% | 5-leaps test, throwing-catching (OC, Loco) | N/A | Accelerometer (ActiGraph wGT3X+) |
| Jekauc, Wagner, Herrmann, Hegazy, & Woll (2017) | Germany  MoMo | N = 698  Age = 14.2  Girls = 52% | Jumping side-to-side test, the single leg stance, and the backward balancing (Loco) | PSDQ (Prox) | MoMo-PAQ |
| Larsen, Kristensen, Junge, Rexen, & Wedderkopp (2015) | Denmark  CHAMPS-DK | N = 673  Age = 9.2  Girls = 56% | Backward balance, precision throw, shuttle run, vertical jump (OC, Loco) | N/A | Accelerometer (ActiGraph wGT3X) |
| Lopes et al. (2019) | Portugal  LabMed Physical Activity Study | N = 103  Age = 13.49  Girls = 51.5% | KTK (Loco) | N/A | Accelerometer (ActiGraph GT1M) |
| Melby et al. (2021) | Denmark  CoSCIS-study | N = 654  Age = 13  Girls = 48% | KTK (Loco) | N/A | Accelerometer (Actigraph MTI 7164) |
| Menescardi et al. (2023) | Spain | N = 361  Age = 8.92  Girls = 50.7% | CAMSA (GM) | PMSC (GM) | PAQ-C |
| Nilsen, Anderssen, Johannessen, et al. (2020) | Norway  PRESPAS | n = 230  Age = 4.7  Girls = 48% | TGMD-3 (OC, Loco) | N/A | Accelerometer (ActiGraph GT3X+) |
| Ryu, Lee, Liu, et al. (2021) | USA | N = 61  Age = 4.44  Girls = 54.1% | TGMD-2 (GM) | PSPC-PC (Prox) | Accelerometer (ActiGraph GT9X) |
| Ryu, Lee, Zeng, et al. (2021) | USA | n = 261  Age = 8.27  Girls = 51.3% | Run, kick, throw speeds; hop and jump distance (GM) | PSPCSA (Prox) | ActiGraph GT3X+ accelerometers (Pensacola, FL) |
| Sallen et al. (2020) | Germany | N = 51  Age = 10.27  Girls = 47.1% | MOBAK-5-6 (OC, Loco) | SEMOK (OC, Loco) | Accelerometer (ActiGraph GT3X-BT) |
| Schmutz et al. (2020) | Switzerland  SPLASHY | N = 550  Age = 3.9  Girls = 53% | ZNA 3-5 (GM) | N/A | Accelerometer (ActiGraph GT3X-BT) |
| Visser et al. (2020) | Australia | N = 134  Age = 6-7  Girls = 34.3% | N/A | PSPMSC (OC, Loco, GM) | Accelerometer (ActiGraph GT3X+) |

**Abbreviations:** AMC = Actual movement competency, AIQ-C = Athletic identity questionnaire competence subscale, AMPET-R = Acheivment Motivation for Learning in Physical Education, BOT = Bruininks-Oseretsky Test of Motor Proficiency, CAPE = Children's Assessment of Participation and Enjoyment, CLASS = Children’s Leisure Activities Study Survey, CPAC = Children’s Physical Activity Correlates, CPMCS = Children’s Perception of Motor Competence Scale, CPSP-SC = Children’s Physical Self-Perception Profile Sports Competence subscale, FPAQ = Flemish Physical Activity Questionnaire, G-DAS-C = Girls Disinclination for Physical Activity Competence Subscale, IPAQ = International Physical Activity Questionnaire, KTK = Körperkoordinationstest fur Kinder, LTEQ = Leisure Time Exercise Questionnaire, LTPA = Leisure time physical activity, MABC = Movement Assessment Battery for Children, MoMo-PAQ = Motorik-Modul Physical Activity Questionniare, PAQ-A = Physical Activity Questionnaire for Adolescents, PAQ-C = Physical Activity Questionnaire for Older Children, PCS = Perceived Competence Scale, PCSC-PC = Perceived Competence Scale for Children Physical Competence subscale, PCSPA = Perceived Competence Scales for Participating in Regular Physical Activity, PDMS-2 = Peabody Developmental Motor Scales, PMC = perceived movement competency, PMC-C = Perceived Motor Competence Questionnaire for Children, PhSCS = Physical Self-Confidence Scale, PMC-C = Perceived Motor Competence Questionnaire in Childhood, PMSC – Perceived Motor Skill Competence Questionnaire, PMSC-S = Perceived Motor Skill Competency Stability, PSESC = Physical Self-Efficacy Scale for Children, PSPC-PC = Pictorial Scale of Perceived Competence physical competence subscale, PSPMSC = Pictorial Scale of Perceived Movement Skill Competence, PSPSEQ-PC = Physical Self Perceptions and Self-Esteem Questionnaire Physical Competence subscale, PSC = Physical Self-Confidence Scale, PSDQ = Physical Self-Descriptive Questionnaire, PSPP-SC = Physical Self-Perception Profile Sports Competence subscale, PSPSCC = Pictorial Scale of Physical Self-Concept for Younger Children, SAPAC = Self-administered Physical Activity Checklist, SDQ-1-PA = Self-Description Questionnaire 1 Physical Ability subscale, SDQ-1-App = Self-Description Questionnaire 1 Physical Appearance Subscale, SDQ-1-PR = Self-Description Questionnaire 1 Peer Relationship subscale, SEMOK = SElbstwahrnehmung MOtorischer Kompetenzen, SHAPES = School Health Action, Planning, and Evaluation System, SPPA-AC = Self Perception Profile for Adolescents Athletic Competence subscale, SPPC-AC = Self-Perception Profile for Children Athletic Competence subscale, , YPAP = Youth PA Promotion, PSPCSA = Pictorial Scale of Perceived Competence and Social Acceptance, TGMD = Test of Gross Motor Development, ZNA = Zurich Neuromotor Assessment

**Articles included in the review**

Aadland, K. N., Moe, V. F., Aadland, E., Anderssen, S. A., Resaland, G. K., & Ommundsen, Y. (2017). Relationships between physical activity, sedentary time, aerobic fitness, motor skills and executive function and academic performance in children. *Mental Health and Physical Activity, 12*, 10-18. <https://doi.org/https://doi.org/10.1016/j.mhpa.2017.01.001>

Aalizadeh, B., Mohamadzadeh, H., & Hosseini, F. S. (2014). Fundamental movement skills among iranian primary school children. *Journal of Family and Reproductive Health, 8*(4), 155-159.

Adank, A. M., Van Kann, D. H. H., Remmers, T., Kremers, S. P. J., & Vos, S. B. (2021). Longitudinal perspectives on children’s physical activity patterns: “Do physical education–related factors matter?”. *Journal of Physical Activity and Health, 18*(10), 1199-1206. <https://doi.org/10.1123/jpah.2020-0859>

Afthentopoulou, A.-E., Venetsanou, F., Zounhia, A., & Petrogiannis, K. (2018). Physical activity, motor competence, and perceived physical competence: What is their relationship in children aged 6–9 years? *Human Movement, 19*(1), 51-56. <https://doi.org/10.5114/hm.2018.73612>

Amraei, M., & Azadian, E. (2021). Motor competence–related age and living environment in girls: A cross-sectional study. *Journal of Motor Learning and Development, 9*(3), 470-482. <https://doi.org/10.1123/jmld.2021-0010>

Anderson, C. B., Mâsse, L. C., Zhang, H., Coleman, K. J., & Chang, S. (2009). Contribution of athletic identity to child and adolescent physical activity. *American Journal of Preventive Medicine, 37*(3), 220-226. <https://doi.org/10.1016/j.amepre.2009.05.017>

Bai, Y., Chen, S., Vazou, S., Welk, G. J., & Schaben, J. (2015). Mediated effects of perceived competence on youth physical activity and sedentary behavior. *Research Quarterly for Exercise and Sport, 86*(4), 406-413. <https://doi.org/10.1080/02701367.2015.1087639>

Baker, B. L., & Davison, K. K. (2011). I know i can: A longitudinal examination of precursors and outcomes of perceived athletic competence among adolescent girls. *Journal of Physical Activity and Health, 8*(2), 192-199. <https://doi.org/10.1123/jpah.8.2.192>

Balaban, V. (2018). The relationship between objectively measured physical activity and fundamental motor skills in 8 to 11 years old children from the czech republic. *Montenegrin Journal of Sports Science and Medicine, 7*(2). <https://doi.org/10.26773/mjssm.180902>

Bardid, F., De Meester, A., Tallir, I., Cardon, G., Lenoir, M., & Haerens, L. (2016). Configurations of actual and perceived motor competence among children: Associations with motivation for sports and global self-worth. *Human Movement Science, 50*, 1-9. <https://doi.org/10.1016/j.humov.2016.09.001>

Barnett, L., Hinkley, T., Okely, A. D., & Salmon, J. (2013). Child, family and environmental correlates of children's motor skill proficiency. *Journal of Science and Medicine in Sport, 16*(4), 332-336. <https://doi.org/10.1016/j.jsams.2012.08.011>

Barnett, L. M., Morgan, P. J., Van Beurden, E., Ball, K., & Lubans, D. R. (2011). A reverse pathway? Actual and perceived skill proficiency and physical activity. *Medicine & Science in Sports & Exercise, 43*(5), 898-904. <https://doi.org/10.1249/MSS.0b013e3181fdfadd>

Barnett, L. M., Morgan, P. J., van Beurden, E., & Beard, J. R. (2008). Perceived sports competence mediates the relationship between childhood motor skill proficiency and adolescent physical activity and fitness: A longitudinal assessment. *International Journal of Behavioral Nutrition and Physical Activity, 5*(1), 40. <https://doi.org/10.1186/1479-5868-5-40>

Barnett, L. M., Ridgers, N. D., Hesketh, K., & Salmon, J. (2017). Setting them up for lifetime activity: Play competence perceptions and physical activity in young children. *Journal of Science and Medicine in Sport, 20*(9), 856-860. <https://doi.org/10.1016/j.jsams.2017.03.003>

Barnett, L. M., Ridgers, N. D., & Salmon, J. (2015). Associations between young children's perceived and actual ball skill competence and physical activity. *Journal of Science and Medicine in Sport, 18*(2), 167-171. <https://doi.org/10.1016/j.jsams.2014.03.001>

Barnett, L. M., Salmon, J., & Hesketh, K. D. (2016). More active pre-school children have better motor competence at school starting age: An observational cohort study. *BMC Public Health, 16*(1), 1068. <https://doi.org/10.1186/s12889-016-3742-1>

Barnett, L. M., Telford, R. M., Strugnell, C., Rudd, J., Olive, L. S., & Telford, R. D. (2019). Impact of cultural background on fundamental movement skill and its correlates. *Journal of Sports Sciences, 37*(5), 492-499. <https://doi.org/10.1080/02640414.2018.1508399>

Bernal, C. M. M., Lhuisset, L., Bru, N., Fabre, N., & Bois, J. (2024). Do physical activity, sedentary time, motor skills and aerobic fitness predict primary school children’s attention? Use of a data mining strategy. *International Journal of Sport and Exercise Psychology*, 1-18. <https://doi.org/10.1080/1612197X.2023.2239841>

Bezerra, T. A., Bandeira, P. F. R., de Souza Filho, A. N., Clark, C. C. T., Mota, J., Duncan, M. J., & de Lucena Martins, C. M. (2021). A network perspective on the relationship between moderate to vigorous physical activity and fundamental motor skills in early childhood. *Journal of Physical Activity and Health, 18*(7), 774-781. <https://doi.org/10.1123/jpah.2020-0218>

Blomqvist, M., Mononen, K., Tolvanen, A., & Konttinen, N. (2019). Objectively assessed vigorous physical activity and motor coordination are associated in 11-year old children. *Scandinavian Journal of Medicine & Science in Sports, 29*(10), 1629-1635. <https://doi.org/https://doi.org/10.1111/sms.13500>

Bois, J. E., Sarrazin, P. G., Brustad, R. J., Trouilloud, D. O., & Cury, F. (2005). Elementary schoolchildren's perceived competence and physical activity involvement: The influence of parents' role modelling behaviours and perceptions of their child's competence. *Psychology of Sport and Exercise, 6*(4), 381-397. <https://doi.org/https://doi.org/10.1016/j.psychsport.2004.03.003>

Bolger, L. A., Bolger, L. E., O’Neill, C., Coughlan, E., Lacey, S., O’Brien, W., & Burns, C. (2019). Fundamental movement skill proficiency and health among a cohort of irish primary school children. *Research Quarterly for Exercise and Sport, 90*(1), 24-35. <https://doi.org/10.1080/02701367.2018.1563271>

Bolger, L. E., Bolger, L. A., O'Neill, C., Coughlan, E., O'Brien, W., Lacey, S., & Burns, C. (2018). Accuracy of children's perceived skill competence and its association with physical activity. *Journal of Physical Activity and Health*, 1-8. <https://doi.org/10.1123/jpah.2017-0371>

Boucher, B. H., Doescher, S. M., & Sugawara, A. I. (1993). Preschool children's motor development and self-concept. *Perceptual and Motor Skills, 76*(1), 11-17. <https://doi.org/10.2466/pms.1993.76.1.11>

Breau, B., Brandes, B., Wright, M. N., Buck, C., Vallis, L. A., & Brandes, M. (2021). Association of individual motor abilities and accelerometer-derived physical activity measures in preschool-aged children. *Journal for the Measurement of Physical Behaviour, 4*(3), 227-235. <https://doi.org/10.1123/jmpb.2020-0065>

Bremer, E., Graham, J. D., Bedard, C., Rodriguez, C., Kriellaars, D., & Cairney, J. (2020). The association between playfun and physical activity: A convergent validation study. *Research Quarterly for Exercise and Sport, 91*(2), 179-187. <https://doi.org/10.1080/02701367.2019.1652723>

Brian, A., Bardid, F., Barnett, L. M., Deconinck, F. J. A., Lenoir, M., & Goodway, J. D. (2018). Actual and perceived motor competence levels of belgian and united states preschool children. *Journal of Motor Learning and Development, 6*(s2), S320-S336. <https://doi.org/10.1123/jmld.2016-0071>

Brian, A., Taunton, S., Shortt, C., Pennell, A., & Sacko, R. (2019). Predictors of physical activity for preschool children with and without disabilities from socioeconomically disadvantaged settings. *Adapt Phys Activ Q, 36*(1), 77-90. <https://doi.org/10.1123/apaq.2017-0191>

Bürgi, F., Meyer, U., Granacher, U., Schindler, C., Marques-Vidal, P., Kriemler, S., & Puder, J. J. (2011). Relationship of physical activity with motor skills, aerobic fitness and body fat in preschool children: A cross-sectional and longitudinal study (ballabeina). *International Journal of Obesity, 35*(7), 937-944. <https://doi.org/10.1038/ijo.2011.54>

Burns, R., Brusseau, T., & Hannon, J. (2017). Multivariate associations among health-related fitness, physical activity, and tgmd-3 test items in disadvantaged children from low-income families. *Perceptual and Motor Skills, 124*(1), 86-104. <https://doi.org/10.1177/0031512516672118>

Burns, R. D., Bai, Y., Byun, W., Colotti, T. E., Pfledderer, C. D., Kwon, S., & Brusseau, T. A. (2022). Bidirectional relationships of physical activity and gross motor skills before and after summer break: Application of a cross-lagged panel model. *Journal of Sport and Health Science, 11*(2), 244-251. <https://doi.org/https://doi.org/10.1016/j.jshs.2020.07.001>

Burns, R. D., & Fu, Y. (2018). Testing the motor competence and health-related variable conceptual model: A path analysis. *Journal of Functional Morphology and Kinesiology, 3*(4). <https://doi.org/10.3390/jfmk3040061>

Capio, C. M., & Eguia, K. F. (2021). Movement skills, perception, and physical activity of young children: A mediation analysis. *Pediatrics International, 63*(4), 442-447. <https://doi.org/10.1111/ped.14436>

Capio, C. M., Sit, C. H., Eguia, K. F., & Abernethy, B. (2014). Physical activity and movement skills proficiency of young filipino children. *Pediatrics International, 56*(4), 651-653. <https://doi.org/10.1111/ped.12436>

Carballo-Fazanes, A., Díaz-Pereira, M. P., Fernández-Villarino, M. A., Abelairas-Gómez, C., & Rey, E. (2023). Physical activity in kindergarten, fundamental movement skills, and screen time in spanish preschool children. *Psychology in the Schools, 60*(9), 3318-3328. <https://doi.org/https://doi.org/10.1002/pits.22925>

Carcamo-Oyarzun, J., Estevan, I., & Herrmann, C. (2020). Association between actual and perceived motor competence in school children. *International Journal of Environmental Research and Public Health, 17*(10). <https://doi.org/10.3390/ijerph17103408>

Carvalho, A. S., Bohn, L., Abdalla, P. P., Ramos, N. C., Borges, F. G., Mota, J., & Machado, D. R. L. (2021). The associations of objectively measured physical activity, fundamental motor skills and time in sedentary behavior in children: A cross-sectional study. *Perceptual and Motor Skills, 128*(6), 2507-2526. <https://doi.org/10.1177/00315125211038731>

Chan, C. H. S., Ha, A. S. C., Ng, J. Y. Y., & Lubans, D. R. (2019). Associations between fundamental movement skill competence, physical activity and psycho-social determinants in hong kong chinese children. *Journal of Sports Sciences, 37*(2), 229-236. <https://doi.org/10.1080/02640414.2018.1490055>

Chaves, R., Baxter-Jones, A., Gomes, T., Souza, M., Pereira, S., & Maia, J. (2015). Effects of individual and school-level characteristics on a child's gross motor coordination development. *International Journal of Environmental Research and Public Health, 12*(8), 8883-8896. <https://doi.org/10.3390/ijerph120808883>

Cliff, D. P., Okely, A. D., Smith, L. M., & McKeen, K. (2009). Relationships between fundamental movement skills and objectively measured physical activity in preschool children. *Pediatric Exercise Science, 21*(4), 436-449. <https://doi.org/10.1123/pes.21.4.436>

Cohen, K. E., Morgan, P. J., Plotnikoff, R. C., Callister, R., & Lubans, D. R. (2014). Fundamental movement skills and physical activity among children living in low-income communities: A cross-sectional study. *International Journal of Behavioral Nutrition and Physical Activity, 11*(1), 49. <https://doi.org/10.1186/1479-5868-11-49>

Coker, C. A., & Herrick, B. (2021). Functional movement proficiency’s association to actual and perceived motor competence. *Journal of Motor Learning and Development, 9*(1), 28-37. <https://doi.org/10.1123/jmld.2020-0002>

Cook, C. J., Howard, S. J., Scerif, G., Twine, R., Kahn, K., Norris, S. A., & Draper, C. E. (2019). Associations of physical activity and gross motor skills with executive function in preschool children from low-income south african settings. *Developmental Science, 22*(5), e12820. <https://doi.org/10.1111/desc.12820>

Coppens, E., De Meester, A., Deconinck, F. J. A., De Martelaer, K., Haerens, L., Bardid, F., . . . D'Hondt, E. (2021). Differences in weight status and autonomous motivation towards sports among children with various profiles of motor competence and organized sports participation. *Children (Basel), 8*(2). <https://doi.org/10.3390/children8020156>

Craft, L. L., Pfeiffer, K. A., & Pivarnik, J. M. (2003). Predictors of physical competence in adolescent girls. *Journal of Youth and Adolescence, 32*(6), 431-438. <https://doi.org/10.1023/A:1025986318306>

Craike, M. J., Polman, R., Eime, R., Symons, C., Harvey, J., & Payne, W. (2014). Associations between behavior regulation, competence, physical activity, and health for adolescent females. *Journal of Physical Activity and Health, 11*(2), 410-418. <https://doi.org/10.1123/jpah.2012-0070>

Crane, J. R., Foley, J. T., Naylor, P.-J., & Temple, V. A. (2017). Longitudinal change in the relationship between fundamental motor skills and perceived competence: Kindergarten to grade 2. *Sports, 5*(3). doi:10.3390/sports5030059

Crane, J. R., Foley, J. T., & Temple, V. A. (2023). The influence of perceptions of competence on motor skills and physical activity in middle childhood: A test of mediation. *International Journal of Environmental Research and Public Health, 20*(9). doi:10.3390/ijerph20095648

Crocker, P. R. E., Eklund, R. C., & Kowalski, K. C. (2000). Children's physical activity and physical self-perceptions. *Journal of Sports Sciences, 18*(6), 383-394. <https://doi.org/10.1080/02640410050074313>

Cumming, S. P., Standage, M., Loney, T., Gammon, C., Neville, H., Sherar, L. B., & Malina, R. M. (2011). The mediating role of physical self-concept on relations between biological maturity status and physical activity in adolescent females. *Journal of Adolescence, 34*(3), 465-473. <https://doi.org/https://doi.org/10.1016/j.adolescence.2010.06.006>

D'Hondt, E., Deforche, B., Gentier, I., Verstuyf, J., Vaeyens, R., De Bourdeaudhuij, I., . . . Lenoir, M. (2014). A longitudinal study of gross motor coordination and weight status in children. *Obesity, 22*(6), 1505-1511. <https://doi.org/https://doi.org/10.1002/oby.20723>

Davison, K. K., Schmalz, D. L., & Downs, D. S. (2010). Hop, skip … no! Explaining adolescent girls’ disinclination for physical activity. *Annals of Behavioral Medicine, 39*(3), 290-302. <https://doi.org/10.1007/s12160-010-9180-x>

de Bruijn, A. G. M., & van der Wilt, F. (2023). Social acceptance in physical education and the regular classroom: Perceived motor competency and frequency and type of sports participation. *Children, 10*(3). doi:10.3390/children10030568

De Meester, A., Maes, J., Stodden, D., Cardon, G., Goodway, J., Lenoir, M., & Haerens, L. (2016). Identifying profiles of actual and perceived motor competence among adolescents: Associations with motivation, physical activity, and sports participation. *Journal of Sports Sciences, 34*(21), 2027-2037. <https://doi.org/10.1080/02640414.2016.1149608>

De Meester, A., Stodden, D., Brian, A., True, L., Cardon, G., Tallir, I., & Haerens, L. (2016). Associations among elementary school children’s actual motor competence, perceived motor competence, physical activity and bmi: A cross-sectional study. *PLOS ONE, 11*(10), e0164600. <https://doi.org/10.1371/journal.pone.0164600>

de Oliveira Martins, A., Flôres, F., Valentini, N., & Copetti, F. (2023). What do the parents perceive, and how it affects children’s motor competence? An exploratory study in 5 to 11 years old south brazilian children. *Motricidade, 19*(1), 41-48.

de Witte, A., Hoeboer, J., Coppens, E., Lenoir, M., Platvoet, S., de Niet, M., . . . de Meester, A. (2022). A variable- and person-centered approach to further understand the relationship between actual and perceived motor competence in children. *Journal of Teaching in Physical Education, 41*(3), 391-400. <https://doi.org/10.1123/jtpe.2021-0038>

den Uil, A. R., Janssen, M., Busch, V., Kat, I. T., & Scholte, R. H. J. (2023). The relationships between children’s motor competence, physical activity, perceived motor competence, physical fitness and weight status in relation to age. *PLOS ONE, 18*(4), e0278438. <https://doi.org/10.1371/journal.pone.0278438>

Dishman, R. K., Hales, D. P., Pfeiffer, K. A., Felton, G. A., Saunders, R., Ward, D. S., . . . Pate, R. R. (2006). Physical self-concept and self-esteem mediate cross-sectional relations of physical activity and sport participation with depression symptoms among adolescent girls. *Health Psychology, 25*(3), 396-407. <https://doi.org/10.1037/0278-6133.25.3.396>

DuBose, K. D., Gross McMillan, A., Wood, A. P., & Sisson, S. B. (2018). Joint relationship between physical activity, weight status, and motor skills in children aged 3 to 10 years. *Perceptual and Motor Skills, 125*(3), 478-492. <https://doi.org/10.1177/0031512518767008>

Duncan, M. J., Jones, V., O’Brien, W., Barnett, L. M., & Eyre, E. L. J. (2018). Self-perceived and actual motor competence in young british children. *Perceptual and Motor Skills, 125*(2), 251-264. <https://doi.org/10.1177/0031512517752833>

Duncan, M. J., & Stanley, M. (2012). Functional movement is negatively associated with weight status and positively associated with physical activity in british primary school children. *Journal of Obesity, 2012*, 697563. <https://doi.org/10.1155/2012/697563>

Eberline, A., Judge, L. W., Walsh, A., & Hensley, L. D. (2018). Relationship of enjoyment, perceived competence, and cardiorespiratory fitness to physical activity levels of elementary school children. *Physical Educator, 75*(3), 394-413. <https://doi.org/https://doi.org/10.18666/TPE-2018-V75-I3-8161>

Emadirad, E., Temple, B. W. N., Field, S. C., Naylor, P.-J., & Temple, V. A. (2021). Motor skills and participation in middle childhood: A direct path for boys, a mediated path for girls. *Journal of Physical Activity and Health, 18*(3), 318-324. <https://doi.org/10.1123/jpah.2020-0296>

Ensrud-Skraastad, O. K., & Haga, M. (2020). Associations between motor competence, physical self-perception and autonomous motivation for physical activity in children. *Sports, 8*(9). doi:10.3390/sports8090120

Estevan, I., Clark, C., Molina-García, J., Menescardi, C., Barton, V., & Queralt, A. (2022). Longitudinal association of movement behaviour and motor competence in childhood: A structural equation model, compositional, and isotemporal substitution analysis. *Journal of Science and Medicine in Sport, 25*(8), 661-666. <https://doi.org/https://doi.org/10.1016/j.jsams.2022.05.010>

Estevan, I., Menescardi, C., Castillo, I., Molina-García, J., García-Massó, X., & Barnett, L. M. (2021). Perceived movement skill competence in stability: Validity and reliability of a pictorial scale in early adolescents. *Scandinavian Journal of Medicine & Science in Sports, 31*(5), 1135-1143. <https://doi.org/https://doi.org/10.1111/sms.13928>

Estevan, I., Molina-García, J., Bowe, S. J., Álvarez, O., Castillo, I., & Barnett, L. M. (2018). Who can best report on children's motor competence: Parents, teachers, or the children themselves? *Psychology of Sport and Exercise, 34*, 1-9. <https://doi.org/https://doi.org/10.1016/j.psychsport.2017.09.002>

Famelia, R., Tsuda, E., Bakhtiar, S., & Goodway, J. D. (2018). Relationships among perceived and actual motor skill competence and physical activity in indonesian preschoolers. *Journal of Motor Learning and Development, 6*(s2), S403-S423. <https://doi.org/10.1123/jmld.2016-0072>

Farmer, O., Belton, S., & O’Brien, W. (2017). The relationship between actual fundamental motor skill proficiency, perceived motor skill confidence and competence, and physical activity in 8–12-year-old irish female youth. *Sports, 5*(4). doi:10.3390/sports5040074

Feitoza, A. H. P., Santos, A. B. D., Barnett, L. M., & Cattuzzo, M. T. (2022). Motor competence, physical activity, and perceived motor competence: A relational systems approach. *Journal of Sports Sciences, 40*(21), 2371-2383. <https://doi.org/10.1080/02640414.2022.2158268>

Field, S. C., & Temple, V. A. (2017). The relationship between fundamental motor skill proficiency and participation in organized sports and active recreation in middle childhood. *Sports, 5*(2). doi:10.3390/sports5020043

Fisher, A., Reilly, J. J., Kelly, L. A., Montgomery, C., Williamson, A., Paton, J. Y., & Grant, S. (2005). Fundamental movement skills and habitual physical activity in young children. *Medicine & Science in Sports & Exercise, 37*(4). Retrieved from <https://journals.lww.com/acsm-msse/fulltext/2005/04000/fundamental_movement_skills_and_habitual_physical.23.aspx>

Foulkes, J. D., Knowles, Z., Fairclough, S. J., Stratton, G., O’Dwyer, M. V., & Foweather, L. (2022). Is foundational movement skill competency important for keeping children physically active and at a healthy weight? *International Journal of Environmental Research and Public Health, 19*(1). doi:10.3390/ijerph19010105

Foweather, L., Knowles, Z., Ridgers, N. D., O’Dwyer, M. V., Foulkes, J. D., & Stratton, G. (2015). Fundamental movement skills in relation to weekday and weekend physical activity in preschool children. *Journal of Science and Medicine in Sport, 18*(6), 691-696. <https://doi.org/https://doi.org/10.1016/j.jsams.2014.09.014>

Fu, Y., & Burns, R. D. (2018). Gross motor skills and school day physical activity: Mediating effect of perceived competence. *Journal of Motor Learning and Development, 6*(2), 287-300. <https://doi.org/10.1123/jmld.2017-0043>

Gilson, N., Cooke, C., & Mahoney, C. (2005). Adolescent physical self‐perceptions, sport/exercise and lifestyle physical activity. *Health Education, 105*(6), 437-450.

Giuriato, M., Lovecchio, N., Carnevale Pellino, V., Mieszkowski, J., Kawczyński, A., Nevill, A., & Biino, V. (2022). Gross motor coordination and their relationship with body mass and physical activity level during growth in children aged 8–11 years old: A longitudinal and allometric approach. *PeerJ, 10*, e13483. <https://doi.org/10.7717/peerj.13483>

Goodway, J. D., & Rudisill, M. E. (1997). Perceived physical competence and actual motor skill competence of african american preschool children. *Adapted Physical Activity Quarterly, 14*(4), 314-326. <https://doi.org/10.1123/apaq.14.4.314>

Gråstén, A., Huhtiniemi, M., & Jaakkola, T. (2022). School-age children’s actual motor competence and perceived physical competence: A 3-yr follow-up. *Medicine & Science in Sports & Exercise, 54*(6). Retrieved from <https://journals.lww.com/acsm-msse/fulltext/2022/06000/school_age_children_s_actual_motor_competence_and.15.aspx>

Gråstén, A., Kolunsarka, I., Huhtiniemi, M., & Jaakkola, T. (2022). Developmental associations of actual motor competence and perceived physical competence with health-related fitness in schoolchildren over a four-year follow-up. *Psychology of Sport and Exercise, 63*, 102279. <https://doi.org/https://doi.org/10.1016/j.psychsport.2022.102279>

Gu, X. (2016). Fundamental motor skill, physical activity, and sedentary behavior in socioeconomically disadvantaged kindergarteners. *Psychology, Health & Medicine, 21*(7), 871-881. <https://doi.org/10.1080/13548506.2015.1125007>

Gu, X., Chen, S., & Zhang, X. (2019). Young hispanic and non-hispanic children’s fundamental motor competence and physical activity behaviors. *Journal of Motor Learning and Development, 7*(2), 180-193. <https://doi.org/10.1123/jmld.2018-0003>

Gu, X., Tamplain, P. M., Chen, W., Zhang, T., Keller, M. J., & Wang, J. (2021). A mediation analysis of the association between fundamental motor skills and physical activity during middle childhood. *Children, 8*(2). doi:10.3390/children8020064

Gu, X., Thomas, K. T., & Chen, Y.-L. (2017). The role of perceived and actual motor competency on children’s physical activity and cardiorespiratory fitness during middle childhood. *Journal of Teaching in Physical Education, 36*(4), 388-397. <https://doi.org/10.1123/jtpe.2016-0192>

Guan, J., Xiang, P., Land, W. M., & Hamilton, X. D. (2023). The roles of perceived physical education competence, enjoyment, and persistence on middle school students’ physical activity engagement. *Perceptual and Motor Skills, 130*(4), 1781-1796. <https://doi.org/10.1177/00315125231178341>

Guo, H., Schenkelberg, M. A., O’Neill, J. R., Dowda, M., & Pate, R. R. (2018). How does the relationship between motor skill performance and body mass index impact physical activity in preschool children? *Pediatric Exercise Science, 30*(2), 266-272. <https://doi.org/10.1123/pes.2017-0074>

Haapala, E. A., Widlund, A., Poikkeus, A.-M., Lima, R. A., Brage, S., Aunio, P., & Lakka, T. A. (2023). Cross-lagged associations between physical activity, motor performance, and academic skills in primary school children. *Medicine & Science in Sports & Exercise, 55*(8). Retrieved from <https://journals.lww.com/acsm-msse/fulltext/2023/08000/cross_lagged_associations_between_physical.13.aspx>

Hall, C. J. S., Eyre, E. L. J., Oxford, S. W., & Duncan, M. J. (2018). Relationships between motor competence, physical activity, and obesity in british preschool aged children. *Journal of Functional Morphology and Kinesiology, 3*(4). doi:10.3390/jfmk3040057

Hall, C. J. S., Eyre, E. L. J., Oxford, S. W., & Duncan, M. J. (2019). Does perception of motor competence mediate associations between motor competence and physical activity in early years children? *Sports, 7*(4). doi:10.3390/sports7040077

Han, S., Li, B., Meng, S., Li, Y., & Tong, W. (2022). Bi-directionality between physical activity within school and fundamental movement skills in school-aged students: A cross-lagged study. *International Journal of Environmental Research and Public Health, 19*(13). doi:10.3390/ijerph19137624

Hands, B., Larkin, D., Parker, H., Straker, L., & Perry, M. (2009). The relationship among physical activity, motor competence and health-related fitness in 14-year-old adolescents. *Scandinavian Journal of Medicine & Science in Sports, 19*(5), 655-663. <https://doi.org/https://doi.org/10.1111/j.1600-0838.2008.00847.x>

Hardman, C. M., Wanderley, R. d. S., Oliveira, E. S. A. d., & Barros, M. V. G. d. (2017). Relationship between physical activity and bmi with level of motor coordination performance in schoolchildren. *Revista Brasileira de Cineantropometria & Desempenho Humano, 19*, 50-61.

Haugen, T., Ommundsen, Y., & Seiler, S. (2013). The relationship between physical activity and physical self-esteem in adolescents: The role of physical fitness indices. *Pediatric Exercise Science, 25*(1), 138-153. <https://doi.org/10.1123/pes.25.1.138>

Haugland, E. S., Nilsen, A. K. O., Okely, A. D., Aadland, K. N., & Aadland, E. (2023). Multivariate physical activity association patterns for fundamental motor skills and physical fitness in preschool children aged 3–5 years. *Journal of Sports Sciences, 41*(7), 654-667. <https://doi.org/10.1080/02640414.2023.2232219>

He, Q., Ng, J. Y. Y., Cairney, J., Bedard, C., & Ha, A. S. C. (2021). Association between physical activity and fundamental movement skills in preschool-aged children: Does perceived movement skill competence mediate this relationship? *International Journal of Environmental Research and Public Health, 18*(3). doi:10.3390/ijerph18031289

Hikihara, Y., Watanabe, M., Aoyama, T., Wakabayashi, H., Hanawa, S., Omi, N., & Tanaka, S. (2022). Does earlier acquisition of motor competence promote pubertal physical activity in japanese elementary school children: A 4-year follow-up study. *Journal of Sports Sciences, 40*(18), 2000-2009. <https://doi.org/10.1080/02640414.2022.2124710>

Hill, P. J., McNarry, M. A., Lester, L., Foweather, L., Boddy, L. M., Fairclough, S. J., & Mackintosh, K. A. (2022). Sex-related differences in the association of fundamental movement skills and health and behavioral outcomes in children. *Journal of Motor Learning and Development, 10*(1), 27-40. <https://doi.org/10.1123/jmld.2020-0066>

Houwen, S., Hartman, E., & Visscher, C. (2009). Physical activity and motor skills in children with and without visual impairments. *Medicine & Science in Sports & Exercise, 41*(1). Retrieved from <https://journals.lww.com/acsm-msse/fulltext/2009/01000/physical_activity_and_motor_skills_in_children.11.aspx>

Hulteen, R. M., Barnett, L. M., Morgan, P. J., Robinson, L. E., Barton, C. J., Wrotniak, B. H., & Lubans, D. R. (2018). Determining the initial predictive validity of the lifelong physical activity skills battery. *Journal of Motor Learning and Development, 6*(2), 301-314. <https://doi.org/10.1123/jmld.2017-0036>

Hulteen, R. M., True, L., & Pfeiffer, K. A. (2020). Differences in associations of product- and process-oriented motor competence assessments with physical activity in children. *Journal of Sports Sciences, 38*(4), 375-382. <https://doi.org/10.1080/02640414.2019.1702279>

Humble, A., Yu, M.-L., & Brown, T. (2024). Association between parent-proxy-reported and child-self-reported perceptions of children’s motor competence and children’s performance-based motor skill abilities. *Scandinavian Journal of Occupational Therapy, 31*(1), 2274883. <https://doi.org/10.1080/11038128.2023.2274883>

Hume, C., Okely, A., Bagley, S., Telford, A., Booth, M., Crawford, D., & Salmon, J. (2008). Does weight status influence associations between children's fundamental movement skills and physical activity? *Research Quarterly for Exercise and Sport, 79*(2), 158-165. <https://doi.org/10.1080/02701367.2008.10599479>

Huotari, P., Heikinaro-Johansson, P., Watt, A., & Jaakkola, T. (2018). Fundamental movement skills in adolescents: Secular trends from 2003 to 2010 and associations with physical activity and bmi. *Scandinavian Journal of Medicine & Science in Sports, 28*(3), 1121-1129. <https://doi.org/https://doi.org/10.1111/sms.13028>

Iivonen, K. S., Sääkslahti, A. K., Mehtälä, A., Villberg, J. J., Tammelin, T. H., Kulmala, J. S., & Poskiparta, M. (2013). Relationship between fundamental motor skills and physical activity in 4-year-old preschool children. *Perceptual and Motor Skills, 117*(2), 627-646. <https://doi.org/10.2466/10.06.PMS.117x22z7>

Jaakkola, T., Hakonen, H., Kankaanpää, A., Joensuu, L., Kulmala, J., Kallio, J., . . . Tammelin, T. H. (2019). Longitudinal associations of fundamental movement skills with objectively measured physical activity and sedentariness during school transition from primary to lower secondary school. *Journal of Science and Medicine in Sport, 22*(1), 85-90. <https://doi.org/https://doi.org/10.1016/j.jsams.2018.07.012>

Jaakkola, T., Huhtiniemi, M., Salin, K., Seppälä, S., Lahti, J., Hakonen, H., & Stodden, D. F. (2019). Motor competence, perceived physical competence, physical fitness, and physical activity within finnish children. *Scandinavian Journal of Medicine & Science in Sports, 29*(7), 1013-1021. <https://doi.org/https://doi.org/10.1111/sms.13412>

Jaakkola, T., & Washington, T. (2013). The relationship between fundamental movement skills and self-reported physical activity during finnish junior high school. *Physical Education and Sport Pedagogy, 18*(5), 492-505. <https://doi.org/10.1080/17408989.2012.690386>

Jaakkola, T., Yli-Piipari, S., Huhtiniemi, M., Salin, K., Seppälä, S., Hakonen, H., & Gråstén, A. (2019). Longitudinal associations among cardiorespiratory and muscular fitness, motor competence and objectively measured physical activity. *Journal of Science and Medicine in Sport, 22*(11), 1243-1248. <https://doi.org/https://doi.org/10.1016/j.jsams.2019.06.018>

Jaakkola, T., Yli-Piipari, S., Huotari, P., Watt, A., & Liukkonen, J. (2016). Fundamental movement skills and physical fitness as predictors of physical activity: A 6-year follow-up study. *Scandinavian Journal of Medicine & Science in Sports, 26*(1), 74-81. <https://doi.org/https://doi.org/10.1111/sms.12407>

Jarvis, S., Williams, M., Rainer, P., Jones, E. S., Saunders, J., & Mullen, R. (2018). Interpreting measures of fundamental movement skills and their relationship with health-related physical activity and self-concept. *Measurement in Physical Education and Exercise Science, 22*(1), 88-100. <https://doi.org/10.1080/1091367X.2017.1391816>

Jekauc, D., Wagner, M. O., Herrmann, C., Hegazy, K., & Woll, A. (2017). Does physical self-concept mediate the relationship between motor abilities and physical activity in adolescents and young adults? *PLOS ONE, 12*(1), e0168539. <https://doi.org/10.1371/journal.pone.0168539>

Johnson, J. L., Wadsworth, D. D., Rudisill, M. E., Irwin, J. M., & Bridges, C. (2022). Does skill performance influence young children’s perceived physical competence? *Perceptual and Motor Skills, 129*(5), 1396-1412. <https://doi.org/10.1177/00315125221116756>

Kaioglou, V., Dania, A., Kambas, A., & Venetsanou, F. (2023). Associations of motor competence, cardiorespiratory fitness, and physical activity: The mediating role of cardiorespiratory fitness. *Research Quarterly for Exercise and Sport, 94*(2), 361-367. <https://doi.org/10.1080/02701367.2021.1991559>

Kalaja, S., Jaakkola, T., Liukkonen, J., & Watt, A. (2010). Fundamental movement skills and motivational factors influencing engagement in physical activity. *Perceptual and Motor Skills, 111*(1), 115-128. <https://doi.org/10.2466/06.10.25.PMS.111.4.115-128>

Kambas, A., Michalopoulou, M., Fatouros, I. G., Christoforidis, C., Manthou, E., Giannakidou, D., . . . Zimmer, R. (2012). The relationship between motor proficiency and pedometer-determined physical activity in young children. *Pediatric Exercise Science, 24*(1), 34-44. <https://doi.org/10.1123/pes.24.1.34>

Karuc, J., Mišigoj-Duraković, M., Marković, G., Hadžić, V., Duncan, M. J., Podnar, H., & Sorić, M. (2020). Movement quality in adolescence depends on the level and type of physical activity. *Physical Therapy in Sport, 46*, 194-203. <https://doi.org/https://doi.org/10.1016/j.ptsp.2020.09.006>

Kavanagh, J., Issartel, J., & Moran, K. (2019). How actual motor competence and perceived motor competence influence motor-skill engagement of a novel cycling task. *Scandinavian Journal of Medicine & Science in Sports, 29*(10), 1583-1590. <https://doi.org/https://doi.org/10.1111/sms.13492>

Khodaverdi, Z., Bahram, A., Khalaji, H., & Kazemnejad, A. (2013). Motor skill competence and perceived motor competence: Which best predicts physical activity among girls? *Iranian Journal of Public Health, 42*(10), 1145-1150.

Khodaverdi, Z., Bahram, A., & Robinson, L. E. (2015). Correlates of physical activity behaviours in young iranian girls. *Child: Care, Health and Development, 41*(6), 903-910. <https://doi.org/https://doi.org/10.1111/cch.12253>

King-Dowling, S., Proudfoot, N. A., Cairney, J., & Timmons, B. W. (2020). Motor competence, physical activity, and fitness across early childhood. *Medicine & Science in Sports & Exercise, 52*(11). Retrieved from <https://journals.lww.com/acsm-msse/fulltext/2020/11000/motor_competence,_physical_activity,_and_fitness.8.aspx>

Kolunsarka, I., Gråstén, A., Huhtiniemi, M., & Jaakkola, T. (2021). Development of children’s actual and perceived motor competence, cardiorespiratory fitness, physical activity, and bmi. *Medicine & Science in Sports & Exercise, 53*(12). Retrieved from <https://journals.lww.com/acsm-msse/fulltext/2021/12000/development_of_children_s_actual_and_perceived.21.aspx>

Kolunsarka, I., Gråstén, A., Stodden, D., Huhtiniemi, M., & Jaakkola, T. (2023). Impact of motor competence profiles on adolescents’ physical activity and cardiorespiratory fitness across four years. *Medicine & Science in Sports & Exercise, 55*(9). Retrieved from <https://journals.lww.com/acsm-msse/fulltext/2023/09000/impact_of_motor_competence_profiles_on.11.aspx>

Lalor, A., Brown, T., & Murdolo, Y. (2016). Relationship between children's performance-based motor skills and child, parent, and teacher perceptions of children's motor abilities using self/informant-report questionnaires. *Australian Occupational Therapy Journal, 63*(2), 105-116. <https://doi.org/https://doi.org/10.1111/1440-1630.12253>

Larouche, R., Boyer, C., Tremblay, M. S., & Longmuir, P. (2013). Physical fitness, motor skill, and physical activity relationships in grade 4 to 6 children. *Applied Physiology, Nutrition, and Metabolism, 39*(5), 553-559. <https://doi.org/10.1139/apnm-2013-0371>

Larsen, L. R., Kristensen, P. L., Junge, T., Rexen, C. T., & Wedderkopp, N. (2015). Motor performance as predictor of physical activity in children: The champs study-dk. *Medicine & Science in Sports & Exercise, 47*(9). Retrieved from <https://journals.lww.com/acsm-msse/fulltext/2015/09000/motor_performance_as_predictor_of_physical.11.aspx>

Laukkanen, A., Niemistö, D., Aunola, K., Barnett, L. M., & Sääkslahti, A. (2023). Child perceived motor competence as a moderator between physical activity parenting and child objectively measured physical activity. *Psychology of Sport and Exercise, 67*, 102444. <https://doi.org/https://doi.org/10.1016/j.psychsport.2023.102444>

Laukkanen, A., Pesola, A., Havu, M., Sääkslahti, A., & Finni, T. (2014). Relationship between habitual physical activity and gross motor skills is multifaceted in 5- to 8-year-old children. *Scandinavian Journal of Medicine & Science in Sports, 24*(2), e102-e110. <https://doi.org/https://doi.org/10.1111/sms.12116>

Laukkanen, A., Pesola, A. J., Finni, T., & Sääkslahti, A. (2017). Body mass index in the early years in relation to motor coordination at the age of 5–7 years. *Sports, 5*(3). doi:10.3390/sports5030049

LeGear, M., Greyling, L., Sloan, E., Bell, R. I., Williams, B.-L., Naylor, P.-J., & Temple, V. A. (2012). A window of opportunity? Motor skills and perceptions of competence of children in kindergarten. *International Journal of Behavioral Nutrition and Physical Activity, 9*(1), 29. <https://doi.org/10.1186/1479-5868-9-29>

Lin, L.-Y., Cherng, R.-J., & Chen, Y.-J. (2017). Relationship between time use in physical activity and gross motor performance of preschool children. *Australian Occupational Therapy Journal, 64*(1), 49-57. <https://doi.org/https://doi.org/10.1111/1440-1630.12318>

Liong, G. H. E., Ridgers, N. D., & Barnett, L. M. (2015). Associations between skill perceptions and young children's actual fundamental movement skills. *Perceptual and Motor Skills, 120*(2), 591-603. <https://doi.org/10.2466/10.25.PMS.120v18x2>

Lohbeck, A., von Keitz, P., Hohmann, A., & Daseking, M. (2021). Children's physical self-concept, motivation, and physical performance: Does physical self-concept or motivation play a mediating role? *Frontiers in Psychology, 12*. <https://doi.org/10.3389/fpsyg.2021.669936>

Lopes, L., Silva Mota, J. A. P., Moreira, C., Abreu, S., Agostinis Sobrinho, C., Oliveira-Santos, J., . . . Santos, R. (2019). Longitudinal associations between motor competence and different physical activity intensities: Labmed physical activity study. *Journal of Sports Sciences, 37*(3), 285-290. <https://doi.org/10.1080/02640414.2018.1497424>

Lopes, V., Barnett, L., & Rodrigues, L. (2016). Is there an association among actual motor competence, perceived motor competence, physical activity, and sedentary behavior in preschool children? *Journal of Motor Learning and Development, 4*(2), 129-141. <https://doi.org/10.1123/jmld.2015-0012>

Lopes, V. P., & Rodrigues, L. P. (2021). The role of physical fitness on the relationship between motor competence and physical activity: Mediator or moderator? *Journal of Motor Learning and Development, 9*(3), 456-469. <https://doi.org/10.1123/jmld.2020-0070>

Lopes, V. P., Saraiva, L., Gonçalves, C., & Rodrigues, L. P. (2018). Association between perceived and actual motor competence in portuguese children. *Journal of Motor Learning and Development, 6*(s2), S366-S377. <https://doi.org/10.1123/jmld.2016-0059>

Loucaides, C. A., Chedzoy, S. M., Bennett, N., & Walshe, K. (2004). Correlates of physical activity in a cypriot sample of sixth-grade children. *Pediatric Exercise Science, 16*(1), 25-36. <https://doi.org/10.1123/pes.16.1.25>

Ma, F.-F., & Luo, D.-M. (2023). Relationships between physical activity, fundamental motor skills, and body mass index in preschool children. *Frontiers in Public Health, 11*. <https://doi.org/10.3389/fpubh.2023.1094168>

Matarma, T., Lagström, H., Hurme, S., Tammelin, T. H., Kulmala, J., Barnett, L. M., & Koski, P. (2018). Motor skills in association with physical activity, sedentary time, body fat, and day care attendance in 5-6-year-old children—the steps study. *Scandinavian Journal of Medicine & Science in Sports, 28*(12), 2668-2676. <https://doi.org/https://doi.org/10.1111/sms.13264>

McGrane, B., Belton, S., Powell, D., & Issartel, J. (2017). The relationship between fundamental movement skill proficiency and physical self-confidence among adolescents. *Journal of Sports Sciences, 35*(17), 1709-1714. <https://doi.org/10.1080/02640414.2016.1235280>

McGrane, B., Powell, D., Belton, S., & Issartel, J. (2018). Investigation into the relationship between adolescents’ perceived and actual fundamental movement skills and physical activity. *Journal of Motor Learning and Development, 6*(s2), S424-S439. <https://doi.org/10.1123/jmld.2016-0073>

McIntyre, F., Parker, H., Chivers, P., & Hands, B. (2018). Actual competence, rather than perceived competence, is a better predictor of physical activity in children aged 6-9 years. *Journal of Sports Sciences, 36*(13), 1433-1440. <https://doi.org/10.1080/02640414.2017.1390243>

Melby, P. S., Elsborg, P., Nielsen, G., Lima, R. A., Bentsen, P., & Andersen, L. B. (2021). Exploring the importance of diversified physical activities in early childhood for later motor competence and physical activity level: A seven-year longitudinal study. *BMC Public Health, 21*(1), 1492. <https://doi.org/10.1186/s12889-021-11343-1>

Menescardi, C., De Meester, A., Álvarez, O., Castillo, I., Haerens, L., & Estevan, I. (2023). The mediational role of motivation in the model of motor development in childhood: A longitudinal study. *Psychology of Sport and Exercise, 66*, 102398. <https://doi.org/https://doi.org/10.1016/j.psychsport.2023.102398>

Menescardi, C., & Estevan, I. (2021). Parental and peer support matters: A broad umbrella of the role of perceived social support in the association between children’s perceived motor competence and physical activity. *International Journal of Environmental Research and Public Health, 18*(12). doi:10.3390/ijerph18126646

Miller, A., Eather, N., Duncan, M., & Lubans, D. R. (2019). Associations of object control motor skill proficiency, game play competence, physical activity and cardiorespiratory fitness among primary school children. *Journal of Sports Sciences, 37*(2), 173-179. <https://doi.org/10.1080/02640414.2018.1488384>

Monacis, D., Trecroci, A., Invernizzi, P. L., & Colella, D. (2022). Can enjoyment and physical self-perception mediate the relationship between bmi and levels of physical activity? Preliminary results from the regional observatory of motor development in italy. *International Journal of Environmental Research and Public Health, 19*(19). doi:10.3390/ijerph191912567

Morano, M., Bortoli, L., Ruiz, M. C., Campanozzi, A., & Robazza, C. (2020). Actual and perceived motor competence: Are children accurate in their perceptions? *PLOS ONE, 15*(5), e0233190. <https://doi.org/10.1371/journal.pone.0233190>

Morano, M., Colella, D., Robazza, C., Bortoli, L., & Capranica, L. (2011). Physical self-perception and motor performance in normal-weight, overweight and obese children. *Scandinavian Journal of Medicine & Science in Sports, 21*(3), 465-473. <https://doi.org/https://doi.org/10.1111/j.1600-0838.2009.01068.x>

Morgan, P. J., Okely, A. D., Cliff, D. P., Jones, R. A., & Baur, L. A. (2008). Correlates of objectively measured physical activity in obese children. *Obesity, 16*(12), 2634-2641. <https://doi.org/https://doi.org/10.1038/oby.2008.463>

Morrison, K. M., Cairney, J., Eisenmann, J., Pfeiffer, K., & Gould, D. (2018). Associations of body mass index, motor performance, and perceived athletic competence with physical activity in normal weight and overweight children. *Journal of Obesity, 2018*, 3598321. <https://doi.org/10.1155/2018/3598321>

Nicolai Ré, A. H., Okely, A. D., Logan, S. W., da Silva, M. M. L. M., Cattuzzo, M. T., & Stodden, D. F. (2020). Relationship between meeting physical activity guidelines and motor competence among low-income school youth. *Journal of Science and Medicine in Sport, 23*(6), 591-595. <https://doi.org/https://doi.org/10.1016/j.jsams.2019.12.014>

Niemistö, D., Barnett, L. M., Cantell, M., Finni, T., Korhonen, E., & Sääkslahti, A. (2019). Socioecological correlates of perceived motor competence in 5- to 7-year-old finnish children. *Scandinavian Journal of Medicine & Science in Sports, 29*(5), 753-765. <https://doi.org/https://doi.org/10.1111/sms.13389>

Nilsen, A. K. O., Anderssen, S. A., Johannessen, K., Aadland, K. N., Ylvisaaker, E., Loftesnes, J. M., & Aadland, E. (2020). Bi-directional prospective associations between objectively measured physical activity and fundamental motor skills in children: A two-year follow-up. *International Journal of Behavioral Nutrition and Physical Activity, 17*(1), 1. <https://doi.org/10.1186/s12966-019-0902-6>

Nilsen, A. K. O., Anderssen, S. A., Loftesnes, J. M., Johannessen, K., Ylvisaaker, E., & Aadland, E. (2020). The multivariate physical activity signature associated with fundamental motor skills in preschoolers. *Journal of Sports Sciences, 38*(3), 264-272. <https://doi.org/10.1080/02640414.2019.1694128>

Noonan, R. J., Boddy, L. M., Knowles, Z. R., & Fairclough, S. J. (2018). Predisposing, reinforcing and enabling factors for physical activity in boys and girls from socially disadvantaged communities. *Health Education Journal, 78*(2), 149-162. <https://doi.org/10.1177/0017896918792690>

Norman, C., Geer, W., & Looper, J. (2019). Motor competency and physical activity in elementary school aged children who participate in nontraditional sports *Journal of Physical Education and Sport, 19*(2).

O’ Brien, W., Belton, S., & Issartel, J. (2016). The relationship between adolescents’ physical activity, fundamental movement skills and weight status. *Journal of Sports Sciences, 34*(12), 1159-1167. <https://doi.org/10.1080/02640414.2015.1096017>

Paxton, R. J., Estabrooks, P. A., & Dzewaltowski, D. (2004). Attraction to physical activity mediates the relationship between perceived competence and physical activity in youth. *Research Quarterly for Exercise and Sport, 75*(1), 107-111. <https://doi.org/10.1080/02701367.2004.10609139>

Peers, C., Issartel, J., Behan, S., O'Connor, N., & Belton, S. (2020). Movement competence: Association with physical self-efficacy and physical activity. *Human Movement Science, 70*, 102582. <https://doi.org/https://doi.org/10.1016/j.humov.2020.102582>

Pereira, S., Reyes, A., Moura-Dos-Santos, M. A., Santos, C., Gomes, T. N., Tani, G., . . . Maia, J. (2020). Why are children different in their moderate-to-vigorous physical activity levels? A multilevel analysis. *Jornal de Pediatria, 96*(2), 225-232. <https://doi.org/https://doi.org/10.1016/j.jped.2018.10.013>

Pérez, L. M. R., & Sanz, J. L. G. (2005). New measure of perceived motor competence for children ages 4 to 6 years. *Perceptual and Motor Skills, 101*(1), 131-148. <https://doi.org/10.2466/pms.101.1.131-148>

Pesce, C., Masci, I., Marchetti, R., Vannozzi, G., & Schmidt, M. (2018). When children’s perceived and actual motor competence mismatch: Sport participation and gender differences. *Journal of Motor Learning and Development, 6*(s2), S440-S460. <https://doi.org/10.1123/jmld.2016-0081>

Queiroz, D. d. R., Aguilar, J. A., Martins Guimarães, T. G., Hardman, C. M., Lima, R. A., Duncan, M. J., . . . de Barros, M. V. G. (2020). Association between body mass index, physical activity and motor competence in children: Moderation analysis by different environmental contexts. *Annals of Human Biology, 47*(5), 417-424. <https://doi.org/10.1080/03014460.2020.1779815>

Raudsepp, L., Liblik, R., & Hannus, A. (2002). Children’s and adolescents’ physical self-perceptions as related to moderate to vigorous physical activity and physical fitness. *Pediatric Exercise Science, 14*(1), 97-106. <https://doi.org/10.1123/pes.14.1.97>

Raudsepp, L., & Päll, P. (2006). The relationship between fundamental motor skills and outside-school physical activity of elementary school children. *Pediatric Exercise Science, 18*(4), 426-435. <https://doi.org/10.1123/pes.18.4.426>

Reed, J. A., Metzker, A., & Phillips, D. A. (2004). Relationships between physical activity and motor skills in middle school children. *Perceptual and Motor Skills, 99*(2), 483-494. <https://doi.org/10.2466/pms.99.2.483-494>

Reyes, A. C., Chaves, R., Baxter-Jones, A. D. G., Vasconcelos, O., Barnett, L. M., Tani, G., . . . Maia, J. (2019). Modelling the dynamics of children’s gross motor coordination. *Journal of Sports Sciences, 37*(19), 2243-2252. <https://doi.org/10.1080/02640414.2019.1626570>

Robinson, L. E. (2011). The relationship between perceived physical competence and fundamental motor skills in preschool children. *Child: Care, Health and Development, 37*(4), 589-596. <https://doi.org/https://doi.org/10.1111/j.1365-2214.2010.01187.x>

Robinson, L. E., & Palmer, K. K. (2021). Examining the psychometric properties of the digital scale of perceived motor competence in young children. *Scandinavian Journal of Medicine & Science in Sports, 31*(12), 2272-2281. <https://doi.org/https://doi.org/10.1111/sms.14042>

Robinson, L. E., Wadsworth, D. D., & Peoples, C. M. (2012). Correlates of school-day physical activity in preschool students. *Research Quarterly for Exercise and Sport, 83*(1), 20-26. <https://doi.org/10.1080/02701367.2012.10599821>

Rogers, V., Barnett, L. M., & Lander, N. (2018). The relationship between fundamental movement skills and physical self-perception among adolescent girls. *Journal of Motor Learning and Development, 6*(s2), S378-S390. <https://doi.org/10.1123/jmld.2017-0041>

Rudisill, M. E., Mahar, M. T., & Meaney, K. S. (1993). The relationship between children's perceived and actual motor competence. *Perceptual and Motor Skills, 76*(3), 895-906. <https://doi.org/10.2466/pms.1993.76.3.895>

Ryu, S., Lee, J. E., Liu, W., McDonough, D. J., & Gao, Z. (2021). Investigating relationships between preschool children’s perceived competence, motor skills, and physical activity: A cross-lagged panel model. *Journal of Clinical Medicine, 10*(23). doi:10.3390/jcm10235620

Ryu, S., Lee, J. E., Zeng, N., Stodden, D., McDonough, D. J., Liu, W., & Gao, Z. (2021). Bidirectional relationships among children’s perceived competence, motor skill competence, physical activity, and cardiorespiratory fitness across one school year. *BioMed Research International, 2021*, 1704947. <https://doi.org/10.1155/2021/1704947>

Sabiston, C. M., & Crocker, P. R. E. (2008). Examining an integrative model of physical activity and healthy eating self-perceptions and behaviors among adolescents. *Journal of Adolescent Health, 42*(1), 64-72. <https://doi.org/https://doi.org/10.1016/j.jadohealth.2007.08.005>

Sallen, J., Andrä, C., Ludyga, S., Mücke, M., & Herrmann, C. (2020). School children’s physical activity, motor competence, and corresponding self-perception: A longitudinal analysis of reciprocal relationships. *Journal of Physical Activity and Health, 17*(11), 1083-1090. <https://doi.org/10.1123/jpah.2019-0507>

Sallis, J. F., Alcaraz, J. E., McKenzie, T. L., & Hovell, M. F. (1999). Predictors of change in children’s physical activity over 20 months: Variations by gender and level of adiposity. *American Journal of Preventive Medicine, 16*(3), 222-229. <https://doi.org/https://doi.org/10.1016/S0749-3797(98)00154-8>

Santos, M. O., Barbosa, D. G., Junior, G. J. F., Silva, R. C., Pelegrini, A., & Felden, É. P. G. (2018). Capacity of objective measures of physical activity to predict brazilian children’s low motor proficiency. *Perceptual and Motor Skills, 125*(4), 669-681. <https://doi.org/10.1177/0031512518774993>

Schmutz, E. A., Leeger-Aschmann, C. S., Kakebeeke, T. H., Zysset, A. E., Messerli-Bürgy, N., Stülb, K., . . . Kriemler, S. (2020). Motor competence and physical activity in early childhood: Stability and relationship. *Frontiers in Public Health, 8*. <https://doi.org/10.3389/fpubh.2020.00039>

Seabra, A. C., Seabra, A. F., Mendonça, D. M., Brustad, R., Maia, J. A., Fonseca, A. M., & Malina, R. M. (2013). Psychosocial correlates of physical activity in school children aged 8–10 years. *European Journal of Public Health, 23*(5), 794-798. <https://doi.org/10.1093/eurpub/cks149>

Silva-Santos, S., Santos, A., Duncan, M., Vale, S., & Mota, J. (2019). Association between moderate and vigorous physical activity and gross motor coordination in preschool children. *Journal of Motor Learning and Development, 7*(2), 273-285. <https://doi.org/10.1123/jmld.2017-0056>

Silva-Santos, S., Santos, A., Martins, C., Duncan, M., Lagoa, M. J., Vale, S., & Mota, J. (2021). Associations between motor competence, moderate-to-vigorous physical activity, and body mass index among preschoolers over 1 year. *Journal of Physical Activity and Health, 18*(7), 832-837. <https://doi.org/10.1123/jpah.2020-0356>

Slykerman, S., Ridgers, N. D., Stevenson, C., & Barnett, L. M. (2016). How important is young children's actual and perceived movement skill competence to their physical activity? *Journal of Science and Medicine in Sport, 19*(6), 488-492. <https://doi.org/https://doi.org/10.1016/j.jsams.2015.07.002>

Smith, E., Fazeli, F., Wilkinson, K., & Clark, C. C. T. (2021). Physical behaviors and fundamental movement skills in british and iranian children: An isotemporal substitution analysis. *Scandinavian Journal of Medicine & Science in Sports, 31*(2), 398-404. <https://doi.org/https://doi.org/10.1111/sms.13837>

Spessato, B. C., Gabbard, C., Robinson, L., & Valentini, N. C. (2013). Body mass index, perceived and actual physical competence: The relationship among young children. *Child: Care, Health and Development, 39*(6), 845-850. <https://doi.org/https://doi.org/10.1111/cch.12014>

Spessato, B. C., Gabbard, C., & Valentini, N. C. (2013). The role of motor competence and body mass index in children’s activity levels in physical education classes. *Journal of Teaching in Physical Education, 32*(2), 118-130. <https://doi.org/10.1123/jtpe.32.2.118>

Stein, C., Fisher, L., Berkey, C., & Colditz, G. (2007). Adolescent physical activity and perceived competence: Does change in activity level impact self-perception? *Journal of Adolescent Health, 40*(5), 462.e461-462.e468. <https://doi.org/https://doi.org/10.1016/j.jadohealth.2006.11.147>

Strotmeyer, A., Herrmann, C., & Kehne, M. (2022). A longitudinal analysis of reciprocal relationships between actual and perceived motor competencies and physical self-concept in primary-school age children. *Psychology of Sport and Exercise, 63*, 102269. <https://doi.org/https://doi.org/10.1016/j.psychsport.2022.102269>

Sung, Y.-S., Loh, S. C., & Lin, L.-Y. (2021). Physical activity and motor performance: A comparison between young children with and without autism spectrum disorder. *Neuropsychiatric disease and treatment*, 3743-3751.

Syväoja, H. J., Kankaanpää, A., Hakonen, H., Inkinen, V., Kulmala, J., Joensuu, L., . . . Tammelin, T. H. (2021). How physical activity, fitness, and motor skills contribute to math performance: Working memory as a mediating factor. *Scandinavian Journal of Medicine & Science in Sports, 31*(12), 2310-2321. <https://doi.org/https://doi.org/10.1111/sms.14049>

Telford, R. M., Telford, R. D., Olive, L. S., Cochrane, T., & Davey, R. (2016). Why are girls less physically active than boys? Findings from the look longitudinal study. *PLOS ONE, 11*(3), e0150041. <https://doi.org/10.1371/journal.pone.0150041>

Temple, V. A., Crane, J. R., Brown, A., Williams, B.-L., & Bell, R. I. (2016). Recreational activities and motor skills of children in kindergarten. *Physical Education and Sport Pedagogy, 21*(3), 268-280. <https://doi.org/10.1080/17408989.2014.924494>

Tietjens, M., Barnett, L. M., Dreiskämper, D., Holfelder, B., Utesch, T. O., Lander, N., . . . Schott, N. (2020). Conceptualising and testing the relationship between actual and perceived motor performance: A cross-cultural comparison in children from australia and germany. *Journal of Sports Sciences, 38*(17), 1984-1996. <https://doi.org/10.1080/02640414.2020.1766169>

Tietjens, M., Dreiskaemper, D., Utesch, T., Schott, N., Barnett, L. M., & Hinkley, T. (2018). Pictorial scale of physical self-concept for younger children (p-psc-c): A feasibility study. *Journal of Motor Learning and Development, 6*(s2), S391-S402. <https://doi.org/10.1123/jmld.2016-0088>

Toftegaard-Stoeckel, J., Groenfeldt, V., & Andersen, L. B. (2010). Children's self-perceived bodily competencies and associations with motor skills, body mass index, teachers' evaluations, and parents' concerns. *Journal of Sports Sciences, 28*(12), 1369-1375. <https://doi.org/10.1080/02640414.2010.510845>

True, L., Brian, A., Goodway, J., & Stodden, D. (2017). Relationships between product-and process-oriented measures of motor competence and perceived competence. *Journal of Motor Learning and Development, 5*(2), 319-335. <https://doi.org/10.1123/jmld.2016-0042>

Tsuda, E., Goodway, J. D., Famelia, R., & Brian, A. (2020). Relationship between fundamental motor skill competence, perceived physical competence and free-play physical activity in children. *Research Quarterly for Exercise and Sport, 91*(1), 55-63. <https://doi.org/10.1080/02701367.2019.1646851>

Valentini, N. C., Nobre, G. C., de Souza, M. S., & Duncan, M. J. (2020). Are bmi, self-perceptions, motor competence, engagement, and fitness related to physical activity in physical education lessons? *Journal of Physical Activity and Health, 17*(5), 493-500. <https://doi.org/10.1123/jpah.2019-0532>

Valentini, N. C., Souza, P. S. d., Souza, M. S. d., & Nobre, G. C. (2023). Individual and environmental parameters in children with and without developmental coordination disorder: Associations with physical activity and body mass index. *Frontiers in Pediatrics, 11*. <https://doi.org/10.3389/fped.2023.1202488>

van Niekerk, L.-L., du Toit, D., & Pienaar, A. E. (2016). The correlation between motor proficiency and physical activity in senior phase learners in the potchefstroom area. *Health SA Gesondheid, 21*, 348-355. <https://doi.org/https://doi.org/10.1016/j.hsag.2016.05.001>

Vedul-Kjelsås, V., Sigmundsson, H., Stensdotter, A. K., & Haga, M. (2012). The relationship between motor competence, physical fitness and self-perception in children. *Child: Care, Health and Development, 38*(3), 394-402. <https://doi.org/https://doi.org/10.1111/j.1365-2214.2011.01275.x>

Visagie, M., Coetzee, D., & Pienaar, A. E. (2017). Activity preferences of 9-to 10-year-old girls and the relationship between object control skills and physical activity levels: The nw-child study. *South African Journal for Research in Sport, Physical Education and Recreation, 39*(1), 199-217.

Visser, E. L., Mazzoli, E., Hinkley, T., Lander, N. J., Utesch, T., & Barnett, L. M. (2020). Are children with higher self-reported wellbeing and perceived motor competence more physically active? A longitudinal study. *Journal of Science and Medicine in Sport, 23*(3), 270-275. <https://doi.org/https://doi.org/10.1016/j.jsams.2019.09.005>

Wang, C. K. J., Chia, Y. H. M., Quek, J. J., & Liu, W. C. (2006). Patterns of physical activity, sedentary behaviors, and psychological determinants of physical activity among singaporean school children. *International Journal of Sport and Exercise Psychology, 4*(3), 227-249. <https://doi.org/10.1080/1612197X.2006.9671797>

Webb, O. J., Benjamin, C. C., Gammon, C., McKee, H. C., & Biddle, S. J. H. (2013). Physical activity, sedentary behaviour and physical self-perceptions in adolescent girls: A mediation analysis. *Mental Health and Physical Activity, 6*(1), 24-29. <https://doi.org/https://doi.org/10.1016/j.mhpa.2012.08.005>

Weedon, B. D., Esser, P., Collett, J., Izadi, H., Joshi, S., Meaney, A., . . . Dawes, H. (2023). The relationship between motor competence physical activity cardiorespiratory fitness and bmi in uk adolescents. *Research Quarterly for Exercise and Sport*, 1-7. <https://doi.org/10.1080/02701367.2023.2265442>

Welk, G. J., & Schaben, J. A. (2004). Psychosocial correlates of physical activity in children-a study of relationships when children have similar opportunities to be active. *Measurement in Physical Education and Exercise Science, 8*(2), 63-81. <https://doi.org/10.1207/s15327841mpee0802_2>

Wrotniak, B. H., Epstein, L. H., Dorn, J. M., Jones, K. E., & Kondilis, V. A. (2006). The relationship between motor proficiency and physical activity in children. *Pediatrics, 118*(6), e1758-e1765. <https://doi.org/10.1542/peds.2006-0742>

Yli-Piipari, S., Gråstén, A., Huhtiniemi, M., Salin, K., & Jaakkola, T. (2021). One-year stability of physical education-centered physical literacy indicators on objectively measured physical activity. *European Physical Education Review, 28*(2), 361-379. <https://doi.org/10.1177/1356336X211046302>

Zeng, N., Johnson, S. L., Boles, R. E., & Bellows, L. L. (2019). Social-ecological correlates of fundamental movement skills in young children. *Journal of Sport and Health Science, 8*(2), 122-129. <https://doi.org/https://doi.org/10.1016/j.jshs.2019.01.001>

Zhang, T., Lee, J., Chu, T. L., Chen, C., & Gu, X. (2020). Accessing physical activity and health disparities among underserved hispanic children: The role of actual and perceived motor competence. *International Journal of Environmental Research and Public Health, 17*(9). doi:10.3390/ijerph17093013

Zhang, T., Thomas, K., & Weiller, K. (2015). Predicting physical activity in 10-12 year old children: A social ecological approach. *Journal of Teaching in Physical Education, 34*(3), 517-536. <https://doi.org/10.1123/jtpe.2013-0195>

Ziviani, J., MacDonald, D., Jenkins, D., Rodger, S., Batch, J., & Cerin, E. (2006). Physical activity of young children. *OTJR: Occupational Therapy Journal of Research, 26*(1), 4-14. <https://doi.org/10.1177/153944920602600102>
