# Supplementary material for: Children's and Adolescents’ Actual Motor Competence, Perceived Physical Competence and Physical Activity: A Structural Equation Modelling Meta-Analysis
Source: Sports Med. 2025 May 6;55(8):1923–36. doi: 10.1007/s40279-025-02233-2 (PMC12460483; doi:10.1007/s40279-025-02233-2)
Supplement: Supplementary file 3 — Supplementary file3 (DOCX 170 KB) [file 40279_2025_2233_MOESM3_ESM.docx]

Table C1 – Study quality assessment

| Study | 1 | 2 | 3 | 4 | 5 | 6 |
| --- | --- | --- | --- | --- | --- | --- |
| (Aadland et al., 2017) | Y | Y | N | N | NA | N |
| (Aalizadeh, Mohamadzadeh, & Hosseini, 2014) | Y | Y | Y | N | NA | Y |
| (Adank, Van Kann, Remmers, Kremers, & Vos, 2021) | UC | Y | Y | NA | NA | Y |
| (Afthentopoulou, Venetsanou, Zounhia, & Petrogiannis, 2018) | N | Y | Y | N | Y | Y |
| (Amraei & Azadian, 2021) | Y | Y | Y | N | Y | Y |
| (Anderson, Mâsse, Zhang, Coleman, & Chang, 2009) | UC | Y | NA | NA | N | N |
| (Bai, Chen, Vazou, Welk, & Schaben, 2015) | UC | N | NA | NA | Y | Y |
| (Baker & Davison, 2011) | N | Y | NA | NA | Y | Y |
| (Balaban, 2018) | N | Y | Y | N | NA | Y |
| (Bardid et al., 2016) | N | Y | Y | NA | Y | Y |
| (Barnett, Morgan, Van Beurden, Ball, & Lubans, 2011) | N | N | Y | N | Y | Y |
| (Barnett, Morgan, van Beurden, & Beard, 2008) | N | Y | Y | Y | Y | Y |
| (Barnett, Ridgers, Hesketh, & Salmon, 2017) | N | Y | NA | NA | Y | N |
| (Barnett, Ridgers, & Salmon, 2015) | Y | Y | Y | Y | Y | Y |
| (Barnett, Salmon, & Hesketh, 2016) | Y | Y | Y | Y | Y | N |
| (Barnett et al., 2019) | UC | Y | Y | Y | N | Y |
| (Barnett, Hinkley, Okely, & Salmon, 2013) | UC | Y | Y | Y | NA | Y |
| (Bergqvist-Norén, Hagman, Xiu, Marcus, & Hagströmer, 2022) | UC | Y | Y | NA | NA | N |
| (Bernal, Lhuisset, Bru, Fabre, & Bois, 2024) | N | Y | Y | NA | NA | UC |
| (Bezerra et al., 2021) | N | Y | Y | Y | NA | Y |
| (Blomqvist, Mononen, Tolvanen, & Konttinen, 2019) | UC | N | Y | NA | NA | N |
| (Bois, Sarrazin, Brustad, Trouilloud, & Cury, 2005) | UC | Y | NA | NA | Y | UC |
| (Bolger et al., 2019) | UC | N | Y | Y | NA | UC |
| (Bolger et al., 2018) | UC | Y | Y | Y | Y | Y |
| (Boucher, Doescher, & Sugawara, 1993) | N | Y | Y | N | Y | Y |
| (Breau et al., 2021) | UC | Y | N | N | NA | N |
| (Bremer et al., 2020) | UC | Y | Y | Y | NA | N |
| (Brian et al., 2018) | N | Y | Y | N | Y | Y |
| (Brian, Taunton, Shortt, Pennell, & Sacko, 2019) | UC | Y | Y | N | Y | N |
| (Bürgi et al., 2011) | Y | Y | N | NA | NA | N |
| (Burns et al., 2022) | N | Y | Y | Y | NA | Y |
| (Burns & Fu, 2018) | N | Y | Y | Y | N | Y |
| (Burns, Brusseau, & Hannon, 2017) | N | Y | Y | Y | NA | Y |
| (Capio & Eguia, 2021) | N | Y | Y | N | Y | Y |
| (Capio, Sit, Eguia, & Abernethy, 2014) | UC | Y | Y | Y | NA | UC |
| (Carballo-Fazanes, Díaz-Pereira, Fernández-Villarino, Abelairas-Gómez, & Rey, 2023) | N | Y | Y | NA | NA | Y |
| (Carcamo-Oyarzun, Estevan, & Herrmann, 2020) | N | Y | Y | N | Y | UC |
| (Carvalho et al., 2021) | N | Y | Y | N | NA | Y |
| (Chan, Ha, Ng, & Lubans, 2019) | UC | Y | Y | Y | Y | N |
| (Chaves et al., 2015) | Y | Y | Y | NA | NA | UC |
| (Cliff, Okely, Smith, & McKeen, 2009) | Y | Y | Y | N | NA | N |
| (Cohen, Morgan, Plotnikoff, Callister, & Lubans, 2014) | UC | Y | Y | Y | NA | N |
| (Coker & Herrick, 2021) | N | Y | Y | Y | Y | UC |
| (Cook et al., 2019) | UC | Y | Y | N | NA | Y |
| (Coppens et al., 2021) | UC | Y | Y | N | Y | Y |
| (Craft, Pfeiffer, & Pivarnik, 2003) | N | Y | NA | NA | Y | Y |
| (Craike et al., 2014) | Y | Y | NA | NA | Y | Y |
| (Crane, Foley, Naylor, & Temple, 2017) | UC | Y | Y | N | Y | N |
| (Crane, Foley, & Temple, 2023) | UC | Y | Y | N | Y | N |
| (Crocker, Eklund, & Kowalski, 2000) | UC | Y | NA | NA | Y | UC |
| (Cumming et al., 2011) | UC | Y | NA | NA | Y | UC |
| (Davison, Schmalz, & Downs, 2010) | N | Y | NA | NA | Y | N |
| (de Bruijn & van der Wilt, 2023) | UC | Y | NA | NA | Y | Y |
| (De Meester et al., 2016a) | Y | Y | Y | NA | Y | UC |
| (De Meester et al., 2016b) | N | Y | Y | N | Y | UC |
| (de Oliveira Martins, Flôres, Valentini, & Copetti, 2023) | Y | Y | Y | N | Y | UC |
| (de Witte et al., 2022) | UC | Y | Y | NA | Y | N |
| (den Uil, Janssen, Busch, Kat, & Scholte, 2023) | UC | Y | Y | N | Y | UC |
| (D'Hondt et al., 2014) | Y | Y | Y | NA | NA | N |
| (Dishman et al., 2006) | UC | Y | NA | NA | Y | UC |
| (DuBose, Gross McMillan, Wood, & Sisson, 2018) | N | Y | Y | N | NA | N |
| (Duncan, Jones, O’Brien, Barnett, & Eyre, 2018) | N | Y | Y | Y | Y | UC |
| (Duncan & Stanley, 2012) | N | Y | Y | N | NA | Y |
| (Eberline, Judge, Walsh, & Hensley, 2018) | N | Y | NA | NA | Y | Y |
| (Emadirad, Temple, Field, Naylor, & Temple, 2021) | N | Y | Y | N | NA | Y |
| (Ensrud-Skraastad & Haga, 2020) | N | Y | Y | NA | Y | Y |
| (Estevan et al., 2022) | N | Y | Y | Y | Y | Y |
| (Estevan et al., 2021) | N | Y | Y | NA | Y | N |
| (Estevan & Barnett, 2018) | N | Y | Y | N | Y | Y |
| (Famelia, Tsuda, Bakhtiar, & Goodway, 2018) | N | Y | Y | Y | Y | UC |
| (Farmer, Belton, & O’Brien, 2017) | N | Y | Y | Y | Y | Y |
| (Feitoza, Santos, Barnett, & Cattuzzo, 2022) | N | Y | Y | Y | Y | Y |
| (Field & Temple, 2017) | UC | Y | Y | N | NA | Y |
| (Fisher et al., 2005) | UC | Y | Y | N | NA | Y |
| (Foulkes et al., 2022) | UC | Y | Y | Y | NA | N |
| (Foweather et al., 2015) | Y | Y | Y | Y | NA | Y |
| (Fu & Burns, 2018) | N | Y | Y | Y | NA | Y |
| (Gilson, Cooke, & Mahoney, 2005) | N | Y | NA | NA | Y | Y |
| (Giuriato et al., 2022) | UC | N | Y | NA | NA | UC |
| (Goodway & Rudisill, 1997) | UC | Y | Y | N | Y | Y |
| (Gråstén, Kolunsarka, Huhtiniemi, & Jaakkola, 2022) | Y | Y | N | NA | Y | Y |
| (Gråstén, Huhtiniemi, & Jaakkola, 2022) | Y | Y | N | NA | Y | Y |
| (Gu, 2016) | UC | Y | Y | N | NA | Y |
| (Gu, Chen, & Zhang, 2019) | UC | Y | Y | Y | NA | Y |
| (Gu et al., 2021) | UC | Y | Y | Y | NA | Y |
| (Gu, Thomas, & Chen, 2017) | UC | Y | Y | N | N | Y |
| (Guan, Xiang, Land, & Hamilton, 2023) | UC | Y | NA | NA | N | Y |
| (Guo, Schenkelberg, O’Neill, Dowda, & Pate, 2018) | UC | Y | Y | N | N | Y |
| (Haapala et al., 2023) | UC | Y | N | NA | NA | Y |
| (Hall, Eyre, Oxford, & Duncan, 2018) | N | Y | Y | Y | NA | Y |
| (Hall, Eyre, Oxford, & Duncan, 2019) | N | Y | Y | Y | Y | N |
| (Han, Li, Meng, Li, & Tong, 2022) | UC | Y | Y | N | NA | Y |
| (Hands, Larkin, Parker, Straker, & Perry, 2009) | Y | Y | Y | N | NA | Y |
| (Hardman, Wanderley, Oliveira, & Barros, 2017) | Y | Y | Y | N | NA | N |
| (Haugen, Ommundsen, & Seiler, 2013) | UC | Y | NA | NA | Y | Y |
| (Haugland, Nilsen, Okely, Aadland, & Aadland, 2023) | Y | Y | Y | Y | NA | N |
| (He, Ng, Cairney, Bedard, & Ha, 2021) | UC | Y | N | N | N | Y |
| (Hikihara et al., 2022) | UC | Y | N | N | NA | N |
| (Hill et al., 2022) | Y | Y | N | Y | Y | Y |
| (Houwen, Hartman, & Visscher, 2009) | N | Y | Y | N | NA | Y |
| (Hulteen, True, & Pfeiffer, 2020) | UC | Y | Y | N | NA | N |
| (Hulteen et al., 2018) | UC | Y | Y | N | NA | Y |
| (Humble, Yu, & Brown, 2024) | N | Y | Y | N | Y | Y |
| (Hume et al., 2008) | N | Y | N | N | NA | N |
| (Huotari, Heikinaro-Johansson, Watt, & Jaakkola, 2018) | Y | Y | N | NA | NA | Y |
| (Iivonen et al., 2013) | Y | Y | N | NA | NA | N |
| (Jaakkola et al., 2019c) | UC | Y | N | NA | NA | Y |
| (Jaakkola, Yli-Piipari, Huotari, Watt, & Liukkonen, 2016) | N | Y | Y | NA | NA | N |
| (Jaakkola et al., 2019a) | Y | Y | N | NA | NA | N |
| (Jaakkola et al., 2019b) | N | Y | Y | NA | Y | Y |
| (Jaakkola & Washington, 2013) | UC | Y | N | NA | NA | Y |
| (Jarvis et al., 2018) | UC | Y | Y | N | Y | Y |
| (Jekauc, Wagner, Herrmann, Hegazy, & Woll, 2017) | Y | Y | N | NA | Y | N |
| (Johnson, Wadsworth, Rudisill, Irwin, & Bridges, 2022) | N | Y | N | Y | Y | N |
| (Kaioglou, Dania, Kambas, & Venetsanou, 2023) | N | Y | Y | Y | NA | Y |
| (Kalaja, Jaakkola, Liukkonen, & Watt, 2010) | UC | Y | N | NA | Y | Y |
| (Kambas et al., 2012) | Y | Y | Y | NA | NA | Y |
| (Karuc et al., 2020) | Y | Y | Y | N | NA | Y |
| (Kavanagh, Issartel, & Moran, 2019) | UC | Y | Y | N | Y | N |
| (Khodaverdi, Bahram, & Robinson, 2015) | UC | Y | Y | N | Y | Y |
| (Khodaverdi, Bahram, Khalaji, & Kazemnejad, 2013) | UC | Y | Y | N | Y | Y |
| (King-Dowling, Proudfoot, Cairney, & Timmons, 2020) | UC | Y | Y | NA | NA | Y |
| (Kolunsarka, Gråstén, Huhtiniemi, & Jaakkola, 2021) | Y | Y | N | NA | NA | N |
| (Kolunsarka, Gråstén, Stodden, Huhtiniemi, & Jaakkola, 2023) | Y | Y | N | NA | NA | N |
| (Lalor, Brown, & Murdolo, 2016) | N | Y | Y | NA | Y | Y |
| (Larouche, Boyer, Tremblay, & Longmuir, 2013) | N | Y | Y | N | NA | N |
| (Larsen, Kristensen, Junge, Rexen, & Wedderkopp, 2015) | N | Y | N | NA | NA | N |
| (Laukkanen, Pesola, Havu, Sääkslahti, & Finni, 2014) | UC | Y | N | N | NA | Y |
| (Laukkanen, Niemistö, Aunola, Barnett, & Sääkslahti, 2023) | UC | Y | NA | NA | Y | Y |
| (Laukkanen, Pesola, Finni, & Sääkslahti, 2017) | UC | Y | N | NA | NA | N |
| (LeGear et al., 2012) | UC | Y | Y | N | Y | Y |
| (Lin, Cherng, & Chen, 2017) | N | Y | Y | Y | NA | Y |
| (Liong, Ridgers, & Barnett, 2015) | UC | Y | Y | N | Y | Y |
| (Lohbeck, von Keitz, Hohmann, & Daseking, 2021) | UC | Y | N | NA | Y | N |
| (Lopes et al., 2019) | N | Y | Y | N | NA | N |
| (Lopes, Saraiva, Gonçalves, & Rodrigues, 2018) | N | Y | Y | Y | Y | Y |
| (Lopes & Rodrigues, 2021) | N | Y | Y | N | NA | Y |
| (Lopes, Barnett, & Rodrigues, 2016) | N | Y | Y | N | Y | Y |
| (Loucaides, Chedzoy, Bennett, & Walshe, 2004) | N | N | NA | NA | N | UC |
| (Ma & Luo, 2023) | N | Y | Y | N | NA | Y |
| (Matarma et al., 2018) | N | Y | Y | N | NA | N |
| (McGrane, Belton, Powell, & Issartel, 2017) | N | Y | Y | Y | Y | N |
| (McGrane, Powell, Belton, & Issartel, 2018) | N | Y | Y | Y | NA | Y |
| (McIntyre, Parker, Chivers, & Hands, 2018) | N | Y | N | N | Y | Y |
| (Melby et al., 2021) | UC | Y | Y | NA | NA | N |
| (Menescardi et al., 2023) | N | Y | Y | Y | Y | N |
| (Menescardi & Estevan, 2021) | N | Y | Y | Y | Y | Y |
| (Miller, Eather, Duncan, & Lubans, 2019) | N | Y | Y | Y | NA | N |
| (Monacis, Trecroci, Invernizzi, & Colella, 2022) | Y | Y | NA | NA | N | Y |
| (Morano, Colella, Robazza, Bortoli, & Capranica, 2011) | N | Y | N | N | Y | Y |
| (Morano, Bortoli, Ruiz, Campanozzi, & Robazza, 2020) | N | Y | Y | N | Y | Y |
| (Morgan, Okely, Cliff, Jones, & Baur, 2008) | N | Y | Y | N | Y | Y |
| (Morrison, Cairney, Eisenmann, Pfeiffer, & Gould, 2018) | N | Y | Y | Y | Y | Y |
| (Nicolai Ré et al., 2020) | N | Y | Y | N | NA | UC |
| (Niemistö et al., 2019) | Y | Y | Y | Y | Y | UC |
| (Nilsen et al., 2020a) | UC | Y | UC | Y | NA | Y |
| (Nilsen et al., 2020b) | Y | Y | Y | Y | NA | Y |
| (Noonan, Boddy, Knowles, & Fairclough, 2018) | N | Y | NA | NA | N | N |
| (Norman, Geer, & Looper, 2019) | N | Y | Y | N | NA | Y |
| (O’ Brien, Belton, & Issartel, 2016) | N | Y | Y | N | NA | N |
| (Paxton, Estabrooks, & Dzewaltowski, 2004) | N | Y | NA | NA | Y | Y |
| (Peers, Issartel, Behan, O'Connor, & Belton, 2020) | UC | Y | Y | Y | Y | Y |
| (Pereira et al., 2020) | UC | Y | Y | N | NA | N |
| (Pérez & Sanz, 2005) | N | Y | Y | N | Y | Y |
| (Pesce, Masci, Marchetti, Vannozzi, & Schmidt, 2018) | Y | Y | Y | N | Y | Y |
| (Queiroz et al., 2020) | Y | Y | Y | Y | NA | N |
| (Raudsepp, Liblik, & Hannus, 2002) | N | Y | NA | NA | Y | N |
| (Raudsepp & Päll, 2006) | Y | Y | N | Y | NA | UC |
| (Reed, Metzker, & Phillips, 2004) | N | Y | UC | N | NA | Y |
| (Reyes et al., 2019) | N | Y | Y | NA | NA | Y |
| (Robinson & Palmer, 2021) | N | Y | Y | N | Y | Y |
| (Robinson, 2011) | Y | Y | Y | N | Y | Y |
| (Robinson, Wadsworth, & Peoples, 2012) | N | Y | Y | N | Y | N |
| (Rogers, Barnett, & Lander, 2018) | N | Y | UC | Y | Y | N |
| (Rudisill, Mahar, & Meaney, 1993) | N | Y | N | N | Y | N |
| (Ryu, Lee, Liu, McDonough, & Gao, 2021a) | N | Y | Y | N | Y | Y |
| (Ryu et al., 2021b) | N | Y | Y | N | Y | Y |
| (Sabiston & Crocker, 2008) | UC | Y | NA | NA | N | Y |
| (Sallen, Andrä, Ludyga, Mücke, & Herrmann, 2020) | N | Y | Y | N | Y | Y |
| (Sallis, Alcaraz, McKenzie, & Hovell, 1999) | UC | Y | NA | NA | Y | N |
| (Santos et al., 2018) | N | Y | Y | N | NA | Y |
| (Schmutz et al., 2020) | Y | Y | Y | N | NA | UC |
| (Seabra et al., 2013) | N | Y | NA | NA | Y | Y |
| (Shull et al., 2022) | N | Y | Y | N | NA | Y |
| (Silva-Santos et al., 2021) | UC | Y | Y | NA | NA | N |
| (Silva-Santos, Santos, Duncan, Vale, & Mota, 2019) | UC | UC | Y | N | NA | UC |
| (Slykerman, Ridgers, Stevenson, & Barnett, 2016) | N | Y | Y | Y | Y | Y |
| (Smith, Fazeli, Wilkinson, & Clark, 2021) | N | Y | Y | N | NA | UC |
| (Spessato, Gabbard, Robinson, & Valentini, 2013) | UC | Y | Y | N | Y | UC |
| (Spessato, Gabbard, & Valentini, 2013) | UC | Y | Y | Y | NA | UC |
| (Stein, Fisher, Berkey, & Colditz, 2007) | Y | Y | NA | NA | Y | N |
| (Strotmeyer, Herrmann, & Kehne, 2022) | N | Y | Y | NA | Y | Y |
| (Sung, Loh, & Lin, 2021) | N | Y | Y | Y | N | UC |
| (Syväoja et al., 2021) | UC | Y | N | N | NA | UC |
| (Telford, Telford, Olive, Cochrane, & Davey, 2016) | Y | Y | Y | NA | N | Y |
| (Temple, Crane, Brown, Williams, & Bell, 2016) | UC | Y | Y | N | NA | Y |
| (Tietjens et al., 2020) | N | Y | Y | N | Y | Y |
| (Tietjens et al., 2018) | N | Y | Y | N | N | Y |
| (Toftegaard-Stoeckel, Groenfeldt, & Andersen, 2010) | N | Y | Y | NA | Y | Y |
| (True, Brian, Goodway, & Stodden, 2017) | UC | Y | Y | Y | Y | Y |
| (Tsuda, Goodway, Famelia, & Brian, 2020) | UC | Y | Y | Y | Y | Y |
| (Valentini, Souza, Souza, & Nobre, 2023) | UC | Y | Y | Y | Y | Y |
| (Valentini, Nobre, de Souza, & Duncan, 2020) | N | Y | Y | Y | Y | UC |
| (van Niekerk, du Toit, & Pienaar, 2016) | Y | Y | Y | N | NA | Y |
| (Vedul-Kjelsås, Sigmundsson, Stensdotter, & Haga, 2012) | UC | Y | Y | N | Y | Y |
| (Veldman et al., 2018) | UC | Y | Y | N | NA | Y |
| (Visagie, Coetzee, & Pienaar, 2017) | Y | Y | Y | N | NA | Y |
| (Visser et al., 2020) | N | Y | NA | NA | Y | Y |
| (Wang, Chia, Quek, & Liu, 2006) | UC | Y | NA | NA | Y | Y |
| (Webb, Benjamin, Gammon, McKee, & Biddle, 2013) | UC | Y | NA | NA | Y | Y |
| (Weedon et al., 2023) | N | Y | Y | N | NA | N |
| (Welk & Schaben, 2004) | N | Y | NA | NA | Y | Y |
| (Wrotniak, Epstein, Dorn, Jones, & Kondilis, 2006) | N | Y | Y | NA | NA | Y |
| (Yli-Piipari, Gråstén, Huhtiniemi, Salin, & Jaakkola, 2021) | N | Y | Y | NA | NA | N |
| (Zeng, Johnson, Boles, & Bellows, 2019) | UC | Y | Y | NA | Y | Y |
| (Zhang, Lee, Chu, Chen, & Gu, 2020) | UC | Y | Y | Y | N | Y |
| (Zhang, Thomas, & Weiller, 2015) | UC | Y | Y | N | Y | UC |
| (Ziviani et al., 2006) | Y | Y | Y | N | Y | N |

**Criteria:**

1. Does the study use a representative sample? (Y = Random sampling / selection of target population used; N = Convenience sampling used, UC = Sampling method not stated or unclear)

2. Does the study adequately describe participant sampling procedures and inclusion criteria? (Y = Inclusion / exclusion criteria clearly described AND/OR authors clearly outline demographic information of participants [at a minimum age and sex/gender]; N = Participants inclusion criteria AND participant demographic information are not presented; UC = Inclusion criteria / demographic information not clearly described)

3. Does the study clearly outline the actual motor competence assessment(s) used and are they valid and reliable? (Y = Actual motor competence assessment is clearly outlined, source is referenced, AND validity of the assessment for the target population is clearly stated within the text, OR previous validation study is referenced. Full details and validation provided for each measure AND reports at least one ‘acceptable’ reliability statistic [e.g., Cronbach alphas ≥ 0.70 or test–retest reliability intraclass correlation coefficient [ICC] ≥ 0.60, Brown et al. [201] OR previous reliability study is referenced]; N = Actual motor competence assessment not outlined or referenced, OR validity of the assessment for the target population is not clearly stated within the text AND previous validation study is not referenced OR reliability of the assessment for the target population is not clearly stated within the text AND previous reliability study is not referenced. Single measure(s) are not outlined, and validation data not provided, or part measure(s) are used; UC = Unclear if valid or reliable measure used due to inadequate description; NA = actual motor competence not assessed)

4. Does the study provide acceptable inter-rater reliability information for the AMC assessment(s) used? (Y = Acceptable inter-rater reliability statistic clearly highlighted (an ICC ≥ 0.60); N = Actual motor competence assessed by a single assessor OR reliability data not reported OR reliability statistic was not acceptable [i.e. ICC < 0.60]; UC = Inadequate description so unclear if reliable measure was use; NA = Actual motor competence not assessed OR actual motor competence assessed using product-related outcomes [e.g., time balancing on one leg, get-up-an-go speed, jumping distance])

5. Does the study clearly outline the perceived physical competence assessments(s) used and are they valid and reliable? (Y = Perceived physical competence assessment is clearly outlined, source is referenced, AND validity of the assessment for the target population is clearly stated within the text, OR previous validation study is referenced. Full details and validation provided for each measure AND reports at least one ‘acceptable’ reliability statistic (e.g., Cronbach alphas ≥ 0.70 or test–retest reliability intraclass correlation coefficient [ICC] ≥ 0.60, Brown et al. [201] OR previous reliability study is referenced; N = Perceived motor competence assessment not outlined or referenced, OR validity of the assessment for the target population is not clearly stated within the text AND previous validation study is not referenced OR reliability of the assessment for the target population is not clearly stated within the text AND previous reliability study is not referenced. Single measure(s) are not outlined, and validation data not provided, or part measure(s) are used; UC = Unclear if valid or reliable measure used due to inadequate description; NA = PMC not assessed)

6. Of those who consented to the study, did an adequate proportion have complete data for all assessment(s)? (Y = Clearly identifiable from the text or tables that no fewer than 80% (cross-sectional studies) or 70% (longitudinal studies) of participants completed all measures; N = < 80% (cross-sectional studies) or < 70% (longitudinal studies) of participants completed all measures; UC = Inadequate description so unclear what percentage of total number of participants completed each assessment)

**References**

Aadland, K. N., Moe, V. F., Aadland, E., Anderssen, S. A., Resaland, G. K., & Ommundsen, Y. (2017). Relationships between physical activity, sedentary time, aerobic fitness, motor skills and executive function and academic performance in children. *Mental Health and Physical Activity, 12*, 10-18. <https://doi.org/:https://doi.org/10.1016/j.mhpa.2017.01.001>

Aalizadeh, B., Mohamadzadeh, H., & Hosseini, F. S. (2014). Fundamental Movement Skills among Iranian Primary School Children. *Journal of Family and Reproductive Health, 8*(4), 155-159.

Adank, A. M., Van Kann, D. H. H., Remmers, T., Kremers, S. P. J., & Vos, S. B. (2021). Longitudinal Perspectives on Children’s Physical Activity Patterns: “Do Physical Education–Related Factors Matter?”. *Journal of Physical Activity and Health, 18*(10), 1199-1206. <https://doi.org/:10.1123/jpah.2020-0859>

Afthentopoulou, A.-E., Venetsanou, F., Zounhia, A., & Petrogiannis, K. (2018). Physical activity, motor competence, and perceived physical competence: what is their relationship in children aged 6–9 years? *Human Movement, 19*(1), 51-56. <https://doi.org/:10.5114/hm.2018.73612>

Amraei, M., & Azadian, E. (2021). Motor Competence–Related Age and Living Environment in Girls: A Cross-Sectional Study. *Journal of Motor Learning and Development, 9*(3), 470-482. <https://doi.org/:10.1123/jmld.2021-0010>

Anderson, C. B., Mâsse, L. C., Zhang, H., Coleman, K. J., & Chang, S. (2009). Contribution of athletic identity to child and adolescent physical activity. *American Journal of Preventive Medicine, 37*(3), 220-226. <https://doi.org/:10.1016/j.amepre.2009.05.017>

Bai, Y., Chen, S., Vazou, S., Welk, G. J., & Schaben, J. (2015). Mediated Effects of Perceived Competence on Youth Physical Activity and Sedentary Behavior. *Research Quarterly for Exercise and Sport, 86*(4), 406-413. <https://doi.org/:10.1080/02701367.2015.1087639>

Baker, B. L., & Davison, K. K. (2011). I Know I Can: A Longitudinal Examination of Precursors and Outcomes of Perceived Athletic Competence Among Adolescent Girls. *Journal of Physical Activity and Health, 8*(2), 192-199. <https://doi.org/:10.1123/jpah.8.2.192>

Balaban, V. (2018). The Relationship between Objectively Measured Physical Activity and Fundamental Motor Skills in 8 to 11 Years Old Children from the Czech Republic. *Montenegrin Journal of Sports Science and Medicine, 7*(2). <https://doi.org/:10.26773/mjssm.180902>

Bardid, F., De Meester, A., Tallir, I., Cardon, G., Lenoir, M., & Haerens, L. (2016). Configurations of actual and perceived motor competence among children: Associations with motivation for sports and global self-worth. *Human Movement Science, 50*, 1-9. <https://doi.org/:10.1016/j.humov.2016.09.001>

Barnett, L., Hinkley, T., Okely, A. D., & Salmon, J. (2013). Child, family and environmental correlates of children's motor skill proficiency. *Journal of Science and Medicine in Sport, 16*(4), 332-336. <https://doi.org/:10.1016/j.jsams.2012.08.011>

Barnett, L. M., Morgan, P. J., Van Beurden, E., Ball, K., & Lubans, D. R. (2011). A reverse pathway? Actual and perceived skill proficiency and physical activity. *Medicine & Science in Sports & Exercise, 43*(5), 898-904. <https://doi.org/:10.1249/MSS.0b013e3181fdfadd>

Barnett, L. M., Morgan, P. J., van Beurden, E., & Beard, J. R. (2008). Perceived sports competence mediates the relationship between childhood motor skill proficiency and adolescent physical activity and fitness: a longitudinal assessment. *International Journal of Behavioral Nutrition and Physical Activity, 5*(1), 40. <https://doi.org/:10.1186/1479-5868-5-40>

Barnett, L. M., Ridgers, N. D., Hesketh, K., & Salmon, J. (2017). Setting them up for lifetime activity: Play competence perceptions and physical activity in young children. *Journal of Science and Medicine in Sport, 20*(9), 856-860. <https://doi.org/:10.1016/j.jsams.2017.03.003>

Barnett, L. M., Ridgers, N. D., & Salmon, J. (2015). Associations between young children's perceived and actual ball skill competence and physical activity. *Journal of Science and Medicine in Sport, 18*(2), 167-171. <https://doi.org/:10.1016/j.jsams.2014.03.001>

Barnett, L. M., Salmon, J., & Hesketh, K. D. (2016). More active pre-school children have better motor competence at school starting age: an observational cohort study. *BMC Public Health, 16*(1), 1068. <https://doi.org/:10.1186/s12889-016-3742-1>

Barnett, L. M., Telford, R. M., Strugnell, C., Rudd, J., Olive, L. S., & Telford, R. D. (2019). Impact of cultural background on fundamental movement skill and its correlates. *Journal of Sports Sciences, 37*(5), 492-499. <https://doi.org/:10.1080/02640414.2018.1508399>

Bergqvist-Norén, L., Hagman, E., Xiu, L., Marcus, C., & Hagströmer, M. (2022). Physical activity in early childhood: a five-year longitudinal analysis of patterns and correlates. *International Journal of Behavioral Nutrition and Physical Activity, 19*(1), 47. <https://doi.org/:10.1186/s12966-022-01289-x>

Bernal, C. M. M., Lhuisset, L., Bru, N., Fabre, N., & Bois, J. (2024). Do physical activity, sedentary time, motor skills and aerobic fitness predict primary school children’s attention? Use of a data mining strategy. *International Journal of Sport and Exercise Psychology*, 1-18. <https://doi.org/:10.1080/1612197X.2023.2239841>

Bezerra, T. A., Bandeira, P. F. R., de Souza Filho, A. N., Clark, C. C. T., Mota, J., Duncan, M. J., & de Lucena Martins, C. M. (2021). A Network Perspective on the Relationship Between Moderate to Vigorous Physical Activity and Fundamental Motor Skills in Early Childhood. *Journal of Physical Activity and Health, 18*(7), 774-781. <https://doi.org/:10.1123/jpah.2020-0218>

Blomqvist, M., Mononen, K., Tolvanen, A., & Konttinen, N. (2019). Objectively assessed vigorous physical activity and motor coordination are associated in 11-year old children. *Scandinavian Journal of Medicine & Science in Sports, 29*(10), 1629-1635. <https://doi.org/:https://doi.org/10.1111/sms.13500>

Bois, J. E., Sarrazin, P. G., Brustad, R. J., Trouilloud, D. O., & Cury, F. (2005). Elementary schoolchildren's perceived competence and physical activity involvement: the influence of parents' role modelling behaviours and perceptions of their child's competence. *Psychology of Sport and Exercise, 6*(4), 381-397. <https://doi.org/:https://doi.org/10.1016/j.psychsport.2004.03.003>

Bolger, L. A., Bolger, L. E., O’Neill, C., Coughlan, E., Lacey, S., O’Brien, W., & Burns, C. (2019). Fundamental Movement Skill Proficiency and Health Among a Cohort of Irish Primary School Children. *Research Quarterly for Exercise and Sport, 90*(1), 24-35. <https://doi.org/:10.1080/02701367.2018.1563271>

Bolger, L. E., Bolger, L. A., O'Neill, C., Coughlan, E., O'Brien, W., Lacey, S., & Burns, C. (2018). Accuracy of Children's Perceived Skill Competence and its Association With Physical Activity. *Journal of Physical Activity and Health*, 1-8. <https://doi.org/:10.1123/jpah.2017-0371>

Boucher, B. H., Doescher, S. M., & Sugawara, A. I. (1993). Preschool children's motor development and self-concept. *Perceptual and Motor Skills, 76*(1), 11-17. <https://doi.org/:10.2466/pms.1993.76.1.11>

Breau, B., Brandes, B., Wright, M. N., Buck, C., Vallis, L. A., & Brandes, M. (2021). Association of Individual Motor Abilities and Accelerometer-Derived Physical Activity Measures in Preschool-Aged Children. *Journal for the Measurement of Physical Behaviour, 4*(3), 227-235. <https://doi.org/:10.1123/jmpb.2020-0065>

Bremer, E., Graham, J. D., Bedard, C., Rodriguez, C., Kriellaars, D., & Cairney, J. (2020). The Association Between PLAYfun and Physical Activity: A Convergent Validation Study. *Research Quarterly for Exercise and Sport, 91*(2), 179-187. <https://doi.org/:10.1080/02701367.2019.1652723>

Brian, A., Bardid, F., Barnett, L. M., Deconinck, F. J. A., Lenoir, M., & Goodway, J. D. (2018). Actual and Perceived Motor Competence Levels of Belgian and United States Preschool Children. *Journal of Motor Learning and Development, 6*(s2), S320-S336. <https://doi.org/:10.1123/jmld.2016-0071>

Brian, A., Taunton, S., Shortt, C., Pennell, A., & Sacko, R. (2019). Predictors of Physical Activity for Preschool Children With and Without Disabilities From Socioeconomically Disadvantaged Settings. *Adapt Phys Activ Q, 36*(1), 77-90. <https://doi.org/:10.1123/apaq.2017-0191>

Bürgi, F., Meyer, U., Granacher, U., Schindler, C., Marques-Vidal, P., Kriemler, S., & Puder, J. J. (2011). Relationship of physical activity with motor skills, aerobic fitness and body fat in preschool children: a cross-sectional and longitudinal study (Ballabeina). *International Journal of Obesity, 35*(7), 937-944. <https://doi.org/:10.1038/ijo.2011.54>

Burns, R., Brusseau, T., & Hannon, J. (2017). Multivariate Associations Among Health-Related Fitness, Physical Activity, and TGMD-3 Test Items in Disadvantaged Children From Low-Income Families. *Perceptual and Motor Skills, 124*(1), 86-104. <https://doi.org/:10.1177/0031512516672118>

Burns, R. D., Bai, Y., Byun, W., Colotti, T. E., Pfledderer, C. D., Kwon, S., & Brusseau, T. A. (2022). Bidirectional relationships of physical activity and gross motor skills before and after summer break: Application of a cross-lagged panel model. *Journal of Sport and Health Science, 11*(2), 244-251. <https://doi.org/:https://doi.org/10.1016/j.jshs.2020.07.001>

Burns, R. D., & Fu, Y. (2018). Testing the Motor Competence and Health-Related Variable Conceptual Model: A Path Analysis. *Journal of Functional Morphology and Kinesiology, 3*(4). <https://doi.org/:10.3390/jfmk3040061>

Capio, C. M., & Eguia, K. F. (2021). Movement skills, perception, and physical activity of young children: A mediation analysis. *Pediatrics International, 63*(4), 442-447. <https://doi.org/:10.1111/ped.14436>

Capio, C. M., Sit, C. H., Eguia, K. F., & Abernethy, B. (2014). Physical activity and movement skills proficiency of young Filipino children. *Pediatrics International, 56*(4), 651-653. <https://doi.org/:10.1111/ped.12436>

Carballo-Fazanes, A., Díaz-Pereira, M. P., Fernández-Villarino, M. A., Abelairas-Gómez, C., & Rey, E. (2023). Physical activity in kindergarten, fundamental movement skills, and screen time in Spanish preschool children. *Psychology in the Schools, 60*(9), 3318-3328. <https://doi.org/:https://doi.org/10.1002/pits.22925>

Carcamo-Oyarzun, J., Estevan, I., & Herrmann, C. (2020). Association between Actual and Perceived Motor Competence in School Children. *International Journal of Environmental Research and Public Health, 17*(10). <https://doi.org/:10.3390/ijerph17103408>

Carvalho, A. S., Bohn, L., Abdalla, P. P., Ramos, N. C., Borges, F. G., Mota, J., & Machado, D. R. L. (2021). The Associations of Objectively Measured Physical Activity, Fundamental Motor Skills and Time in Sedentary Behavior in Children: A Cross-Sectional Study. *Perceptual and Motor Skills, 128*(6), 2507-2526. <https://doi.org/:10.1177/00315125211038731>

Chan, C. H. S., Ha, A. S. C., Ng, J. Y. Y., & Lubans, D. R. (2019). Associations between fundamental movement skill competence, physical activity and psycho-social determinants in Hong Kong Chinese children. *Journal of Sports Sciences, 37*(2), 229-236. <https://doi.org/:10.1080/02640414.2018.1490055>

Chaves, R., Baxter-Jones, A., Gomes, T., Souza, M., Pereira, S., & Maia, J. (2015). Effects of Individual and School-Level Characteristics on a Child's Gross Motor Coordination Development. *International Journal of Environmental Research and Public Health, 12*(8), 8883-8896. <https://doi.org/:10.3390/ijerph120808883>

Cliff, D. P., Okely, A. D., Smith, L. M., & McKeen, K. (2009). Relationships between fundamental movement skills and objectively measured physical activity in preschool children. *Pediatric Exercise Science, 21*(4), 436-449. <https://doi.org/:10.1123/pes.21.4.436>

Cohen, K. E., Morgan, P. J., Plotnikoff, R. C., Callister, R., & Lubans, D. R. (2014). Fundamental movement skills and physical activity among children living in low-income communities: a cross-sectional study. *International Journal of Behavioral Nutrition and Physical Activity, 11*(1), 49. <https://doi.org/:10.1186/1479-5868-11-49>

Coker, C. A., & Herrick, B. (2021). Functional Movement Proficiency’s Association to Actual and Perceived Motor Competence. *Journal of Motor Learning and Development, 9*(1), 28-37. <https://doi.org/:10.1123/jmld.2020-0002>

Cook, C. J., Howard, S. J., Scerif, G., Twine, R., Kahn, K., Norris, S. A., & Draper, C. E. (2019). Associations of physical activity and gross motor skills with executive function in preschool children from low-income South African settings. *Developmental Science, 22*(5), e12820. <https://doi.org/:10.1111/desc.12820>

Coppens, E., De Meester, A., Deconinck, F. J. A., De Martelaer, K., Haerens, L., Bardid, F., . . . D'Hondt, E. (2021). Differences in Weight Status and Autonomous Motivation towards Sports among Children with Various Profiles of Motor Competence and Organized Sports Participation. *Children (Basel), 8*(2). <https://doi.org/:10.3390/children8020156>

Craft, L. L., Pfeiffer, K. A., & Pivarnik, J. M. (2003). Predictors of Physical Competence in Adolescent Girls. *Journal of Youth and Adolescence, 32*(6), 431-438. <https://doi.org/:10.1023/A:1025986318306>

Craike, M. J., Polman, R., Eime, R., Symons, C., Harvey, J., & Payne, W. (2014). Associations between behavior regulation, competence, physical activity, and health for adolescent females. *Journal of Physical Activity and Health, 11*(2), 410-418. <https://doi.org/:10.1123/jpah.2012-0070>

Crane, J. R., Foley, J. T., Naylor, P.-J., & Temple, V. A. (2017). Longitudinal Change in the Relationship between Fundamental Motor Skills and Perceived Competence: Kindergarten to Grade 2. *Sports, 5*(3). doi:10.3390/sports5030059

Crane, J. R., Foley, J. T., & Temple, V. A. (2023). The Influence of Perceptions of Competence on Motor Skills and Physical Activity in Middle Childhood: A Test of Mediation. *International Journal of Environmental Research and Public Health, 20*(9). doi:10.3390/ijerph20095648

Crocker, P. R. E., Eklund, R. C., & Kowalski, K. C. (2000). Children's physical activity and physical self-perceptions. *Journal of Sports Sciences, 18*(6), 383-394. <https://doi.org/:10.1080/02640410050074313>

Cumming, S. P., Standage, M., Loney, T., Gammon, C., Neville, H., Sherar, L. B., & Malina, R. M. (2011). The mediating role of physical self-concept on relations between biological maturity status and physical activity in adolescent females. *Journal of Adolescence, 34*(3), 465-473. <https://doi.org/:https://doi.org/10.1016/j.adolescence.2010.06.006>

D'Hondt, E., Deforche, B., Gentier, I., Verstuyf, J., Vaeyens, R., De Bourdeaudhuij, I., . . . Lenoir, M. (2014). A longitudinal study of gross motor coordination and weight status in children. *Obesity, 22*(6), 1505-1511. <https://doi.org/:https://doi.org/10.1002/oby.20723>

Davison, K. K., Schmalz, D. L., & Downs, D. S. (2010). Hop, Skip … No! Explaining Adolescent Girls’ Disinclination for Physical Activity. *Annals of Behavioral Medicine, 39*(3), 290-302. <https://doi.org/:10.1007/s12160-010-9180-x>

de Bruijn, A. G. M., & van der Wilt, F. (2023). Social Acceptance in Physical Education and the Regular Classroom: Perceived Motor Competency and Frequency and Type of Sports Participation. *Children, 10*(3). doi:10.3390/children10030568

De Meester, A., Maes, J., Stodden, D., Cardon, G., Goodway, J., Lenoir, M., & Haerens, L. (2016a). Identifying profiles of actual and perceived motor competence among adolescents: associations with motivation, physical activity, and sports participation. *Journal of Sports Sciences, 34*(21), 2027-2037. <https://doi.org/:10.1080/02640414.2016.1149608>

De Meester, A., Stodden, D., Brian, A., True, L., Cardon, G., Tallir, I., & Haerens, L. (2016b). Associations among Elementary School Children’s Actual Motor Competence, Perceived Motor Competence, Physical Activity and BMI: A Cross-Sectional Study. *PLOS ONE, 11*(10), e0164600. <https://doi.org/:10.1371/journal.pone.0164600>

de Oliveira Martins, A., Flôres, F., Valentini, N., & Copetti, F. (2023). What do the parents perceive, and how it affects children’s motor competence? An exploratory study in 5 to 11 years old south Brazilian children. *Motricidade, 19*(1), 41-48.

de Witte, A., Hoeboer, J., Coppens, E., Lenoir, M., Platvoet, S., de Niet, M., . . . de Meester, A. (2022). A Variable- and Person-Centered Approach to Further Understand the Relationship Between Actual and Perceived Motor Competence in Children. *Journal of Teaching in Physical Education, 41*(3), 391-400. <https://doi.org/:10.1123/jtpe.2021-0038>

den Uil, A. R., Janssen, M., Busch, V., Kat, I. T., & Scholte, R. H. J. (2023). The relationships between children’s motor competence, physical activity, perceived motor competence, physical fitness and weight status in relation to age. *PLOS ONE, 18*(4), e0278438. <https://doi.org/:10.1371/journal.pone.0278438>

Dishman, R. K., Hales, D. P., Pfeiffer, K. A., Felton, G. A., Saunders, R., Ward, D. S., . . . Pate, R. R. (2006). Physical self-concept and self-esteem mediate cross-sectional relations of physical activity and sport participation with depression symptoms among adolescent girls. *Health Psychology, 25*(3), 396-407. <https://doi.org/:10.1037/0278-6133.25.3.396>

DuBose, K. D., Gross McMillan, A., Wood, A. P., & Sisson, S. B. (2018). Joint Relationship Between Physical Activity, Weight Status, and Motor Skills in Children Aged 3 to 10 Years. *Perceptual and Motor Skills, 125*(3), 478-492. <https://doi.org/:10.1177/0031512518767008>

Duncan, M. J., Jones, V., O’Brien, W., Barnett, L. M., & Eyre, E. L. J. (2018). Self-Perceived and Actual Motor Competence in Young British Children. *Perceptual and Motor Skills, 125*(2), 251-264. <https://doi.org/:10.1177/0031512517752833>

Duncan, M. J., & Stanley, M. (2012). Functional Movement Is Negatively Associated with Weight Status and Positively Associated with Physical Activity in British Primary School Children. *Journal of Obesity, 2012*, 697563. <https://doi.org/:10.1155/2012/697563>

Eberline, A., Judge, L. W., Walsh, A., & Hensley, L. D. (2018). Relationship of Enjoyment, Perceived Competence, and Cardiorespiratory Fitness to Physical Activity Levels of Elementary School Children. *Physical Educator, 75*(3), 394-413. <https://doi.org/:https://doi.org/10.18666/TPE-2018-V75-I3-8161>

Emadirad, E., Temple, B. W. N., Field, S. C., Naylor, P.-J., & Temple, V. A. (2021). Motor Skills and Participation in Middle Childhood: A Direct Path for Boys, a Mediated Path for Girls. *Journal of Physical Activity and Health, 18*(3), 318-324. <https://doi.org/:10.1123/jpah.2020-0296>

Ensrud-Skraastad, O. K., & Haga, M. (2020). Associations between Motor Competence, Physical Self-Perception and Autonomous Motivation for Physical Activity in Children. *Sports, 8*(9). doi:10.3390/sports8090120

Estevan, I., & Barnett, L. M. (2018). Considerations Related to the Definition, Measurement and Analysis of Perceived Motor Competence. *Sports Medicine, 48*(12), 2685-2694. <https://doi.org/:10.1007/s40279-018-0940-2>

Estevan, I., Clark, C., Molina-García, J., Menescardi, C., Barton, V., & Queralt, A. (2022). Longitudinal association of movement behaviour and motor competence in childhood: A structural equation model, compositional, and isotemporal substitution analysis. *Journal of Science and Medicine in Sport, 25*(8), 661-666. <https://doi.org/:https://doi.org/10.1016/j.jsams.2022.05.010>

Estevan, I., Menescardi, C., Castillo, I., Molina-García, J., García-Massó, X., & Barnett, L. M. (2021). Perceived movement skill competence in stability: Validity and reliability of a pictorial scale in early adolescents. *Scandinavian Journal of Medicine & Science in Sports, 31*(5), 1135-1143. <https://doi.org/:https://doi.org/10.1111/sms.13928>

Famelia, R., Tsuda, E., Bakhtiar, S., & Goodway, J. D. (2018). Relationships Among Perceived and Actual Motor Skill Competence and Physical Activity in Indonesian Preschoolers. *Journal of Motor Learning and Development, 6*(s2), S403-S423. <https://doi.org/:10.1123/jmld.2016-0072>

Farmer, O., Belton, S., & O’Brien, W. (2017). The Relationship between Actual Fundamental Motor Skill Proficiency, Perceived Motor Skill Confidence and Competence, and Physical Activity in 8–12-Year-Old Irish Female Youth. *Sports, 5*(4). doi:10.3390/sports5040074

Feitoza, A. H. P., Santos, A. B. D., Barnett, L. M., & Cattuzzo, M. T. (2022). Motor competence, physical activity, and perceived motor competence: A relational systems approach. *Journal of Sports Sciences, 40*(21), 2371-2383. <https://doi.org/:10.1080/02640414.2022.2158268>

Field, S. C., & Temple, V. A. (2017). The Relationship between Fundamental Motor Skill Proficiency and Participation in Organized Sports and Active Recreation in Middle Childhood. *Sports, 5*(2). doi:10.3390/sports5020043

Fisher, A., Reilly, J. J., Kelly, L. A., Montgomery, C., Williamson, A., Paton, J. Y., & Grant, S. (2005). Fundamental Movement Skills and Habitual Physical Activity in Young Children. *Medicine & Science in Sports & Exercise, 37*(4). Retrieved from <https://journals.lww.com/acsm-msse/fulltext/2005/04000/fundamental_movement_skills_and_habitual_physical.23.aspx>

Foulkes, J. D., Knowles, Z., Fairclough, S. J., Stratton, G., O’Dwyer, M. V., & Foweather, L. (2022). Is Foundational Movement Skill Competency Important for Keeping Children Physically Active and at a Healthy Weight? *International Journal of Environmental Research and Public Health, 19*(1). doi:10.3390/ijerph19010105

Foweather, L., Knowles, Z., Ridgers, N. D., O’Dwyer, M. V., Foulkes, J. D., & Stratton, G. (2015). Fundamental movement skills in relation to weekday and weekend physical activity in preschool children. *Journal of Science and Medicine in Sport, 18*(6), 691-696. <https://doi.org/:https://doi.org/10.1016/j.jsams.2014.09.014>

Fu, Y., & Burns, R. D. (2018). Gross Motor Skills and School Day Physical Activity: Mediating Effect of Perceived Competence. *Journal of Motor Learning and Development, 6*(2), 287-300. <https://doi.org/:10.1123/jmld.2017-0043>

Gilson, N., Cooke, C., & Mahoney, C. (2005). Adolescent physical self‐perceptions, sport/exercise and lifestyle physical activity. *Health Education, 105*(6), 437-450.

Giuriato, M., Lovecchio, N., Carnevale Pellino, V., Mieszkowski, J., Kawczyński, A., Nevill, A., & Biino, V. (2022). Gross motor coordination and their relationship with body mass and physical activity level during growth in Children aged 8–11 years old: a longitudinal and allometric approach. *PeerJ, 10*, e13483. <https://doi.org/:10.7717/peerj.13483>

Goodway, J. D., & Rudisill, M. E. (1997). Perceived Physical Competence and Actual Motor Skill Competence of African American Preschool Children. *Adapted Physical Activity Quarterly, 14*(4), 314-326. <https://doi.org/:10.1123/apaq.14.4.314>

Gråstén, A., Huhtiniemi, M., & Jaakkola, T. (2022). School-Age Children’s Actual Motor Competence and Perceived Physical Competence: A 3-Yr Follow-up. *Medicine & Science in Sports & Exercise, 54*(6). Retrieved from <https://journals.lww.com/acsm-msse/fulltext/2022/06000/school_age_children_s_actual_motor_competence_and.15.aspx>

Gråstén, A., Kolunsarka, I., Huhtiniemi, M., & Jaakkola, T. (2022). Developmental associations of actual motor competence and perceived physical competence with health-related fitness in schoolchildren over a four-year follow-up. *Psychology of Sport and Exercise, 63*, 102279. <https://doi.org/:https://doi.org/10.1016/j.psychsport.2022.102279>

Gu, X. (2016). Fundamental motor skill, physical activity, and sedentary behavior in socioeconomically disadvantaged kindergarteners. *Psychology, Health & Medicine, 21*(7), 871-881. <https://doi.org/:10.1080/13548506.2015.1125007>

Gu, X., Chen, S., & Zhang, X. (2019). Young Hispanic and Non-Hispanic Children’s Fundamental Motor Competence and Physical Activity Behaviors. *Journal of Motor Learning and Development, 7*(2), 180-193. <https://doi.org/:10.1123/jmld.2018-0003>

Gu, X., Tamplain, P. M., Chen, W., Zhang, T., Keller, M. J., & Wang, J. (2021). A Mediation Analysis of the Association between Fundamental Motor Skills and Physical Activity during Middle Childhood. *Children, 8*(2). doi:10.3390/children8020064

Gu, X., Thomas, K. T., & Chen, Y.-L. (2017). The Role of Perceived and Actual Motor Competency on Children’s Physical Activity and Cardiorespiratory Fitness During Middle Childhood. *Journal of Teaching in Physical Education, 36*(4), 388-397. <https://doi.org/:10.1123/jtpe.2016-0192>

Guan, J., Xiang, P., Land, W. M., & Hamilton, X. D. (2023). The Roles of Perceived Physical Education Competence, Enjoyment, and Persistence on Middle School Students’ Physical Activity Engagement. *Perceptual and Motor Skills, 130*(4), 1781-1796. <https://doi.org/:10.1177/00315125231178341>

Guo, H., Schenkelberg, M. A., O’Neill, J. R., Dowda, M., & Pate, R. R. (2018). How Does the Relationship Between Motor Skill Performance and Body Mass Index Impact Physical Activity in Preschool Children? *Pediatric Exercise Science, 30*(2), 266-272. <https://doi.org/:10.1123/pes.2017-0074>

Haapala, E. A., Widlund, A., Poikkeus, A.-M., Lima, R. A., Brage, S., Aunio, P., & Lakka, T. A. (2023). Cross-Lagged Associations between Physical Activity, Motor Performance, and Academic Skills in Primary School Children. *Medicine & Science in Sports & Exercise, 55*(8). Retrieved from <https://journals.lww.com/acsm-msse/fulltext/2023/08000/cross_lagged_associations_between_physical.13.aspx>

Hall, C. J. S., Eyre, E. L. J., Oxford, S. W., & Duncan, M. J. (2018). Relationships between Motor Competence, Physical Activity, and Obesity in British Preschool Aged Children. *Journal of Functional Morphology and Kinesiology, 3*(4). doi:10.3390/jfmk3040057

Hall, C. J. S., Eyre, E. L. J., Oxford, S. W., & Duncan, M. J. (2019). Does Perception of Motor Competence Mediate Associations between Motor Competence and Physical Activity in Early Years Children? *Sports, 7*(4). doi:10.3390/sports7040077

Han, S., Li, B., Meng, S., Li, Y., & Tong, W. (2022). Bi-Directionality between Physical Activity within School and Fundamental Movement Skills in School-Aged Students: A Cross-Lagged Study. *International Journal of Environmental Research and Public Health, 19*(13). doi:10.3390/ijerph19137624

Hands, B., Larkin, D., Parker, H., Straker, L., & Perry, M. (2009). The relationship among physical activity, motor competence and health-related fitness in 14-year-old adolescents. *Scandinavian Journal of Medicine & Science in Sports, 19*(5), 655-663. <https://doi.org/:https://doi.org/10.1111/j.1600-0838.2008.00847.x>

Hardman, C. M., Wanderley, R. d. S., Oliveira, E. S. A. d., & Barros, M. V. G. d. (2017). Relationship between physical activity and BMI with level of motor coordination performance in schoolchildren. *Revista Brasileira de Cineantropometria & Desempenho Humano, 19*, 50-61.

Haugen, T., Ommundsen, Y., & Seiler, S. (2013). The Relationship Between Physical Activity and Physical Self-Esteem in Adolescents: The Role of Physical Fitness Indices. *Pediatric Exercise Science, 25*(1), 138-153. <https://doi.org/:10.1123/pes.25.1.138>

Haugland, E. S., Nilsen, A. K. O., Okely, A. D., Aadland, K. N., & Aadland, E. (2023). Multivariate physical activity association patterns for fundamental motor skills and physical fitness in preschool children aged 3–5 years. *Journal of Sports Sciences, 41*(7), 654-667. <https://doi.org/:10.1080/02640414.2023.2232219>

He, Q., Ng, J. Y. Y., Cairney, J., Bedard, C., & Ha, A. S. C. (2021). Association between Physical Activity and Fundamental Movement Skills in Preschool-Aged Children: Does Perceived Movement Skill Competence Mediate This Relationship? *International Journal of Environmental Research and Public Health, 18*(3). doi:10.3390/ijerph18031289

Hikihara, Y., Watanabe, M., Aoyama, T., Wakabayashi, H., Hanawa, S., Omi, N., & Tanaka, S. (2022). Does earlier acquisition of motor competence promote pubertal physical activity in Japanese elementary school children: A 4-year follow-up study. *Journal of Sports Sciences, 40*(18), 2000-2009. <https://doi.org/:10.1080/02640414.2022.2124710>

Hill, P. J., McNarry, M. A., Lester, L., Foweather, L., Boddy, L. M., Fairclough, S. J., & Mackintosh, K. A. (2022). Sex-Related Differences in the Association of Fundamental Movement Skills and Health and Behavioral Outcomes in Children. *Journal of Motor Learning and Development, 10*(1), 27-40. <https://doi.org/:10.1123/jmld.2020-0066>

Houwen, S., Hartman, E., & Visscher, C. (2009). Physical Activity and Motor Skills in Children with and without Visual Impairments. *Medicine & Science in Sports & Exercise, 41*(1). Retrieved from <https://journals.lww.com/acsm-msse/fulltext/2009/01000/physical_activity_and_motor_skills_in_children.11.aspx>

Hulteen, R. M., Barnett, L. M., Morgan, P. J., Robinson, L. E., Barton, C. J., Wrotniak, B. H., & Lubans, D. R. (2018). Determining the Initial Predictive Validity of the Lifelong Physical Activity Skills Battery. *Journal of Motor Learning and Development, 6*(2), 301-314. <https://doi.org/:10.1123/jmld.2017-0036>

Hulteen, R. M., True, L., & Pfeiffer, K. A. (2020). Differences in associations of product- and process-oriented motor competence assessments with physical activity in children. *Journal of Sports Sciences, 38*(4), 375-382. <https://doi.org/:10.1080/02640414.2019.1702279>

Humble, A., Yu, M.-L., & Brown, T. (2024). Association between parent-proxy-reported and child-self-reported perceptions of children’s motor competence and children’s performance-based motor skill abilities. *Scandinavian Journal of Occupational Therapy, 31*(1), 2274883. <https://doi.org/:10.1080/11038128.2023.2274883>

Hume, C., Okely, A., Bagley, S., Telford, A., Booth, M., Crawford, D., & Salmon, J. (2008). Does Weight Status Influence Associations Between Children's Fundamental Movement Skills and Physical Activity? *Research Quarterly for Exercise and Sport, 79*(2), 158-165. <https://doi.org/:10.1080/02701367.2008.10599479>

Huotari, P., Heikinaro-Johansson, P., Watt, A., & Jaakkola, T. (2018). Fundamental movement skills in adolescents: Secular trends from 2003 to 2010 and associations with physical activity and BMI. *Scandinavian Journal of Medicine & Science in Sports, 28*(3), 1121-1129. <https://doi.org/:https://doi.org/10.1111/sms.13028>

Iivonen, K. S., Sääkslahti, A. K., Mehtälä, A., Villberg, J. J., Tammelin, T. H., Kulmala, J. S., & Poskiparta, M. (2013). Relationship between Fundamental Motor Skills and Physical Activity in 4-Year-Old Preschool Children. *Perceptual and Motor Skills, 117*(2), 627-646. <https://doi.org/:10.2466/10.06.PMS.117x22z7>

Jaakkola, T., Hakonen, H., Kankaanpää, A., Joensuu, L., Kulmala, J., Kallio, J., . . . Tammelin, T. H. (2019a). Longitudinal associations of fundamental movement skills with objectively measured physical activity and sedentariness during school transition from primary to lower secondary school. *Journal of Science and Medicine in Sport, 22*(1), 85-90. <https://doi.org/:https://doi.org/10.1016/j.jsams.2018.07.012>

Jaakkola, T., Huhtiniemi, M., Salin, K., Seppälä, S., Lahti, J., Hakonen, H., & Stodden, D. F. (2019b). Motor competence, perceived physical competence, physical fitness, and physical activity within Finnish children. *Scandinavian Journal of Medicine & Science in Sports, 29*(7), 1013-1021. <https://doi.org/:https://doi.org/10.1111/sms.13412>

Jaakkola, T., & Washington, T. (2013). The relationship between fundamental movement skills and self-reported physical activity during Finnish junior high school. *Physical Education and Sport Pedagogy, 18*(5), 492-505. <https://doi.org/:10.1080/17408989.2012.690386>

Jaakkola, T., Yli-Piipari, S., Huhtiniemi, M., Salin, K., Seppälä, S., Hakonen, H., & Gråstén, A. (2019c). Longitudinal associations among cardiorespiratory and muscular fitness, motor competence and objectively measured physical activity. *Journal of Science and Medicine in Sport, 22*(11), 1243-1248. <https://doi.org/:https://doi.org/10.1016/j.jsams.2019.06.018>

Jaakkola, T., Yli-Piipari, S., Huotari, P., Watt, A., & Liukkonen, J. (2016). Fundamental movement skills and physical fitness as predictors of physical activity: A 6-year follow-up study. *Scandinavian Journal of Medicine & Science in Sports, 26*(1), 74-81. <https://doi.org/:https://doi.org/10.1111/sms.12407>

Jarvis, S., Williams, M., Rainer, P., Jones, E. S., Saunders, J., & Mullen, R. (2018). Interpreting measures of fundamental movement skills and their relationship with health-related physical activity and self-concept. *Measurement in Physical Education and Exercise Science, 22*(1), 88-100. <https://doi.org/:10.1080/1091367X.2017.1391816>

Jekauc, D., Wagner, M. O., Herrmann, C., Hegazy, K., & Woll, A. (2017). Does Physical Self-Concept Mediate the Relationship between Motor Abilities and Physical Activity in Adolescents and Young Adults? *PLOS ONE, 12*(1), e0168539. <https://doi.org/:10.1371/journal.pone.0168539>

Johnson, J. L., Wadsworth, D. D., Rudisill, M. E., Irwin, J. M., & Bridges, C. (2022). Does Skill Performance Influence Young Children’s Perceived Physical Competence? *Perceptual and Motor Skills, 129*(5), 1396-1412. <https://doi.org/:10.1177/00315125221116756>

Kaioglou, V., Dania, A., Kambas, A., & Venetsanou, F. (2023). Associations of Motor Competence, Cardiorespiratory Fitness, and Physical Activity: The Mediating Role of Cardiorespiratory Fitness. *Research Quarterly for Exercise and Sport, 94*(2), 361-367. <https://doi.org/:10.1080/02701367.2021.1991559>

Kalaja, S., Jaakkola, T., Liukkonen, J., & Watt, A. (2010). Fundamental Movement Skills and Motivational Factors Influencing Engagement in Physical Activity. *Perceptual and Motor Skills, 111*(1), 115-128. <https://doi.org/:10.2466/06.10.25.PMS.111.4.115-128>

Kambas, A., Michalopoulou, M., Fatouros, I. G., Christoforidis, C., Manthou, E., Giannakidou, D., . . . Zimmer, R. (2012). The Relationship Between Motor Proficiency and Pedometer-Determined Physical Activity in Young Children. *Pediatric Exercise Science, 24*(1), 34-44. <https://doi.org/:10.1123/pes.24.1.34>

Karuc, J., Mišigoj-Duraković, M., Marković, G., Hadžić, V., Duncan, M. J., Podnar, H., & Sorić, M. (2020). Movement quality in adolescence depends on the level and type of physical activity. *Physical Therapy in Sport, 46*, 194-203. <https://doi.org/:https://doi.org/10.1016/j.ptsp.2020.09.006>

Kavanagh, J., Issartel, J., & Moran, K. (2019). How actual motor competence and perceived motor competence influence motor-skill engagement of a novel cycling task. *Scandinavian Journal of Medicine & Science in Sports, 29*(10), 1583-1590. <https://doi.org/:https://doi.org/10.1111/sms.13492>

Khodaverdi, Z., Bahram, A., Khalaji, H., & Kazemnejad, A. (2013). Motor Skill Competence and Perceived Motor Competence: Which Best Predicts Physical Activity among Girls? *Iranian Journal of Public Health, 42*(10), 1145-1150.

Khodaverdi, Z., Bahram, A., & Robinson, L. E. (2015). Correlates of physical activity behaviours in young Iranian girls. *Child: Care, Health and Development, 41*(6), 903-910. <https://doi.org/:https://doi.org/10.1111/cch.12253>

King-Dowling, S., Proudfoot, N. A., Cairney, J., & Timmons, B. W. (2020). Motor Competence, Physical Activity, and Fitness across Early Childhood. *Medicine & Science in Sports & Exercise, 52*(11). Retrieved from <https://journals.lww.com/acsm-msse/fulltext/2020/11000/motor_competence,_physical_activity,_and_fitness.8.aspx>

Kolunsarka, I., Gråstén, A., Huhtiniemi, M., & Jaakkola, T. (2021). Development of Children’s Actual and Perceived Motor Competence, Cardiorespiratory Fitness, Physical Activity, and BMI. *Medicine & Science in Sports & Exercise, 53*(12). Retrieved from <https://journals.lww.com/acsm-msse/fulltext/2021/12000/development_of_children_s_actual_and_perceived.21.aspx>

Kolunsarka, I., Gråstén, A., Stodden, D., Huhtiniemi, M., & Jaakkola, T. (2023). Impact of Motor Competence Profiles on Adolescents’ Physical Activity and Cardiorespiratory Fitness across Four Years. *Medicine & Science in Sports & Exercise, 55*(9). Retrieved from <https://journals.lww.com/acsm-msse/fulltext/2023/09000/impact_of_motor_competence_profiles_on.11.aspx>

Lalor, A., Brown, T., & Murdolo, Y. (2016). Relationship between children's performance-based motor skills and child, parent, and teacher perceptions of children's motor abilities using self/informant-report questionnaires. *Australian Occupational Therapy Journal, 63*(2), 105-116. <https://doi.org/:https://doi.org/10.1111/1440-1630.12253>

Larouche, R., Boyer, C., Tremblay, M. S., & Longmuir, P. (2013). Physical fitness, motor skill, and physical activity relationships in grade 4 to 6 children. *Applied Physiology, Nutrition, and Metabolism, 39*(5), 553-559. <https://doi.org/:10.1139/apnm-2013-0371>

Larsen, L. R., Kristensen, P. L., Junge, T., Rexen, C. T., & Wedderkopp, N. (2015). Motor Performance as Predictor of Physical Activity in Children: The CHAMPS Study-DK. *Medicine & Science in Sports & Exercise, 47*(9). Retrieved from <https://journals.lww.com/acsm-msse/fulltext/2015/09000/motor_performance_as_predictor_of_physical.11.aspx>

Laukkanen, A., Niemistö, D., Aunola, K., Barnett, L. M., & Sääkslahti, A. (2023). Child perceived motor competence as a moderator between physical activity parenting and child objectively measured physical activity. *Psychology of Sport and Exercise, 67*, 102444. <https://doi.org/:https://doi.org/10.1016/j.psychsport.2023.102444>

Laukkanen, A., Pesola, A., Havu, M., Sääkslahti, A., & Finni, T. (2014). Relationship between habitual physical activity and gross motor skills is multifaceted in 5- to 8-year-old children. *Scandinavian Journal of Medicine & Science in Sports, 24*(2), e102-e110. <https://doi.org/:https://doi.org/10.1111/sms.12116>

Laukkanen, A., Pesola, A. J., Finni, T., & Sääkslahti, A. (2017). Body Mass Index in the Early Years in Relation to Motor Coordination at the Age of 5–7 Years. *Sports, 5*(3). doi:10.3390/sports5030049

LeGear, M., Greyling, L., Sloan, E., Bell, R. I., Williams, B.-L., Naylor, P.-J., & Temple, V. A. (2012). A window of opportunity? Motor skills and perceptions of competence of children in Kindergarten. *International Journal of Behavioral Nutrition and Physical Activity, 9*(1), 29. <https://doi.org/:10.1186/1479-5868-9-29>

Lin, L.-Y., Cherng, R.-J., & Chen, Y.-J. (2017). Relationship between time use in physical activity and gross motor performance of preschool children. *Australian Occupational Therapy Journal, 64*(1), 49-57. <https://doi.org/:https://doi.org/10.1111/1440-1630.12318>

Liong, G. H. E., Ridgers, N. D., & Barnett, L. M. (2015). Associations between Skill Perceptions and Young Children's Actual Fundamental Movement Skills. *Perceptual and Motor Skills, 120*(2), 591-603. <https://doi.org/:10.2466/10.25.PMS.120v18x2>

Lohbeck, A., von Keitz, P., Hohmann, A., & Daseking, M. (2021). Children's Physical Self-Concept, Motivation, and Physical Performance: Does Physical Self-Concept or Motivation Play a Mediating Role? *Frontiers in Psychology, 12*. <https://doi.org/:10.3389/fpsyg.2021.669936>

Lopes, L., Silva Mota, J. A. P., Moreira, C., Abreu, S., Agostinis Sobrinho, C., Oliveira-Santos, J., . . . Santos, R. (2019). Longitudinal associations between motor competence and different physical activity intensities: LabMed physical activity study. *Journal of Sports Sciences, 37*(3), 285-290. <https://doi.org/:10.1080/02640414.2018.1497424>

Lopes, V., Barnett, L., & Rodrigues, L. (2016). Is There an Association Among Actual Motor Competence, Perceived Motor Competence, Physical Activity, and Sedentary Behavior in Preschool Children? *Journal of Motor Learning and Development, 4*(2), 129-141. <https://doi.org/:10.1123/jmld.2015-0012>

Lopes, V. P., & Rodrigues, L. P. (2021). The Role of Physical Fitness on the Relationship Between Motor Competence and Physical Activity: Mediator or Moderator? *Journal of Motor Learning and Development, 9*(3), 456-469. <https://doi.org/:10.1123/jmld.2020-0070>

Lopes, V. P., Saraiva, L., Gonçalves, C., & Rodrigues, L. P. (2018). Association Between Perceived and Actual Motor Competence in Portuguese Children. *Journal of Motor Learning and Development, 6*(s2), S366-S377. <https://doi.org/:10.1123/jmld.2016-0059>

Loucaides, C. A., Chedzoy, S. M., Bennett, N., & Walshe, K. (2004). Correlates of Physical Activity in a Cypriot Sample of Sixth-Grade Children. *Pediatric Exercise Science, 16*(1), 25-36. <https://doi.org/:10.1123/pes.16.1.25>

Ma, F.-F., & Luo, D.-M. (2023). Relationships between physical activity, fundamental motor skills, and body mass index in preschool children. *Frontiers in Public Health, 11*. <https://doi.org/:10.3389/fpubh.2023.1094168>

Matarma, T., Lagström, H., Hurme, S., Tammelin, T. H., Kulmala, J., Barnett, L. M., & Koski, P. (2018). Motor skills in association with physical activity, sedentary time, body fat, and day care attendance in 5-6-year-old children—The STEPS Study. *Scandinavian Journal of Medicine & Science in Sports, 28*(12), 2668-2676. <https://doi.org/:https://doi.org/10.1111/sms.13264>

McGrane, B., Belton, S., Powell, D., & Issartel, J. (2017). The relationship between fundamental movement skill proficiency and physical self-confidence among adolescents. *Journal of Sports Sciences, 35*(17), 1709-1714. <https://doi.org/:10.1080/02640414.2016.1235280>

McGrane, B., Powell, D., Belton, S., & Issartel, J. (2018). Investigation Into the Relationship Between Adolescents’ Perceived and Actual Fundamental Movement Skills and Physical Activity. *Journal of Motor Learning and Development, 6*(s2), S424-S439. <https://doi.org/:10.1123/jmld.2016-0073>

McIntyre, F., Parker, H., Chivers, P., & Hands, B. (2018). Actual competence, rather than perceived competence, is a better predictor of physical activity in children aged 6-9 years. *Journal of Sports Sciences, 36*(13), 1433-1440. <https://doi.org/:10.1080/02640414.2017.1390243>

Melby, P. S., Elsborg, P., Nielsen, G., Lima, R. A., Bentsen, P., & Andersen, L. B. (2021). Exploring the importance of diversified physical activities in early childhood for later motor competence and physical activity level: a seven-year longitudinal study. *BMC Public Health, 21*(1), 1492. <https://doi.org/:10.1186/s12889-021-11343-1>

Menescardi, C., De Meester, A., Álvarez, O., Castillo, I., Haerens, L., & Estevan, I. (2023). The mediational role of motivation in the model of motor development in childhood: A longitudinal study. *Psychology of Sport and Exercise, 66*, 102398. <https://doi.org/:https://doi.org/10.1016/j.psychsport.2023.102398>

Menescardi, C., & Estevan, I. (2021). Parental and Peer Support Matters: A Broad Umbrella of the Role of Perceived Social Support in the Association between Children’s Perceived Motor Competence and Physical Activity. *International Journal of Environmental Research and Public Health, 18*(12). doi:10.3390/ijerph18126646

Miller, A., Eather, N., Duncan, M., & Lubans, D. R. (2019). Associations of object control motor skill proficiency, game play competence, physical activity and cardiorespiratory fitness among primary school children. *Journal of Sports Sciences, 37*(2), 173-179. <https://doi.org/:10.1080/02640414.2018.1488384>

Monacis, D., Trecroci, A., Invernizzi, P. L., & Colella, D. (2022). Can Enjoyment and Physical Self-Perception Mediate the Relationship between BMI and Levels of Physical Activity? Preliminary Results from the Regional Observatory of Motor Development in Italy. *International Journal of Environmental Research and Public Health, 19*(19). doi:10.3390/ijerph191912567

Morano, M., Bortoli, L., Ruiz, M. C., Campanozzi, A., & Robazza, C. (2020). Actual and perceived motor competence: Are children accurate in their perceptions? *PLOS ONE, 15*(5), e0233190. <https://doi.org/:10.1371/journal.pone.0233190>

Morano, M., Colella, D., Robazza, C., Bortoli, L., & Capranica, L. (2011). Physical self-perception and motor performance in normal-weight, overweight and obese children. *Scandinavian Journal of Medicine & Science in Sports, 21*(3), 465-473. <https://doi.org/:https://doi.org/10.1111/j.1600-0838.2009.01068.x>

Morgan, P. J., Okely, A. D., Cliff, D. P., Jones, R. A., & Baur, L. A. (2008). Correlates of Objectively Measured Physical Activity in Obese Children. *Obesity, 16*(12), 2634-2641. <https://doi.org/:https://doi.org/10.1038/oby.2008.463>

Morrison, K. M., Cairney, J., Eisenmann, J., Pfeiffer, K., & Gould, D. (2018). Associations of Body Mass Index, Motor Performance, and Perceived Athletic Competence with Physical Activity in Normal Weight and Overweight Children. *Journal of Obesity, 2018*, 3598321. <https://doi.org/:10.1155/2018/3598321>

Nicolai Ré, A. H., Okely, A. D., Logan, S. W., da Silva, M. M. L. M., Cattuzzo, M. T., & Stodden, D. F. (2020). Relationship between meeting physical activity guidelines and motor competence among low-income school youth. *Journal of Science and Medicine in Sport, 23*(6), 591-595. <https://doi.org/:https://doi.org/10.1016/j.jsams.2019.12.014>

Niemistö, D., Barnett, L. M., Cantell, M., Finni, T., Korhonen, E., & Sääkslahti, A. (2019). Socioecological correlates of perceived motor competence in 5- to 7-year-old Finnish children. *Scandinavian Journal of Medicine & Science in Sports, 29*(5), 753-765. <https://doi.org/:https://doi.org/10.1111/sms.13389>

Nilsen, A. K. O., Anderssen, S. A., Johannessen, K., Aadland, K. N., Ylvisaaker, E., Loftesnes, J. M., & Aadland, E. (2020a). Bi-directional prospective associations between objectively measured physical activity and fundamental motor skills in children: a two-year follow-up. *International Journal of Behavioral Nutrition and Physical Activity, 17*(1), 1. <https://doi.org/:10.1186/s12966-019-0902-6>

Nilsen, A. K. O., Anderssen, S. A., Loftesnes, J. M., Johannessen, K., Ylvisaaker, E., & Aadland, E. (2020b). The multivariate physical activity signature associated with fundamental motor skills in preschoolers. *Journal of Sports Sciences, 38*(3), 264-272. <https://doi.org/:10.1080/02640414.2019.1694128>

Noonan, R. J., Boddy, L. M., Knowles, Z. R., & Fairclough, S. J. (2018). Predisposing, reinforcing and enabling factors for physical activity in boys and girls from socially disadvantaged communities. *Health Education Journal, 78*(2), 149-162. <https://doi.org/:10.1177/0017896918792690>

Norman, C., Geer, W., & Looper, J. (2019). Motor competency and physical activity in elementary school aged children who participate in nontraditional sports *Journal of Physical Education and Sport, 19*(2).

O’ Brien, W., Belton, S., & Issartel, J. (2016). The relationship between adolescents’ physical activity, fundamental movement skills and weight status. *Journal of Sports Sciences, 34*(12), 1159-1167. <https://doi.org/:10.1080/02640414.2015.1096017>

Paxton, R. J., Estabrooks, P. A., & Dzewaltowski, D. (2004). Attraction to Physical Activity Mediates the Relationship between Perceived Competence and Physical Activity in Youth. *Research Quarterly for Exercise and Sport, 75*(1), 107-111. <https://doi.org/:10.1080/02701367.2004.10609139>

Peers, C., Issartel, J., Behan, S., O'Connor, N., & Belton, S. (2020). Movement competence: Association with physical self-efficacy and physical activity. *Human Movement Science, 70*, 102582. <https://doi.org/:https://doi.org/10.1016/j.humov.2020.102582>

Pereira, S., Reyes, A., Moura-Dos-Santos, M. A., Santos, C., Gomes, T. N., Tani, G., . . . Maia, J. (2020). Why are children different in their moderate-to-vigorous physical activity levels? A multilevel analysis. *Jornal de Pediatria, 96*(2), 225-232. <https://doi.org/:https://doi.org/10.1016/j.jped.2018.10.013>

Pérez, L. M. R., & Sanz, J. L. G. (2005). New Measure of Perceived Motor Competence for Children Ages 4 to 6 Years. *Perceptual and Motor Skills, 101*(1), 131-148. <https://doi.org/:10.2466/pms.101.1.131-148>

Pesce, C., Masci, I., Marchetti, R., Vannozzi, G., & Schmidt, M. (2018). When Children’s Perceived and Actual Motor Competence Mismatch: Sport Participation and Gender Differences. *Journal of Motor Learning and Development, 6*(s2), S440-S460. <https://doi.org/:10.1123/jmld.2016-0081>

Queiroz, D. d. R., Aguilar, J. A., Martins Guimarães, T. G., Hardman, C. M., Lima, R. A., Duncan, M. J., . . . de Barros, M. V. G. (2020). Association between body mass index, physical activity and motor competence in children: moderation analysis by different environmental contexts. *Annals of Human Biology, 47*(5), 417-424. <https://doi.org/:10.1080/03014460.2020.1779815>

Raudsepp, L., Liblik, R., & Hannus, A. (2002). Children’s and Adolescents’ Physical Self-Perceptions as Related to Moderate to Vigorous Physical Activity and Physical Fitness. *Pediatric Exercise Science, 14*(1), 97-106. <https://doi.org/:10.1123/pes.14.1.97>

Raudsepp, L., & Päll, P. (2006). The Relationship between Fundamental Motor Skills and Outside-School Physical Activity of Elementary School Children. *Pediatric Exercise Science, 18*(4), 426-435. <https://doi.org/:10.1123/pes.18.4.426>

Reed, J. A., Metzker, A., & Phillips, D. A. (2004). Relationships between Physical Activity and Motor Skills in Middle School Children. *Perceptual and Motor Skills, 99*(2), 483-494. <https://doi.org/:10.2466/pms.99.2.483-494>

Reyes, A. C., Chaves, R., Baxter-Jones, A. D. G., Vasconcelos, O., Barnett, L. M., Tani, G., . . . Maia, J. (2019). Modelling the dynamics of children’s gross motor coordination. *Journal of Sports Sciences, 37*(19), 2243-2252. <https://doi.org/:10.1080/02640414.2019.1626570>

Robinson, L. E. (2011). The relationship between perceived physical competence and fundamental motor skills in preschool children. *Child: Care, Health and Development, 37*(4), 589-596. <https://doi.org/:https://doi.org/10.1111/j.1365-2214.2010.01187.x>

Robinson, L. E., & Palmer, K. K. (2021). Examining the psychometric properties of the digital scale of perceived motor competence in young children. *Scandinavian Journal of Medicine & Science in Sports, 31*(12), 2272-2281. <https://doi.org/:https://doi.org/10.1111/sms.14042>

Robinson, L. E., Wadsworth, D. D., & Peoples, C. M. (2012). Correlates of School-Day Physical Activity in Preschool Students. *Research Quarterly for Exercise and Sport, 83*(1), 20-26. <https://doi.org/:10.1080/02701367.2012.10599821>

Rogers, V., Barnett, L. M., & Lander, N. (2018). The Relationship Between Fundamental Movement Skills and Physical Self-Perception Among Adolescent Girls. *Journal of Motor Learning and Development, 6*(s2), S378-S390. <https://doi.org/:10.1123/jmld.2017-0041>

Rudisill, M. E., Mahar, M. T., & Meaney, K. S. (1993). The Relationship between Children's Perceived and Actual Motor Competence. *Perceptual and Motor Skills, 76*(3), 895-906. <https://doi.org/:10.2466/pms.1993.76.3.895>

Ryu, S., Lee, J. E., Liu, W., McDonough, D. J., & Gao, Z. (2021a). Investigating Relationships between Preschool Children’s Perceived Competence, Motor Skills, and Physical Activity: A Cross-Lagged Panel Model. *Journal of Clinical Medicine, 10*(23). doi:10.3390/jcm10235620

Ryu, S., Lee, J. E., Zeng, N., Stodden, D., McDonough, D. J., Liu, W., & Gao, Z. (2021b). Bidirectional Relationships among Children’s Perceived Competence, Motor Skill Competence, Physical Activity, and Cardiorespiratory Fitness across One School Year. *BioMed Research International, 2021*, 1704947. <https://doi.org/:10.1155/2021/1704947>

Sabiston, C. M., & Crocker, P. R. E. (2008). Examining an Integrative Model of Physical Activity and Healthy Eating Self-Perceptions and Behaviors Among Adolescents. *Journal of Adolescent Health, 42*(1), 64-72. <https://doi.org/:https://doi.org/10.1016/j.jadohealth.2007.08.005>

Sallen, J., Andrä, C., Ludyga, S., Mücke, M., & Herrmann, C. (2020). School Children’s Physical Activity, Motor Competence, and Corresponding Self-Perception: A Longitudinal Analysis of Reciprocal Relationships. *Journal of Physical Activity and Health, 17*(11), 1083-1090. <https://doi.org/:10.1123/jpah.2019-0507>

Sallis, J. F., Alcaraz, J. E., McKenzie, T. L., & Hovell, M. F. (1999). Predictors of change in children’s physical activity over 20 months: Variations by gender and level of adiposity. *American Journal of Preventive Medicine, 16*(3), 222-229. <https://doi.org/:https://doi.org/10.1016/S0749-3797(98)00154-8>

Santos, M. O., Barbosa, D. G., Junior, G. J. F., Silva, R. C., Pelegrini, A., & Felden, É. P. G. (2018). Capacity of Objective Measures of Physical Activity to Predict Brazilian Children’s Low Motor Proficiency. *Perceptual and Motor Skills, 125*(4), 669-681. <https://doi.org/:10.1177/0031512518774993>

Schmutz, E. A., Leeger-Aschmann, C. S., Kakebeeke, T. H., Zysset, A. E., Messerli-Bürgy, N., Stülb, K., . . . Kriemler, S. (2020). Motor Competence and Physical Activity in Early Childhood: Stability and Relationship. *Frontiers in Public Health, 8*. <https://doi.org/:10.3389/fpubh.2020.00039>

Seabra, A. C., Seabra, A. F., Mendonça, D. M., Brustad, R., Maia, J. A., Fonseca, A. M., & Malina, R. M. (2013). Psychosocial correlates of physical activity in school children aged 8–10 years. *European Journal of Public Health, 23*(5), 794-798. <https://doi.org/:10.1093/eurpub/cks149>

Shull, E. R., Dowda, M., McIver, K. L., McLain, A. C., Benjamin-Neelon, S. E., Ulrich, B., & Pate, R. R. (2022). Behavioral, Environmental, and Demographic Factors Associated with Objectively Measured Physical Activity in Infants. *Childhood Obesity, 18*(7), 466-475. <https://doi.org/:10.1089/chi.2021.0197>

Silva-Santos, S., Santos, A., Duncan, M., Vale, S., & Mota, J. (2019). Association Between Moderate and Vigorous Physical Activity and Gross Motor Coordination in Preschool Children. *Journal of Motor Learning and Development, 7*(2), 273-285. <https://doi.org/:10.1123/jmld.2017-0056>

Silva-Santos, S., Santos, A., Martins, C., Duncan, M., Lagoa, M. J., Vale, S., & Mota, J. (2021). Associations Between Motor Competence, Moderate-to-Vigorous Physical Activity, and Body Mass Index Among Preschoolers Over 1 Year. *Journal of Physical Activity and Health, 18*(7), 832-837. <https://doi.org/:10.1123/jpah.2020-0356>

Slykerman, S., Ridgers, N. D., Stevenson, C., & Barnett, L. M. (2016). How important is young children's actual and perceived movement skill competence to their physical activity? *Journal of Science and Medicine in Sport, 19*(6), 488-492. <https://doi.org/:https://doi.org/10.1016/j.jsams.2015.07.002>

Smith, E., Fazeli, F., Wilkinson, K., & Clark, C. C. T. (2021). Physical behaviors and fundamental movement skills in British and Iranian children: An isotemporal substitution analysis. *Scandinavian Journal of Medicine & Science in Sports, 31*(2), 398-404. <https://doi.org/:https://doi.org/10.1111/sms.13837>

Spessato, B. C., Gabbard, C., Robinson, L., & Valentini, N. C. (2013). Body mass index, perceived and actual physical competence: the relationship among young children. *Child: Care, Health and Development, 39*(6), 845-850. <https://doi.org/:https://doi.org/10.1111/cch.12014>

Spessato, B. C., Gabbard, C., & Valentini, N. C. (2013). The Role of Motor Competence and Body Mass Index in Children’s Activity Levels in Physical Education Classes. *Journal of Teaching in Physical Education, 32*(2), 118-130. <https://doi.org/:10.1123/jtpe.32.2.118>

Stein, C., Fisher, L., Berkey, C., & Colditz, G. (2007). Adolescent Physical Activity and Perceived Competence: Does Change in Activity Level Impact Self-Perception? *Journal of Adolescent Health, 40*(5), 462.e461-462.e468. <https://doi.org/:https://doi.org/10.1016/j.jadohealth.2006.11.147>

Strotmeyer, A., Herrmann, C., & Kehne, M. (2022). A longitudinal analysis of reciprocal relationships between actual and perceived motor competencies and physical self-concept in primary-school age children. *Psychology of Sport and Exercise, 63*, 102269. <https://doi.org/:https://doi.org/10.1016/j.psychsport.2022.102269>

Sung, Y.-S., Loh, S. C., & Lin, L.-Y. (2021). Physical activity and motor performance: A comparison between young children with and without autism spectrum disorder. *Neuropsychiatric disease and treatment*, 3743-3751.

Syväoja, H. J., Kankaanpää, A., Hakonen, H., Inkinen, V., Kulmala, J., Joensuu, L., . . . Tammelin, T. H. (2021). How physical activity, fitness, and motor skills contribute to math performance: Working memory as a mediating factor. *Scandinavian Journal of Medicine & Science in Sports, 31*(12), 2310-2321. <https://doi.org/:https://doi.org/10.1111/sms.14049>

Telford, R. M., Telford, R. D., Olive, L. S., Cochrane, T., & Davey, R. (2016). Why Are Girls Less Physically Active than Boys? Findings from the LOOK Longitudinal Study. *PLOS ONE, 11*(3), e0150041. <https://doi.org/:10.1371/journal.pone.0150041>

Temple, V. A., Crane, J. R., Brown, A., Williams, B.-L., & Bell, R. I. (2016). Recreational activities and motor skills of children in kindergarten. *Physical Education and Sport Pedagogy, 21*(3), 268-280. <https://doi.org/:10.1080/17408989.2014.924494>

Tietjens, M., Barnett, L. M., Dreiskämper, D., Holfelder, B., Utesch, T. O., Lander, N., . . . Schott, N. (2020). Conceptualising and testing the relationship between actual and perceived motor performance: A cross-cultural comparison in children from Australia and Germany. *Journal of Sports Sciences, 38*(17), 1984-1996. <https://doi.org/:10.1080/02640414.2020.1766169>

Tietjens, M., Dreiskaemper, D., Utesch, T., Schott, N., Barnett, L. M., & Hinkley, T. (2018). Pictorial Scale of Physical Self-Concept for Younger Children (P-PSC-C): A Feasibility Study. *Journal of Motor Learning and Development, 6*(s2), S391-S402. <https://doi.org/:10.1123/jmld.2016-0088>

Toftegaard-Stoeckel, J., Groenfeldt, V., & Andersen, L. B. (2010). Children's self-perceived bodily competencies and associations with motor skills, body mass index, teachers' evaluations, and parents' concerns. *Journal of Sports Sciences, 28*(12), 1369-1375. <https://doi.org/:10.1080/02640414.2010.510845>

True, L., Brian, A., Goodway, J., & Stodden, D. (2017). Relationships between product-and process-oriented measures of motor competence and perceived competence. *Journal of Motor Learning and Development, 5*(2), 319-335. <https://doi.org/:10.1123/jmld.2016-0042>

Tsuda, E., Goodway, J. D., Famelia, R., & Brian, A. (2020). Relationship Between Fundamental Motor Skill Competence, Perceived Physical Competence and Free-Play Physical Activity in Children. *Research Quarterly for Exercise and Sport, 91*(1), 55-63. <https://doi.org/:10.1080/02701367.2019.1646851>

Valentini, N. C., Nobre, G. C., de Souza, M. S., & Duncan, M. J. (2020). Are BMI, Self-Perceptions, Motor Competence, Engagement, and Fitness Related to Physical Activity in Physical Education Lessons? *Journal of Physical Activity and Health, 17*(5), 493-500. <https://doi.org/:10.1123/jpah.2019-0532>

Valentini, N. C., Souza, P. S. d., Souza, M. S. d., & Nobre, G. C. (2023). Individual and environmental parameters in children with and without developmental coordination disorder: associations with physical activity and body mass index. *Frontiers in Pediatrics, 11*. <https://doi.org/:10.3389/fped.2023.1202488>

van Niekerk, L.-L., du Toit, D., & Pienaar, A. E. (2016). The correlation between motor proficiency and physical activity in Senior Phase learners in the Potchefstroom area. *Health SA Gesondheid, 21*, 348-355. <https://doi.org/:https://doi.org/10.1016/j.hsag.2016.05.001>

Vedul-Kjelsås, V., Sigmundsson, H., Stensdotter, A. K., & Haga, M. (2012). The relationship between motor competence, physical fitness and self-perception in children. *Child: Care, Health and Development, 38*(3), 394-402. <https://doi.org/:https://doi.org/10.1111/j.1365-2214.2011.01275.x>

Veldman, S. L. C., Jones, R. A., Santos, R., Sousa-Sá, E., Pereira, J. R., Zhang, Z., & Okely, A. D. (2018). Associations between gross motor skills and physical activity in Australian toddlers. *Journal of Science and Medicine in Sport, 21*(8), 817-821. <https://doi.org/:https://doi.org/10.1016/j.jsams.2017.12.007>

Visagie, M., Coetzee, D., & Pienaar, A. E. (2017). Activity preferences of 9-to 10-year-old girls and the relationship between object control skills and physical activity levels: the NW-Child Study. *South African Journal for Research in Sport, Physical Education and Recreation, 39*(1), 199-217.

Visser, E. L., Mazzoli, E., Hinkley, T., Lander, N. J., Utesch, T., & Barnett, L. M. (2020). Are children with higher self-reported wellbeing and perceived motor competence more physically active? A longitudinal study. *Journal of Science and Medicine in Sport, 23*(3), 270-275. <https://doi.org/:https://doi.org/10.1016/j.jsams.2019.09.005>

Wang, C. K. J., Chia, Y. H. M., Quek, J. J., & Liu, W. C. (2006). Patterns of physical activity, sedentary behaviors, and psychological determinants of physical activity among Singaporean school children. *International Journal of Sport and Exercise Psychology, 4*(3), 227-249. <https://doi.org/:10.1080/1612197X.2006.9671797>

Webb, O. J., Benjamin, C. C., Gammon, C., McKee, H. C., & Biddle, S. J. H. (2013). Physical activity, sedentary behaviour and physical self-perceptions in adolescent girls: A mediation analysis. *Mental Health and Physical Activity, 6*(1), 24-29. <https://doi.org/:https://doi.org/10.1016/j.mhpa.2012.08.005>

Weedon, B. D., Esser, P., Collett, J., Izadi, H., Joshi, S., Meaney, A., . . . Dawes, H. (2023). The Relationship Between Motor Competence Physical Activity Cardiorespiratory Fitness and BMI in UK Adolescents. *Research Quarterly for Exercise and Sport*, 1-7. <https://doi.org/:10.1080/02701367.2023.2265442>

Welk, G. J., & Schaben, J. A. (2004). Psychosocial Correlates of Physical Activity in Children-A Study of Relationships When Children Have Similar Opportunities to Be Active. *Measurement in Physical Education and Exercise Science, 8*(2), 63-81. <https://doi.org/:10.1207/s15327841mpee0802_2>

Wrotniak, B. H., Epstein, L. H., Dorn, J. M., Jones, K. E., & Kondilis, V. A. (2006). The Relationship Between Motor Proficiency and Physical Activity in Children. *Pediatrics, 118*(6), e1758-e1765. <https://doi.org/:10.1542/peds.2006-0742>

Yli-Piipari, S., Gråstén, A., Huhtiniemi, M., Salin, K., & Jaakkola, T. (2021). One-year stability of physical education-centered physical literacy indicators on objectively measured physical activity. *European Physical Education Review, 28*(2), 361-379. <https://doi.org/:10.1177/1356336X211046302>

Zeng, N., Johnson, S. L., Boles, R. E., & Bellows, L. L. (2019). Social-ecological correlates of fundamental movement skills in young children. *Journal of Sport and Health Science, 8*(2), 122-129. <https://doi.org/:https://doi.org/10.1016/j.jshs.2019.01.001>

Zhang, T., Lee, J., Chu, T. L., Chen, C., & Gu, X. (2020). Accessing Physical Activity and Health Disparities among Underserved Hispanic Children: The Role of Actual and Perceived Motor Competence. *International Journal of Environmental Research and Public Health, 17*(9). doi:10.3390/ijerph17093013

Zhang, T., Thomas, K., & Weiller, K. (2015). Predicting Physical Activity in 10-12 Year Old Children: A Social Ecological Approach. *Journal of Teaching in Physical Education, 34*(3), 517-536. <https://doi.org/:10.1123/jtpe.2013-0195>

Ziviani, J., MacDonald, D., Jenkins, D., Rodger, S., Batch, J., & Cerin, E. (2006). Physical Activity of Young Children. *OTJR: Occupational Therapy Journal of Research, 26*(1), 4-14. <https://doi.org/:10.1177/153944920602600102>
