# Supplementary material for: Children's and Adolescents’ Actual Motor Competence, Perceived Physical Competence and Physical Activity: A Structural Equation Modelling Meta-Analysis
Source: Sports Med. 2025 May 6;55(8):1923–36. doi: 10.1007/s40279-025-02233-2 (PMC12460483; doi:10.1007/s40279-025-02233-2)
Supplement: Supplementary file 4 — Supplementary file4 (DOCX 23 KB) [file 40279_2025_2233_MOESM4_ESM.docx]

Table D1 – Results from sensitivity analysis comparing results based on the assessment of actual motor competence, perceived motor competence, and physical activity

|  | **k samples** | **n participants** | **a** | **b** | **c'** | **ab** |
| --- | --- | --- | --- | --- | --- | --- |
| **Gross motor competence** |  |  |  |  |  |  |
| ***Actual competence assessment*** |  |  |  |  |  |  |
| Process | 88 | 17,125 | .204  (.154, .252 | .126  (.069, .182) | .173  (.014, .209 | .026  (.014, .039) |
| Product | 51 | 17,722 | .242  (.171, .310) | .105  (-.003, .209) | .192  (.139, .245) | .025  (-.001, .044) |
| ***Perceived competence assessment*** |  |  |  |  |  |  |
| FMS | 32 | 5384 | .159  (.058, .256) | .160  (.095, .219) | .098  (.024, .162) | .025  (.001, .045) |
| Other physical competence assessment | 90 | 35,517 | .251  (.216, .283) | .165  (.120, .209) | .184  (.125, .241) | .041  (.030, .054) |
| ***Physical activity assessment*** |  |  |  |  |  |  |
| Device | 89 | 18,896 | .211  (.136, .277) | .093  (.041, .146) | .177  (.146, .208) | .019  (.009, .033) |
| Self-reported | 63 | 33,387 | .202  .139, .263) | .212  .167, .257) | .178  (.123, .232) | .043  (.029, .058) |
| **Locomotion** |  |  |  |  |  |  |
| ***Actual competence assessment*** |  |  |  |  |  |  |
| Process | 90 | 20,940 | .102  (.038, .166) | .130  (.075, .185) | .148  (.097, .199) | .013  (.005, .024) |
| Product | 74 | 22,234 | .276  (.198, .353) | .174  (.052, .290) | .076  (.007, .144) | .048  (.015, .061) |
| ***Perceived competence assessment*** |  |  |  |  |  |  |
| FMS | 37 | 7890 | .133  (.054, .212) | .160  (.100, .215) | .037  (-.021, .158) | .021  (.008, .038) |
| Other physical competence assessment | 90 | 36,889 | .192  (.118, .266) | .166  (.120 .212) | .207  (.26, .289) | .032  (.020, .046) |
| ***Physical activity assessment*** |  |  |  |  |  |  |
| Device | 109 | 25,950 | .124  (.028, .219) | .115  (.064, .165) | .132  (.092, .171) | .014  (.003, .027) |
| Self-reported | 66 | 31,788 | .097  (-.142, .159) | .238  (.188, .286) | .096  (-.033, .219) | .023  (-0.11, .044) |
| **Object control** |  |  |  |  |  |  |
| ***Actual competence assessment*** |  |  |  |  |  |  |
| Process | 109 | 23,401 | .185  (.131, .238) | .148  (.097, .198) | .132  (.087, .178) | .027  (.017, .040) |
| Product | 52 | 14,012 | .134  (.032, .239) | .114  (-.002, .207) | .180  (.121, .237) | .015  (-.001, .038) |
| ***Perceived competence assessment*** |  |  |  |  |  |  |
| FMS | 43 | 8332 | .141  (.077, 0204) | .176  (.121, .228) | .062  (.007, .131) | .025  (.013, .040) |
| Other physical competence assessment | 88 | 34,053 | .213  (.141, .287) | .166  (.117, .213) | .205  (.104, .306) | .035  (.023, .051) |
| ***Physical activity assessment*** |  |  |  |  |  |  |
| Device | 108 | 21,689 | .178  (.099, .259) | .111  (.063, .158) | .159  (.119, .200) | .020  (.011, .032) |
| Self-reported | 64 | 32,335 | .124  (-.011, .135) | .231  (.186, .276) | .115  (.044, .185) | .029  (-.002, .059) |
